# Design, Synthesis, and Biological Evaluation of Pyrano[2,3-c]-pyrazole–Based RalA Inhibitors Against Hepatocellular Carcinoma

**DOI:** 10.3389/fchem.2021.700956

**Published:** 2021-11-15

**Authors:** Yuting Wang, Mingyao He, Xiang Li, Jinlong Chai, Qinglin Jiang, Cheng Peng, Gu He, Wei Huang

**Affiliations:** ^1^ State Key Laboratory of Southwestern Chinese Medicine Resources, Hospital of Chengdu University of Traditional Chinese Medicine, School of Pharmacy, Chengdu University of Traditional Chinese Medicine, Chengdu, China; ^2^ State Key Laboratory of Biotherapy and Department of Urology, West China Hospital, Sichuan University, Chengdu, China; ^3^ School of Pharmacy and Sichuan Province College Key Laboratory of Structure-Specific Small Molecule Drugs, Chengdu Medical College, Chengdu, China

**Keywords:** RalA inhibitors, pyrano[2;3-c]-pyrazole, hepatocellular carcinoma, synthesis, autophagy

## Abstract

The activation of Ras small GTPases, including RalA and RalB, plays an important role in carcinogenesis, tumor progress, and metastasis. In the current study, we report the discovery of a series of 6-sulfonylamide-pyrano [2,3-c]-pyrazole derivatives as novel RalA inhibitors. ELISA-based biochemical assay results indicated that compounds **4k**–**4r** suppressed RalA/B binding capacities to their substrates. Cellular proliferation assays indicated that these RalA inhibitors potently inhibited the proliferation of HCC cell lines, including HepG2, SMMC-7721, Hep3B, and Huh-7 cells. Among the evaluated compounds, **4p** displayed good inhibitory capacities on RalA (IC_50_ = 0.22 μM) and HepG2 cells (IC_50_ = 2.28 μM). Overall, our results suggested that a novel small-molecule RalA inhibitor with a 6-sulfonylamide-pyrano [2, 3-c]-pyrazole scaffold suppressed autophagy and cell proliferation in hepatocellular carcinoma, and that it has potential for HCC-targeted therapy.

## Introduction

Hepatocellular carcinoma (HCC) is a primary liver malignancy with one of the highest mortality rates worldwide (Jacobson et al., 2018; Zhou et al., 2018; Paludetto et al., 2019; Siegel et al., 2019). Although significant progress has been made to improve chemotherapy, transcatheter artery chemoembolization (TACE), and targeted HCC therapy, many patients still present recurrences and the development of drug resistance (Xue et al., 2019; Pan et al., 2020; Yu et al., 2020). Therefore, novel targeted therapeutic approaches are required to help overcome these problems. From a molecular-level perspective, an important HCC development factor is the imbalance of major signaling pathways, including Ras, p53, PI3K/Akt, and Wnt/β-catenin (Saha and Giri, 2019; Zhongqi et al., 2019). Ras—a small-molecule GTPase member—has important regulatory functions in cell signal transduction, cytoplasmic skeletal construction, and material transport (Ostrem et al., 2013; Lim et al., 2014; Lito et al., 2016). Notably, recent findings indicated that the Ras gene was mutated in more than 30% of HCC-afflicted patients sampled (Guichard et al., 2012; Lindsay and Blackhall, 2019; Suzuki et al., 2021). Mutations in the Ras gene have also been detected in various other types of cancers as well, including those for lung cancer, pancreatic cancer, and colon cancer (Zhang et al., 2018; Zhao et al., 2018; Chen et al., 2020). In the subseries of Ras signaling pathways, Ral has one of the most similar structures and functions compared to Ras, reaching up to 50% similarity for their sequences (Yan and Theodorescu, 2018). In the Ral small-GTPase branch, RalA and RalB are closely related G proteins with similar protein structures (Jiang et al., 2016). Their protein structures have an N-terminal free-drifting 11 amino acid sequence, a C-terminal membrane-targeting sequence, and a GTP- binding domain, which facilitate GDP/GTP binding (Zheng et al., 2016). The GTP- binding domain consists of five α-helices, six β-helices, and five loops. Their GTPase activity is low and requires the involvement of GTPase-activating proteins (GAPs) (Tracy et al., 2016). Additionally, the guanylate exchange factor (GEF) plays an important role in GTP/GDP binding and release (Hobbs et al., 2016). Usually, functional Ral members depend on their ability to cycle between activated (phosphorylated) and inactivated (dephosphorylated) Ral states. The Ral inhibitors can be divided into the following cases: targeted to Ral guanine exchange factors (RalGEFs); directly targeted to Ral; targeted to Ral effectors, and combinations of these therapies (Klose et al., 2016).

**FIGURE 1:**
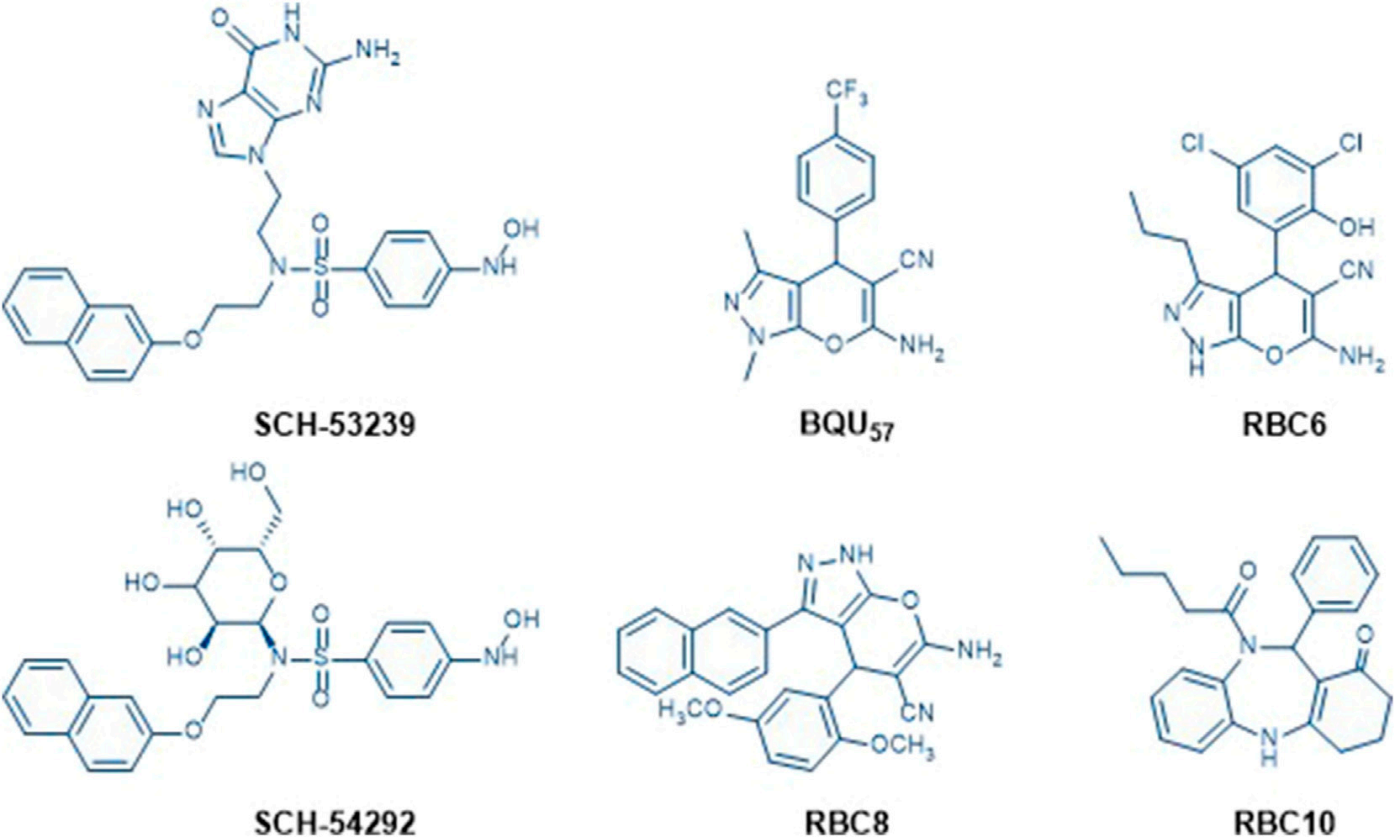
Chemical structures of known Ral inhibitors, including SCH-53239, SCH-54292, BQU57, RBC6, RBC8, and RBC10.

SCH-53239 was originally designed to inhibit guanine nucleotide exchange and control Ral signaling. A subsequent study elucidated the structure of SCH-53239 and led to the discovery of a derivative with a higher water solubility known as SCH-54292 (Gray et al., 2020). RBC8 has an efficient inhibitory effect on human and mouse platelets, preventing interactions with effector proteins and Ral-binding protein by binding to allosteric sites on GDP-bound Rals, leaving it in an inactive state. These types of compounds include currently reported Ral inhibitors, such as RBC6, RBC10, and BQU5726-27 (Figure 1). Prior structure analyses of BQU57 and RBC8 have suggested that the pyrano-fused pyrazole scaffold can bind to the RalA-GDP allosteric site (Alvarado and Giles, 2007; Hamada et al., 2011; Ezzeldin et al., 2014; Yan et al., 2016; Yan and Theodorescu, 2018; Walsh et al., 2019; Bum-Erdene et al., 2020; Xiong et al., 2020). In the current study, following a rationalized approach for strategic drug design, we aimed to develop methods to change the pyrazole ring N-methyl group into a substituted phenyl ring and add a sulfonyl group at the pyrano ring 2-amine group. These adjustments were performed to enhance RalA binding, via a 3-cyanide group and 4-aronmatic ring. The resultant pyrano[2,3-c]-pyrazole derivatives were assayed via their RalA and HCC cellular proliferation inhibitory capacities.

To achieve these objectives we designed and synthesized 6-sulfonamido-pyrano[2,3-c]-pyrazole–based RalA inhibitors. Then, we assayed their performance in HCC-based models *in vitro* and *in vivo*. We used HepG2 cells as vectors for *in vitro* ELISA-based biochemical assays, cell proliferation assays, and autophagy assays. We hypothesized that compound **4p** would suppress RalA/B activation and cellular proliferation, as well as subsequently induce lethal autophagy in HCC-afflicted cells. Furthermore, we hypothesized that compound **4p** would significantly suppress tumor growth using a HepG2 xenograft–based model. Overall, we expected that our findings and novel approach using RalA inhibitors will stimulate novel research that might achieve improved outcomes for targeted HCC therapies.

## Results and Discussion

**FIGURE 2:**
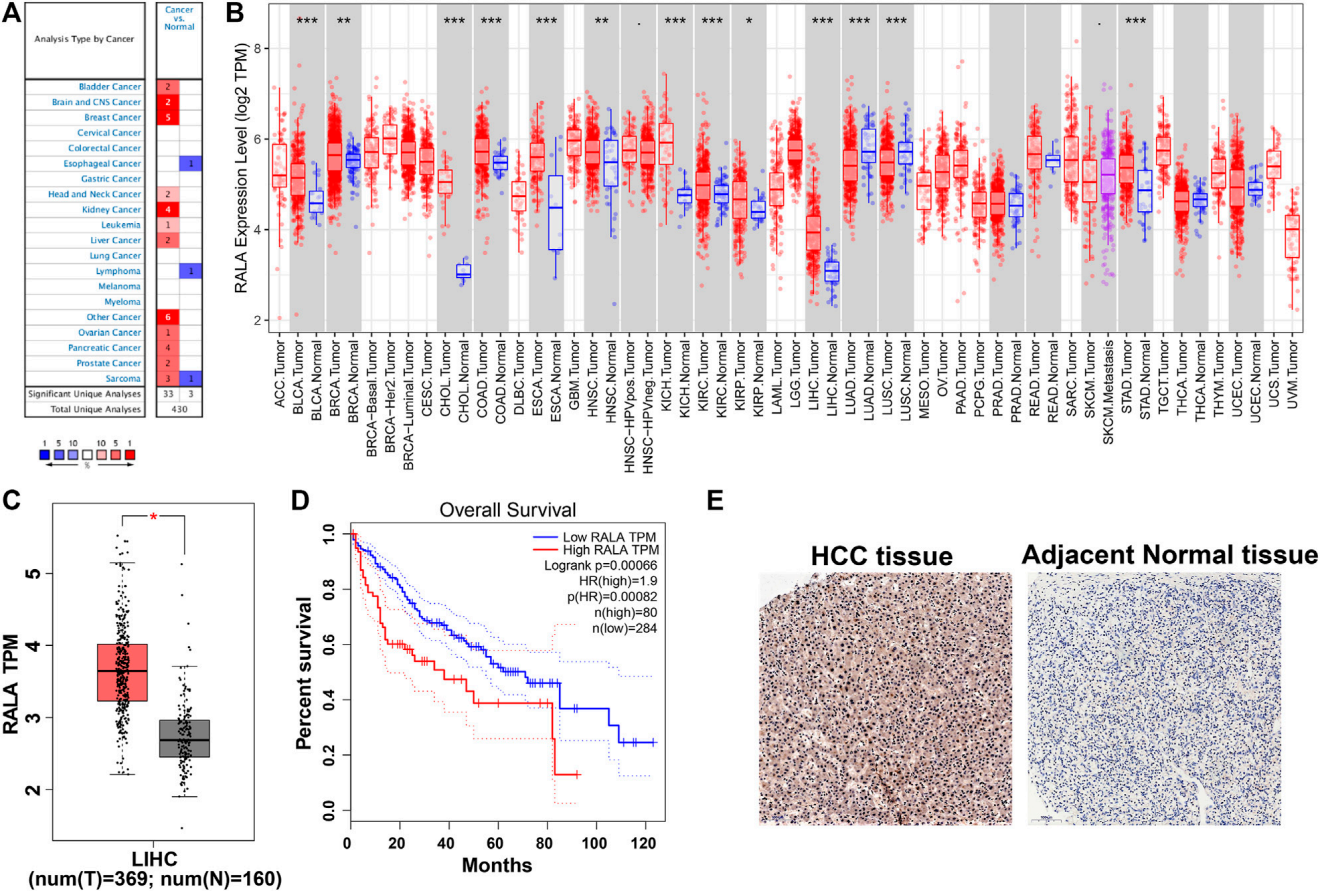
**(A)** Analysis of the differential RalA expression of various cancer types in the Oncomine database; **(B)** Differential expression profiles of various cancers and RalA in the TCGA database; **(C)** Differential expression of RalA protein in HCC and normal tissues; **(D)** Kalplan–Meier curves of RalA on the overall survival rate of HCC patients in the TCGA database; **(E)** Protein expression levels of RalA in HCC and adjacent normal tissues detected by IHC, scale bar: 100 μm.

We observed that RalA mRNA levels were upregulated in the Oncomine database cancer subtype panels (Figure 2A). Results suggested that RalA was overexpressed in different cancer subtypes, including bladder cancer, breast cancer, liver cancer, and prostate cancer (He et al., 2019; Pan et al., 2018). Next, we assessed RalA mRNA expression patterns in the TCGA database. RalA was upregulated in ten TCGA subtype cohorts (fold change >2.0, *p* < 0.01) and was downregulated in two lung cancer cohorts (Figure 2B). The analysis of 369 HCC-afflicted tissues and 160 adjacent normal liver tissue samples from data in the TCGA and GTEx databases demonstrated that RalA mRNA expression in HCC-afflicted tissues was 2.67-fold higher than in adjacently sampled normal ones (Figure 2C) (Wang et al., 2018; Hao et al., 2019; Wang et al., 2019). Furthermore, the RalA mRNA levels were a significant marker to predict HCC-afflicted patients’ prognosis (Figure 2D). IHC-derived images of RalA proteins indicated that they were overexpressed in HCC vs. normal liver tissues (Figure 2E). Based on these results, we hypothesized that the upregulated RalA may be an attractive target for use in HCC-related therapies.

**FIGURE 3:**
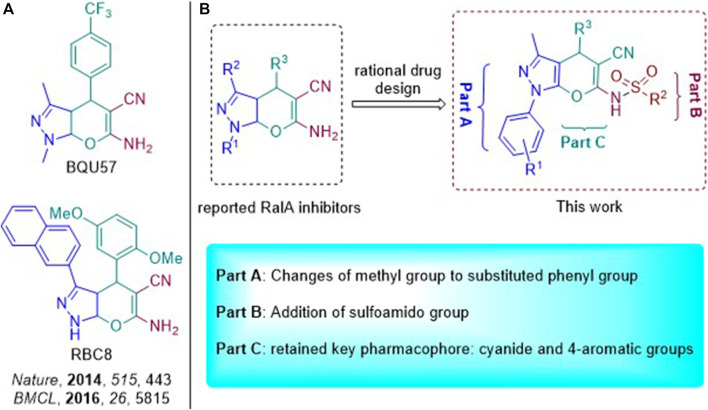
**(A)** Pyran-fused pyrazole derivatives reported to inhibit RalA; **(B)** Design strategy of novel RalA inhibitors.

BQU57 and RBC8 as RalA inhibitors were recently reported (Figure 3A) and suggested that they were characterized by a pyran-fused pyrazole scaffold. This type of a scaffold constitutes a core structure that can bind to the RalA-GDP allosteric site, with 2-amino and 3-cyanide groups on the pyran moiety, being key pharmacophores. Pyrano[2,3-c]-pyrazole–based RalA inhibitors’ effects on cancer cell proliferation have also been reported. However, we aimed to further develop efficient small-molecule allosteric RalA inhibitors based on these scaffold dynamics. Based on the necessary 5-nitrile-6-amino-pyrano[2, 3-c] pyrazole scaffold, variations in functional groups (R^1^ and R^2^) were introduced into the core structure, enriching the drug-like skeleton diversity (Figure 3B). The resulting compounds may, thus, have great potential to serve as foundations for exploring their uses as novel and efficient RalA inhibitors.

**SCHEME 1:**
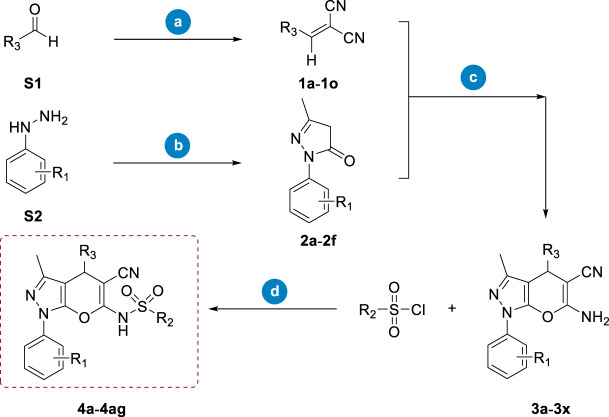
Synthetic route of compounds **4a**–**4ag**. Reagents and conditions: **(A)**. Malononitrile, acetic acid, and CH_3_COONa, overnight; **(B)**. Ethyl acetoacetate, acetic acid, and reflux, 4 h; **(C)**. Morpholine and methanol, 40°C, 30 min; **(D)**. DMAP and DCM, 35°C, 12 h.

Considering both feasible synthetic routes and the chemical diversity of novel RalA inhibitors, we employed the synthetic route depicted in Scheme 1. Briefly, the starting compound (**1a–1o**) was a cyano-olefin formed by aldehyde and Malononitrile reactions via Knoevenagel condensation in the presence of acetic acid and sodium acetate. The corresponding pyrazolone (**2a–2f**) was synthesized from ethyl acetoacetate and phenylhydrazine by heating and refluxing acetic acid constituents for 4 h. The **1a–1o** and **2a–2f** compounds were further used as raw materials. Then, morpholine was added into methanol (solvent) followed by heating and stirring for 30 min to form pyrano [2,3-c]pyrazole intermediates (**3a–3x**). Finally, intermediates **3a–3x** reacted with a panel of sulfonyl chloride in the presence of DMAP using dichloromethane (DCM) as a solvent to obtain the target compound 4a–4ag (Scheme 1).

The structures of the synthesized target compounds (**4a**–**4ag**) were characterized by their HR-MS, ^1^H-NMR, and ^13^C-NMR spectra. Their RalA/B inhibitory activities at 1.0 μM and cellular proliferation data for HCC cell lines are shown in Table 1. When different pyrano [2,3-c]-pyrazole ring–type bioactivities were compared, the remaining RalA/B kinase activity was relatively high for pyran ring substituents with moderate steric hindrance (e.g. benzene rings or halogen-substituted phenyl groups). However, the corresponding HepG2 and Huh7 IC_50_ values were higher than 10 μM. When the 4-substitution of the pyran ring (R^3^ substitution) was a 4-trifluoromethyl phenyl or heteroaromatic ring, better activities were observed. Our findings also indicated that the hydrogen atom in the R^1^ group was preferred using **4s–4ag**. Any changes at this site, such as halogen, methyl, or methoxyl substitutions led to a loss of RalA/B and HCC cell proliferation inhibitory activities. We found that the p-methoxybenzenesulfonyl group was the R^2^ -substituted fragment from compound **4n–4ab** with the highest inhibitory activities against RalA/B and HCC cells. 1.0 μM of **4p** was added before incubation, and only 13 and 22% of RalA and RalB kinase activities were detected, respectively. After compound **4p’s** application, its IC_50_ values for HepG2 and Huh7 cells were 2.28 ± 0.23 and 4.31 ± 0.39 μM, respectively.

**TABLE 1:** Remaining kinase activities (%) after 1 μM compounds **4a**–**4ag** incubation on RalA and RalB and cell proliferation inhibition.
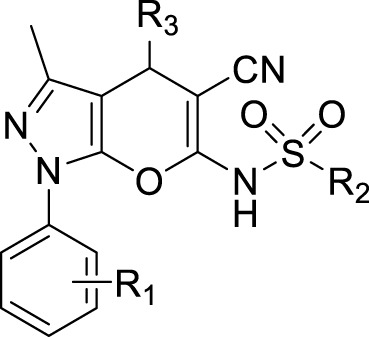

No	R^1^	R^2^	R^3^	% control^a^ 1 μM		IC_50_ (μM)	
				RalA	RALB	HepG2	Huh7
**4a**	H	4-MeC_6_H_4_	Ph	74	85	>10	>10
**4b**	H	4-MeC_6_H_4_	4-MeC_6_H_4_	75	105	>10	>10
**4c**	H	4-MeC_6_H_4_	3-ClC_6_H_4_	40	69	7.28 ± 0.64	>10
**4d**	H	4-MeC_6_H_4_	4-BrC_6_H_4_	77	89	>10	>10
**4e**	H	4-MeC_6_H_4_	4-FC_6_H_4_	74	76	>10	>10
**4f**	H	4-MeC_6_H_4_	3-BrC_6_H_4_	47	71	8.83 ± 0.75	>10
**4g**	H	4-MeC_6_H_4_	3,4-diCl C_6_H_3_	66	58	>10	>10
**4h**	H	4-MeC_6_H_4_	2-F-4-Br C_6_H_3_	70	83	>10	>10
**4i**	H	4-MeC_6_H_4_	4-MeOC_6_H_4_	69	60	>10	>10
**4j**	H	4-MeC_6_H_4_	2-furyl	57	70	9.57 ± 1.05	9.94 ± 1.23
**4k**	H	4-MeC_6_H_4_	3-pyridyl	32	38	5.63 ± 0.58	6.01 ± 0.57
**4l**	H	4-MeC_6_H_4_	4-pyridyl	40	52	9.2 ± 0.89	9.15 ± 1.04
**4m**	H	4-MeC_6_H_4_	3-thienyl	45	58	7.2 ± 0.65	>10
**4n**	H	4-MeC_6_H_4_	4-CF_3_C_6_H_4_	46	59	8.46 ± 0.92	9.58 ± 0.92
**4o**	H	2-thienyl	4-CF_3_C_6_H_4_	36	47	5.32 ± 0.61	>10
**4p**	H	4-MeOC_6_H_4_	4-CF_3_C_6_H_4_	13	22	2.28 ± 0.23	4.31 ± 0.39
**4q**	H	4-CF_3_C_6_H_4_	4-CF_3_C_6_H_4_	37	39	8.65 ± 0.97	8.42 ± 0.94
**4r**	H	2,4-diFC_6_H_3_	4-CF_3_C_6_H_4_	30	43	7.56 ± 0.92	7.39 ± 0.85
**4s**	2-Cl	4-MeC_6_H_4_	2-furyl	48	72	9.89 ± 0.81	>10
**4t**	2-Cl	4-MeC_6_H_4_	3-pyridyl	46	65	8.28 ± 0.89	>10
**4u**	2-Cl	4-MeC_6_H_4_	4-pyridyl	44	50	8.71 ± 0.74	>10
**4v**	2-Cl	4-MeC_6_H_4_	3-thienyl	56	78	>10	>10
**4w**	2-Cl	4-MeC_6_H_4_	3-MeOC_6_H_4_	51	69	8.87 ± 0.95	>10
**4x**	2-Cl	Ph	3-MeOC_6_H_4_	59	58	>10	8.93 ± 1.02
**4y**	2-Cl	4-MeOC_6_H_4_	3-MeOC_6_H_4_	54	73	>10	>10
**4z**	2-Cl	2-NO_2_C_6_H_4_	3-MeOC_6_H_4_	53	60	>10	>10
**4aa**	2-Cl	3-NO_2_C_6_H_4_	3-MeOC_6_H_4_	72	73	>10	>10
**4ab**	2-Cl	4-NO_2_C_6_H_4_	3-MeOC_6_H_4_	60	65	>10	>10
**4ac**	4-Cl	4-MeC_6_H_4_	Ph	67	68	>10	>10
**4ad**	2-Cl	4-MeC_6_H_4_	Ph	52	64	>10	>10
**4ae**	4-Me	4-MeC_6_H_4_	Ph	79	78	>10	>10
**4af**	2-OMe	4-MeC_6_H_4_	Ph	73	78	>10	>10
**4ag**	4-F	4-MeC_6_H_4_	Ph	66	75	>10	>10

a % control = kinase activity remained.

In Table 2 we list the IC_50_ values for compounds **4k–4r** for RalA/B and HepG2, Hep3B, Huh-7, and SMMC-7721 HCC cell proliferation. Compound **4p** demonstrated the best RalA and RalB inhibitory activities with IC_50_ values of 0.22 and 0.41 μM, respectively. All eight compounds displayed good to moderately good inhibitory effects on RalA/B and HepG2 cell proliferation. Compounds **4l**, **4m**, and **4p** exhibited potent cellular proliferation inhibitory capacities on the HCC cell lines. In the current study, HepG2 was the most sensitive cell line with IC_50_ values of 6.01, 9.15, and 2.28 μM for compounds **4l**, **4m**, and **4p,** respectively. Therefore, **4p** was selected as the best compound to increase the understanding of its RalA binding and its potential molecular mechanisms against HCC in subsequent experiments.

**TABLE 2:** The IC_50_ values (μM) of compounds **4k**–**4r** on RalA/B and HCC cell lines.

No	Kinase activities (IC_50,_ μM)^a^		Anti-proliferative activities (IC_50_, μM)^b^			
	RalA	RalB	HepG2	Huh-7	Hep3B	SMMC-7721
**4k**	0.75 ± 0.09	1.09 ± 0.13	5.63 ± 0.58	6.01 ± 0.57	>10	>10
**4l**	1.24 ± 0.15	2.64 ± 0.30	9.2 ± 0.89	9.15 ± 1.04	4.39 ± 0.57	5.94 ± 0.77
**4m**	1.70 ± 0.22	3.86 ± 0.39	7.2 ± 0.65	>10	9.84 ± 1.37	9.60 ± 1.05
**4n**	1.81 ± 0.19	4.11 ± 0.45	8.46 ± 0.92	9.58 ± 0.92	>10	>10
**4o**	0.96 ± 0.12	1.93 ± 0.26	5.32 ± 0.61	>10	7.69 ± 0.84	>10
**4p**	0.22 ± 0.04	0.41 ± 0.07	2.28 ± 0.23	4.31 ± 0.39	2.71 ± 0.27	5.38 ± 0.37
**4q**	1.02 ± 0.09	1.16 ± 0.19	8.65 ± 0.97	8.42 ± 0.94	7.76 ± 1.00	>10
**4r**	0.66 ± 0.08	1.49 ± 0.22	7.56 ± 0.92	7.39 ± 0.85	8.30 ± 0.66	>10

a IC_50_ values for enzymatic inhibition of RalA and RalB; Data displayed are the average of at least three independent replicates ±standard deviation.

b IC_50_ = compound concentration required to inhibit tumor cell proliferation by 50%; Data displayed are the average of at least three independent replicates ±standard deviation.

**FIGURE 4:**
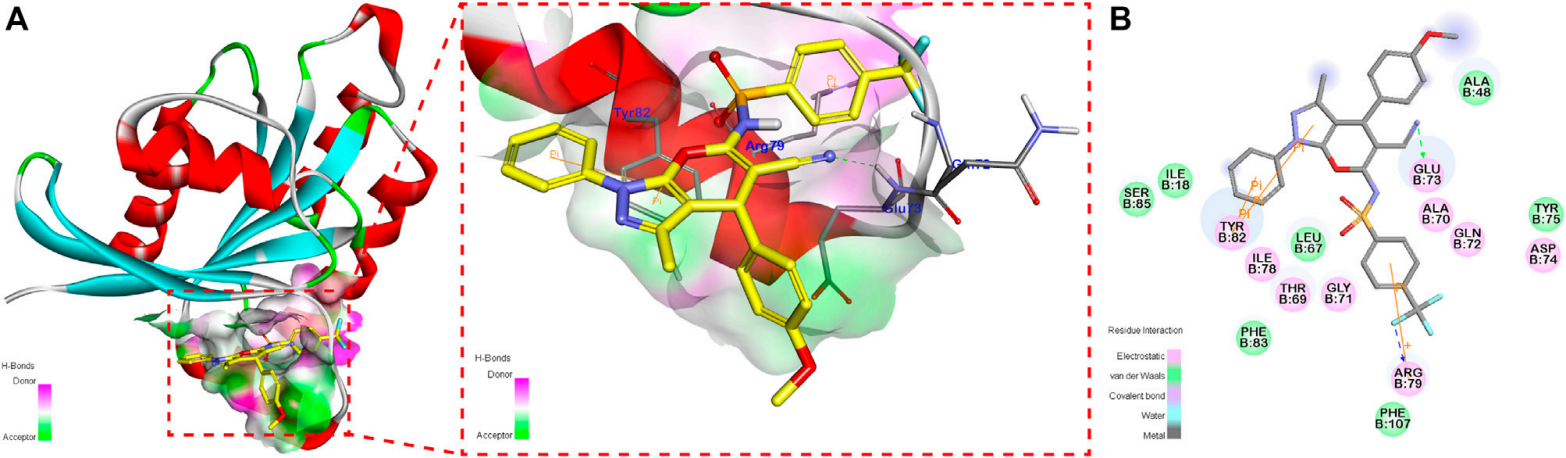
3D contour **(A)** and 2D contour **(B)** of the binding mode of compound 4p in at the RalA-GDP allosteric site.

The 3D contours of **4p** bounded to RalA allosteric sites are shown in Figure 4. We observed that the cyano group of **4p** formed a hydrogen bond with the RalA Glu73 residue. The benzene ring at the R^1^ position and the pyrazole ring formed a π–π conjugation system with RalA Tyr82 residues, which might strengthen the stable binding. A cation–π interaction between **4p** p-trifluoromethyl benzenesulfonyl fragment and RalA Arg79 residue was also detected.

**FIGURE 5:**
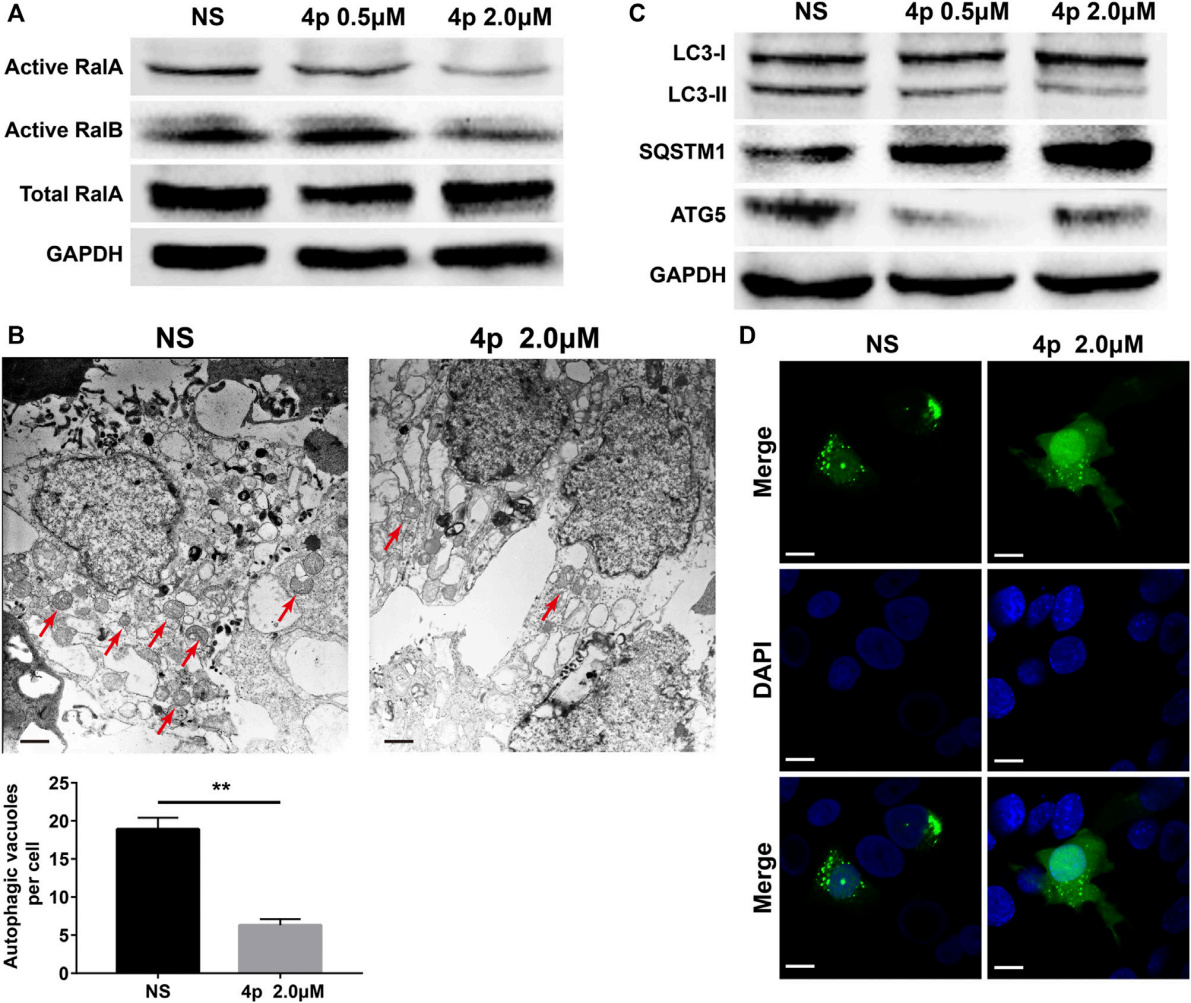
**(A)** Selective inhibition of **4p** on RalA was observed by the GST pulldown test; **(B)** Transmission electron micrograph of HepG2 cells treated with **4p**, scale bar: 500 nm; **(C)** Western blotting was used to detect the change of the LC3-II/I ratio; **(D)** Formation of autophagosome in GFP-LC3–transfected HepG2 cells induced by 2.0 μM **4p**, scale bar: 6 μm.

GST pulldown assays were performed to assess **4p** RalA/B inhibitory capacities. 2.0 μM of compound **4p** significantly suppressed activated RalA and RalB protein expression levels. Only activated RalA was suppressed after incubation with 0.5 μM of compound **4p** (Figure 5A). Additionally, total RalA expression was unchanged, which suggested that **4p** potentially interfered with RalBP1’s binding to RalA/B.

**FIGURE 6:**
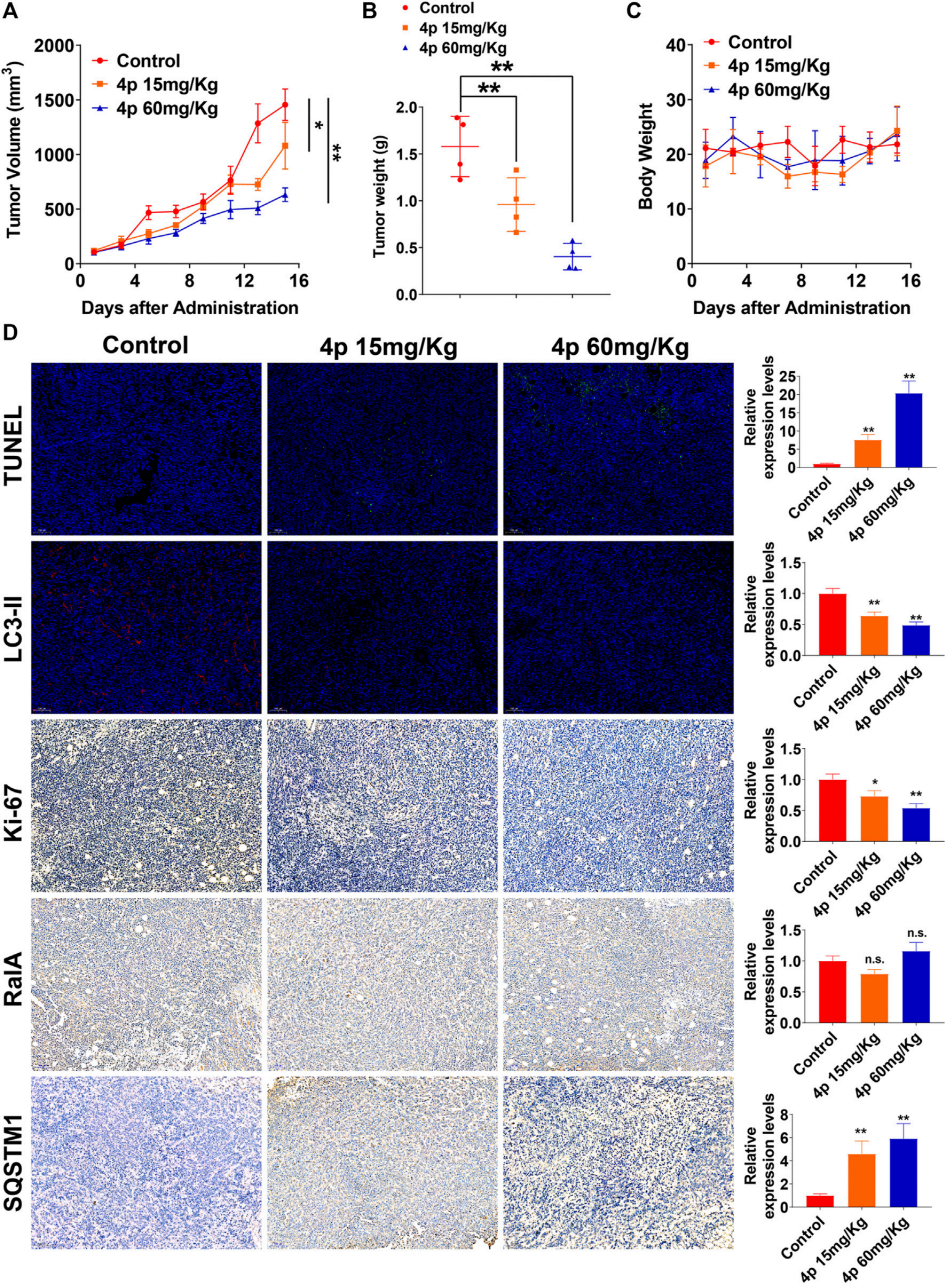
Compound **4p** inhibited tumor growth in a HepG2 xenograft model. **(A)** Tumor volume changes were determined every 2 days after drug administration; **(B)** Tumor weight in each mouse in **(A, C)** The body weight of mice in each group; **(D)** Immunofluorescence and IHC images and analysis of tissue sections stained by TUNEL, Ki-67, RalA, LC3-II, and SQSTM1 in each group, scale bar: 100 μm.

Several reports have characterized RalA/B potential regulatory roles in autophagy (Zhang et al., 2019; Pang et al., 2020; Zhang et al., 2020). Considering the dual effect of HCC autophagy, we assessed changes in HepG2 cell autophagy levels after the addition of 0.5 or 2.0 μM of **4p**, before incubation. Based on HepG2 cells’ TEM images with or without **4p** (Figure 5B), autophagy vacuoles per cell decreased in the **4p-**treated group (*p* < 0.01). After **4p** treatments, LC3-II and ATG5 protein levels declined and SQSTM1 increased. These results suggested that **4p** suppressed the autophagy flux in HepG2 cells (Figure 5C). In GFP-LC3–transfected HepG2 cells, cytoplasm LC3 fluorescent puncta numbers also declined after **4p** treatments. These results indicated that the **4p** addition suppressed autophagy in HepG2 cells. The *in vivo* antitumor capacities of **4p** were determined on a HepG2 subcutaneous xenograft model. The **4p** intraperitoneal injections were set to 15 mg/kg and 60 mg/kg, according to *in vitro* results. We observed that at a high dosage, the treatment group presented superior tumor growth inhibition (TGI) (63%), compared to the low-dosage group (39%) (Figure 6A). Moreover, mean tumor weights in both high- and low-dosage groups notably declined compared to the control (*p* < 0.01) without significant changes in body weights (Figures 6B,C). The potential **4p** molecular mechanisms were further validated using immunofluorescence and IHC analyses of tissue sections stained by antibodies against TUNEL, Ki-67, RalA, LC3-II, and SQSTM1. In the **4p** therapy groups, we found that a high **4p** dosage group induced declined suppression levels of Ki-67 positive cells (*p* < 0.01), induced increased TUNEL and SQSTM1 levels, and decreased LC3-II expression (*p* < 0.01). No clear changes in total RalA levels were detected (Figure 6D). Therefore, the *in vivo* results were consistent with *in vitro* experiments.

## Conclusion

Overall, we reported design, synthesis, and biologically based evaluation details of 6-sulfonamide-pyrano[2,3-c]-pyrazole as a novel RalA inhibitor against HCC, both *in vitro* and *in vivo*. Compound **4p** was the most effective compound tested. It significantly inhibited cell proliferation and suppressed autophagy in HepG2 cells. These results can be a solid base for further exploration and development of novel RalA inhibitor candidates.

## Experimental

### General Information

Reactions were monitored using TLC purchased from a commercial supplier. Melting points were determined using a Reichert Thermovar apparatus. Proton nuclear magnetic resonance (^1^H NMR, 400 MHz) spectra and carbon nuclear magnetic resonance (^13^C NMR, 100 MHz) spectra were recorded on a Bruker Avance III NMR spectrometer. TMS (tetramethylsilane) was used as the internal standard and chloroform-d or DMSO-d_6_ was used to dissolve samples. Chemical shifts (d) were marked in ppm. ESI-HRMS spectrum data were collected using a Waters TOF-MS instrument (Waters, Milford, MA, United States). Nitrogen was used as the nebulizing gas, desolvation gas, and cone curtain gas. Chemicals received from commercial sources were used without further purification. Column chromatography was performed on silica gel (400–500 mesh) eluting with ethyl acetate and petroleum ether. TLC was performed on glass-backed silica plates. UV light and I_2_ were used to visualize products.

#### Reagents, Antibodies, and Cell Cultures

The antibodies recognizing LC3, p62/SQSTM1, RalA, RalB, and GAPDH were purchased from Proteintech (Wuhan, China). HCC cell lines, including HepG2, Hep3B, Huh-7, and SMMC-7721, were obtained from the Chinese Center for Type Culture Collection (Wuhan, China) and cultured in DMEM (Dulbecco’s modified Eagle’s medium) with 10% FBS (fetal bovine serum) and streptomycin. Cytotoxicity assays were performed by using the MTT method as previously described.

#### Biochemical and *In Vitro* Bioassays

The *in vitro* RalA/B bioactivity assays were performed following methods outlined in previous reports. (Yan et al., 2016; Zhang et al., 2018; Walsh et al., 2019; Bum-Erdene et al., 2020). In brief, the lysates of the test compound or vehicle-treated cells were cleared using cold centrifugation, and supernatants were collected. An RalBP1 solution was added to 96-well plates and incubated for 1–2 h, and then the plates were washed thrice using ELISA buffer. Next, cellular lysates were added, and samples were incubated overnight at 4°C. Then, ice-cold mouse anti-FLAG antibody was added, and samples were incubated for one more hour. After subsequent washing with ELISA buffer thrice, the HRP-conjugated anti-mouse antibody was added, and samples were incubated for another hour. Then, the HRP substrate was added. Antigen–antibody reactions were quenched by the addition of sulfuric acid (2 mol/L). Remaining RalA/B was detected by the OD450 values which were collected by using a plate reader. Cell proliferation assays, colony formation assays, apoptosis assays, GST pulldown, and Western blotting were used following methods outlined in our previous reports. (Zhou et al., 2015; Zhang et al., 2016; Yang et al., 2017; He et al., 2019). Autophagy levels and protein immunoblotting were used following the methods mentioned in our previous reports (Pan et al., 2018; Wang et al., 2018; Hao et al., 2019; Wang et al., 2019; Peng et al., 2020). Detailed experimental procedures are described in supporting materials.

#### Xenograft Models and *In Vivo* Evaluation

Female Balb/c nude mice aged 6–8 weeks were purchased from Beijing Huafukang Co. Ltd. (Beijing, China). Animal-based experiments complied with the guidelines of the Animal Ethics Committee (Sichuan University), and all aspects were approved by the Animal Ethics Committee of West China Hospital of Sichuan University (China). In brief, HepG2 cells were injected subcutaneously in the dorsal flank of Balb/c nude mice (5*10^5^ cells per mice). Tumor volumes were recorded every 2 days post cancer cell injection. After the therapeutic endpoint, all mice were euthanized, and tumor tissues were stripped, fixed in formalin, embedded in paraffin, and sectioned. The IHC and immunofluorescent staining methods were performed following methods as per our previous research, (Zhang et al., 2019; Pan et al., 2020; Pang et al., 2020; Zhang et al., 2020), and detailed experimental procedures are also described in supporting materials.

#### General Procedure of Method for the Synthesis of 4a-4ag

To achieve the desired compound 2-benzylidenemalononitrile (**1a**), the following steps were completed. First, a mixture of benzaldehyde (10.0 mmol, 1.06 g), malononitrile (10.0 mmol, 0.66 g), sodium acetate (5.0 mmol, 0.41 g), and acetic acid (10 ml) was stirred overnight at room temperature. TLC was used to monitor whether or not the reaction was complete. When the basic reaction of raw materials was complete, ethyl acetate (30 ml) was added after the stirring was stopped, and glacial acetic acid was neutralized by NaHCO_3_ until pH values up to 7.0 were observed. The organic layer was separated and concentrated, and the crude product was purified using silica gel column chromatography using a mixed solvent of petroleum ether/ethylacetate (4:1) to give compound **1a**.

To achieve the desired compound 2-benzylidene-malononitrile (**2b**), the following steps were completed. First, a mixture of 4-chlorophenylhydrazine salt (20 mmol, 3.6 g) and ethyl acetoacetate (30 mmol, 3.8 ml) was dissolved in 30 ml of glacial acetic acid and refluxed for 4 h. TLC was used to monitor whether or the reaction was complete. When the basic reaction of raw materials was complete, ethyl acetate (30 ml) was added on when the stirring was stopped. Then, glacial acetic acid was neutralized by NaHCO_3_ to a pH value = 7. The organic layer was separated and concentrated, and the crude product was purified using silica gel column chromatography using a mixed solvent of petroleum ether/ethylacetate (6:1) to give compound **2b**.

6-Amino-3-methyl-1,4-diphenyl-1,4-dihydropyrano[2,3c]-pyrazole-5-carbonitrile (3a):A mixture of **1a** (5 mmol, 0.77 g), **2a** (5 mmol, 1.04 g) and morpholine (2.5 mmol, 0.22 g) in 10 ml methanol was stirred at 40°C for 30 min. The reaction mixture was filtered and washed with a small amount of ice-cold methanol to give the crude product, and then the crude product was purified using silica gel column chromatography using a mixed solvent of petroleum ether/ethylacetate (5:1) to give the compound **3a**. Yield: 90%, white power solid, m. p. 170–172°C. ^1^H NMR (400 MHz, CDCl_3_) δ 7.65 (dt, *J* = 7.3, 1.4 Hz, 2H), 7.46 (tt, *J* = 7.5, 1.6 Hz, 2H), 7.39–7.30 (m, 3H), 7.30–7.24 (m, 3H), 4.67 (s, 2H), 4.66 (s, 1H), and 1.89 (s, 3H) ppm.

6-Amino-3-methyl-1-phenyl-4-(p-tolyl)-1,4-dihydropyrano-[2,3-c]-pyrazole-5-carbonitrile (3b): Synthesized with **1b** and **2a** according to the general procedure of **3a**. Yield: 91%, white power solid, m. p. 169–170°C. ^1^H NMR (400 MHz, CDCl_3_) δ 7.68–7.63 (m, 2H), 7.45 (td, *J* = 8.3, 1.7 Hz, 2H), 7.30 (tt, *J* = 7.4, 1.6 Hz, 1H), 7.16–7.11 (m, 4H), 4.66 (s, 2H), 4.62 (s, 1H), 2.34 (s, 3H), 1.90 (s, 3H) ppm.

6-Amino-4-(3-chlorophenyl)-3-methyl-1-phenyl-1,4-dihydropyrano[2,3-c]-pyrazole-5-carbonitrile (3c): Synthesized with **1c** and **2a** according to the general procedure of **3a**. Yield: 88%, white power solid, m. p. 155–157°C. ^1^H NMR (400 MHz, DMSO-d_6_) δ 7.80–7.78 (m, 2H), 7.49(tt, *J* = 8.6, 1.9 Hz, 2H), 7.41–7.37 (m, 3H), 7.34–7.30 (m, 3H)), 7.27–7.24 (m, 3H), 4.75 (s, 1H), 1.81 (s, 3H) ppm.

6-Amino-4-(4-bromophenyl)-3-methyl-1-phenyl-1,4-dihydropyrano[2,3-c]-pyrazole-5-carbonitrile (3d): Synthesized with **1d** and **2a** according to the general procedure of **3a**. Yield: 90%, white power solid, m. p. 181–183°C. ^1^H NMR (400 MHz, CDCl_3_) δ 7.67–7.62 (m, 2H), 7.51–7.42 (m, 4H), 7.32 (t, *J* = 7.4 Hz, 1H), 7.14 (dt, *J* = 8.4, 2.6 Hz, 2H), 4.72 (s, 2H), 4.64 (s, 1H), 1.89 (s, 3H) ppm.

6-Amino-4-(4-fluorophenyl)-3-methyl-1-phenyl-1,4-dihydropyrano[2,3-c]-pyrazole-5-carbonitrile (3e): Synthesized with **1e** and **2a** according to the general procedure of **3a**. Yield: 83%, yellow power solid, m. p. 173–176°C. ^1^H NMR (400 MHz, DMSO-d_6_) δ 7.79 (d, *J* = 8.0 Hz, 2H), 7.49 (t, *J* = 7.8 Hz, 2H), 7.34–7.29 (m, 3H), 7.22–7.15 (m, 4H), 4.73 (s, 1H), 1.79 (s, 3H) ppm.

6-Amino-4-(3-bromophenyl)-3-methyl-1-phenyl-1,4-dihydropyrano[2,3-c]-pyrazole-5-carbonitrile (3f): Synthesized with **1f** and **2a** according to the general procedure of **3a**. Yield: 87%, white power solid, m. p. 161–164°C. ^1^H NMR (400 MHz, CDCl_3_) δ 7.66–7.63 (m, 2H), 7.47 (tt, *J* = 6.7, 2.0 Hz, 2H), 7.42 (dt, *J* = 6.5, 2.1 Hz, 1H), 7.36–7.30 (m, 2H), 7.23–7.22 (m, 2H), 4.75 (s, 2H), 4.63 (s, 1H), 1.91 (s, 3H) ppm.

6-Amino-4-(3,4-dichlorophenyl)-3-methyl-1-phenyl-1,4-dihydropyrano[2,3-c]-pyrazole-5-carbonitrile (3g): Synthesized with **1g** and **2a** according to the general procedure of **3a**. Yield: 85%, white power solid, m. p. 198–200°C. ^1^H NMR (400 MHz, CDCl_3_) δ 7.66–7.63 (m, 2H), 7.49–7.42 (m, 3H), 7.34 (dt, *J* = 7.4, 1.1 Hz, 1H), 7.32 (d, *J* = 2.1 Hz, 1H), 7.13 (dd, *J* = 8.2, 2.1 Hz, 1H), 4.77 (s, 2H), 4.64 (s, 1H), 1.92 (s, 3H) ppm.

6-Amino-4-(4-bromo-2-fluorophenyl)-3-methyl-1-phenyl-1,4-dihydropyrano[2,3-c]-pyrazole-5-carbonitrile (3h): Synthesized with **1h** and **2a** according to the general procedure of **3a**. Yield: 85%, white power solid, m. p. 200–203°C. ^1^H NMR (400 MHz, DMSO-d_6_) δ 7.81–7.75 (m, 2H), 7.56 (dd, *J* = 10.1, 2.0 Hz, 1H), 7.53–7.47 (m, 2H), 7.42 (dd, *J* = 8.3, 1.9 Hz, 1H), 7.37–7.26 (m, 4H), 4.97 (s, 1H), 1.83 (s, 3H) ppm.

6-Amino-4-(4-methoxyphenyl)-3-methyl-1-phenyl-1,4-dihydropyrano[2,3-c]-pyrazole-5-carbonitrile (3i): Synthesized with **1i** and **2a** according to the general procedure of **3a**. Yield: 90%, white power solid, m. p. 176–178°C. ^1^H NMR (400 MHz, DMSO-d_6_) δ 7.82–7.76 (m, 2H), 7.49 (tt, *J* = 8.6, 2.0 Hz, 2H), 7.31 (tt, *J* = 7.4, 1.1 Hz, 2H), 7.18–7.15 (m, 4H), 6.90 (dt, *J* = 8.6, 2.0 Hz, 1H), 4.63 (s, 1H), 3.75 (s, 3H), 1.79 (s, 3H) ppm.

6-Amino-4-(furan-2-yl)-3-methyl-1-phenyl-1,4-dihydropyrano[2,3-c]-pyrazole-5-carbonitrile (3j): Synthesized with **1j** and **2a** according to the general procedure of **3a**. Yield: 60%, yellow power solid, m. p. 225.1–225.5°C. ^1^H NMR (400 MHz, CDCl_3_) δ 7.62 (d, *J* = 7.8 Hz, 2H), 7.45 (t, *J* = 7.9 Hz, 2H), 7.37 (d, *J* = 0.9 Hz, 1H), 7.31 (t, *J* = 7.4 Hz, 1H), 6.34 (dd, *J* = 3.0, 1.9 Hz, 1H), 6.23 (d, *J* = 3.2 Hz, 1H), 4.83 (s, 1H), 4.75 (s, 2H), 2.09 (s, 3H) ppm.

6-Amino-3-methyl-1-phenyl-4-(pyridin-3-yl)-1,4-dihydropyrano[2,3-c]-pyrazole-5-carbonitrile (3k): Synthesized with **1k** and **2a** according to the general procedure of **3a**. Yield: 80%, white power solid, m. p. 188.7–189.1°C. ^1^H NMR (400 MHz, DMSO-d_6_) δ 8.55 (d, *J* = 2.0 Hz, 1H), 8.49 (dd, *J* = 4.7, 1.5 Hz, 1H), 7.79 (d, *J* = 7.7 Hz, 2H), 7.66 (dt, *J* = 7.9, 1.8 Hz, 1H), 7.50 (t, *J* = 8.0 Hz, 2H), 7.38 (dd, *J* = 7.8, 4.8 Hz, 1H), 7.33 (t, *J* = 7.4 Hz, 1H), 7.29 (s, *J* = 7.0 Hz, 2H), 4.79 (s, 1H), 1.79 (s, 3H) ppm.

6-Amino-3-methyl-1-phenyl-4-(pyridin-4-yl)-1,4-dihydropyrano[2,3-c]-pyrazole-5-carbonitrile (3l): Synthesized with **1l** and **2a** according to the general procedure of **3a**. Yield: 80%, white power solid, m. p. 192.2–192.6°C. ^1^H NMR (400 MHz, DMSO-d_6_) δ 8.59 (dd, *J* = 4.7, 1.3 Hz, 2H), 7.83–7.77 (m, 2H), 7.52–7.48 (m, 2H), 7.40–7.31 (m, 5H), 4.79 (s, 1H), 1.82 (s, 3H) ppm.

6-Amino-3-methyl-1-phenyl-4-(thiophen-3-yl)-1,4-dihydropyrano[2,3-c]-pyrazole-5-carbonitrile (3m): Synthesized with **1m** and **2a** according to the general procedure of **3a**. Yield: 85%, yellow power solid, m. p. 178.2–178.6°C. ^1^H NMR (400 MHz, DMSO-d_6_) δ 7.81–7.75 (m, 2H), 7.53–7.45 (m, 3H), 7.37 (dd, *J* = 2.9, 1.2 Hz, 1H), 7.31 (t, *J* = 7.4 Hz, 1H), 7.18 (s, 2H), 6.95 (dd, *J* = 5.0, 1.2 Hz, 1H), 4.82 (s, 1H), 1.86 (s, 3H) ppm.

6-Amino-3-methyl-1-phenyl-4-(4-(trifluoromethyl) phenyl)-1,4-dihydropyrano[2,3-c]-pyrazole-5-carbonitrile (3n): Synthesized with **1n** and **2a** according to the general procedure of **3a**. Yield: 85%, white power solid, m. p. 162.9–163.5°C. ^1^H NMR (400 MHz, DMSO-d_6_) δ 7.79 (d, *J* = 7.8 Hz, 2H), 7.73 (d, *J* = 8.1 Hz, 2H), 7.50 (t, *J* = 8.3 Hz, 4H), 7.33 (t, *J* = 7.6 Hz, 3H), 4.85 (s, 1H), 1.79 (s, 3H) ppm.

6-Amino-1-(2-chlorophenyl)-4-(furan-2-yl)-3-methyl-1,4-dihydropyrano[2,3-c]-pyrazole-5-carbonitrile (3o): Synthesized with **1j** and **2b** according to the general procedure of **3a**. Yield: 62%, yellow power solid, m. p. 86.8–87.2°C. ^1^H NMR (400 MHz, DMSO-d_6_) δ 7.70 (dd, *J* = 7.8, 1.5 Hz, 1H), 7.59 (td, *J* = 7.5, 2.2 Hz, 2H), 7.57–7.48 (m, 2H), 7.12 (s, 2H), 6.42 (dd, *J* = 3.1, 1.9 Hz, 1H), 6.27 (d, *J* = 3.1 Hz, 1H), 4.90 (s, 1H), 1.96 (s, 3H) ppm.

6-Amino-1-(2-chlorophenyl)-3-methyl-4-(pyridin-3-yl)-1,4-dihydropyrano[2,3-c]-pyrazole-5-carbonitrile (3p): Synthesized with **1k** and **2b** according to the general procedure of **3a**. Yield: 63%, white power solid, m. p. 165.9–166.4°C. ^1^H NMR (400 MHz, DMSO-d_6_) δ 8.56–8.47 (m, 2H), 7.71 (dd, *J* = 7.7, 1.6 Hz, 1H), 7.68–7.61 (m, 2H), 7.59–7.51 (m, 2H), 7.41 (dd, *J* = 7.8, 4.8 Hz, 1H), 7.15 (s, 2H), 4.80 (s, 1H), 1.78 (s, 3H) ppm.

6-Amino-1-(2-chlorophenyl)-3-methyl-4-(pyridin-4-yl)-1,4-dihydropyrano[2,3-c]-pyrazole-5-carbonitrile (3q): Synthesized with **1l** and **2b** according to the general procedure of **3a**. Yield: 61%, white power solid, m. p. 200.1–200.7°C. ^1^H NMR (400 MHz, DMSO-d_6_) δ 8.57 (dd, *J* = 4.4, 1.5 Hz, 2H), 7.71 (dd, *J* = 7.8, 1.6 Hz, 1H), 7.66 (dd, *J* = 7.3, 2.2 Hz, 1H), 7.59–7.51 (m, 2H), 7.28 (dd, *J* = 4.5, 1.5 Hz, 2H), 7.19 (s, 2H), 4.77 (s, 1H), 1.80 (s, 3H) ppm.

6-Amino-1-(2-chlorophenyl)-3-methyl-4-(thiophen-3-yl)-1,4-dihydropyrano[2,3-c]-pyrazole-5-carbonitrile (3r): Synthesized with **1m** and **2b** according to the general procedure of **3a**. Yield: 65%, yellow power solid, m. p. 167.0–167.5°C. ^1^H NMR (400 MHz, DMSO-d_6_) δ 7.70 (dd, *J* = 7.7, 1.7 Hz, 1H), 7.62 (dd, *J* = 7.2, 2.2 Hz, 1H), 7.58–7.48 (m, 3H), 7.35 (dd, *J* = 2.8, 1.0 Hz, 1H), 7.01 (s, 2H), 6.91 (dd, *J* = 5.0, 1.1 Hz, 1H), 4.83 (s, 1H), 1.85 (s, 3H) ppm.

6-Amino-1-(2-chlorophenyl)-4-(3-methoxyphenyl)-3-methyl-1,4-dihydropyrano[2,3-c]-pyrazole-5-carbonitrile (3s): Synthesized with **1o** and **2b** according to the general procedure of **3a**. Yield: 80%, white power solid, m. p. 170.7–171.2°C. ^1^H NMR (400 MHz, DMSO-d_6_) δ 11.49 (s, 1H), 7.64–7.62 (m, 1H), 7.57–7.42 (m, 3H), 7.28 (t, *J* = 7.9 Hz, 2H), 7.16 (d, *J* = 7.2 Hz, 1H), 6.87 (dd, *J* = 8.2, 2.2 Hz, 1H), 5.85 (d, *J* = 9.2 Hz, 1H), 4.66 (d, *J* = 10.9 Hz, 1H), 3.74 (s, 3H), 2.14 (s, 3H) ppm.

6-Amino-1-(4-chlorophenyl)-3-methyl-4-phenyl-1,4-dihydropyrano[2,3-c]-pyrazole-5-carbonitrile (3t): Synthesized with **1a** and **2c** according to the general procedure of **3a**. Yield: 80%, white power solid, m. p. 165.9–166.4°C. ^1^H NMR (400 MHz, DMSO-d_6_) δ 7.83 (dt, *J* = 8.9, 3.0 Hz, 2H), 7.53 (dt, *J* = 8.9, 3.2 Hz, 2H), 7.38–7.32 (m, 2H), 7.29–7.19 (m, 5H), 4.68 (s, 1H), 1.78 (s, 3H) ppm.

6-Amino-1-(2-chlorophenyl)-3-methyl-4-phenyl-1,4-dihydropyrano[2,3-c]-pyrazole-5-carbonitrile (3u): Synthesized with **1a** and **2b** according to the general procedure of **3a**. Yield: 75%, white power solid, m. p. 188.9–189.2°C. 1H NMR (400 MHz, DMSO-d_6_) δ 11.64 (s, 1H), 7.67–7.56 (m, 3H), 7.51–7.46 (m, 3H) 7.37 (t, *J* = 7.4 Hz, 2H), 7.32–7.28 (m, 1H), 5.8 (d, *J* = 9.4 Hz, 1H), 4.70 (d, *J* = 10.2 Hz, 1H), 2.14 (s, 3H) ppm.

6-Amino-3-methyl-4-phenyl-1-(p-tolyl)-1,4-dihydropyrano-[2,3-c]-pyrazole-5-carbonitrile (3v): Synthesized with **1a** and **2d** according to the general procedure of **3a**. Yield: 90%, white power solid, m. p. 167.0–167.5°C. ^1^H NMR (400 MHz, DMSO-d_6_) δ 7.65 (dt, *J* = 8.5, 2.8 Hz, 2H), 7.35 (tt, *J* = 7.3, 1.0 Hz, 2H), 7.30–7.23 (m, 5H), 7.17 (s, 2H), 4.67 (s, 1H), 2.35 (s, 3H), 1.77 (s, 3H) ppm.

6-Amino-1-(4-methoxyphenyl)-3-methyl-4-phenyl-1,4-dihydropyrano[2,3-c]-pyrazole-5-carbonitrile (3w): Synthesized with **1a** and **2e** according to the general procedure of **3a**. Yield: 88%, white power solid, m. p. 186.2–187,3 °C. ^1^H NMR (400 MHz, DMSO-d_6_) δ 7.65 (dt, *J* = 8.5, 3.5 Hz, 2H), 7.38–7.32 (m, 2H), 7.29–7.23 (m, 3H), 7.14 (s, 2H), 7.06–7.01 (m, 2H), 4.67 (s, 1H), 3.80 (s, 3H), 1.77 (s, 3H) ppm.

6-Amino-1-(4-fluorophenyl)-3-methyl-4-phenyl-1,4-dihydropyrano[2,3-c]-pyrazole -5-carbonitrile (3x): Synthesized with **1a** and **2f** according to the general procedure of **3a**. Yield: 80%, white power solid, m. p. 162.9–163.5°C. ^1^H NMR (400 MHz, DMSO-d_6_) δ 7.84–7.77 (m, 2H), 7.37–7.31 (m, 4H), 7.29–7.24 (m, 3H), 7.19 (s, 2H), 4.68 (s, 1H), 1.77 (s, 3H) ppm.

N-(5-cyano-3-methyl-1,4-diphenyl-1,4-dihydropyrano[2,3-c]-pyrazole-6-yl)-4-methylbenzenesulfonamide (4a): A mixture of **3a** (5 mmol, 1.6 g), 4-methylbenzenesulfonyl chloride (5 mmol, 0.95 g), and N,N-dimethylpyridin-4-amine (DMAP) (5 mmol, 0.61 g) in 30 ml dichloromethane was stirred at room temperature overnight. TLC was used to monitor whether the reaction was complete. When the basic reaction of raw materials was complete, the stirring was stopped, and the reaction mixture was cooled to room temperature. The crude product was purified using silica gel column chromatography using a mixture solvent of petroleum ether/ethyl acetate (8:1) to give **4a**. Yield: 66%, white power solid, m. p. 68.3–69.7°C. ^1^H NMR (400 MHz, CDCl_3_) *δ* 7.50–7.42 (m, 4H), 7.41–7.38 (m, 1H), 7.35 (dt, *J* = 8.4, 2.5 Hz, 2H), 7.24–7.18 (m, 3H), 7.17–7.13 (m, 2H), 7.04 (dt, *J* = 7.7, 0.8 Hz, 2H), 4.98 (d, *J* = 9.6 Hz, 1H), 4.85 (d, *J* = 9.6 Hz, 1H), 2.37 (s, 3H), 2.15 (s, 3H) ppm; ^13^C NMR (100 MHz, CDCl_3_) *δ* 147.7, 147.2, 140.4, 137.1, 136.3, 130.7 (2C), 129.9, 129.4 (2C), 129.4 (2C), 128.6, 128.5 (2C), 128.0, 127.9 (2C), 123.3 (2C), 114.0, 113.9, 108.3, 40.3, 27.3, 21.7, 14.9 ppm. HRMS (ESI): calculated for C_27_H_22_N_4_NaO_3_S^+^ [M + Na]^+^, 505.1305; found 505.1304.

N-(5-cyano-3-methyl-1-phenyl-4-(p-tolyl)-1,4-dihydropyrano[2,3-c]-pyrazol-6-yl)-4-methylbenzenesulfonamide (4b): Synthesized with 3b according to the general procedure of **4a**. Yield: 70%, white power solid, m. p. 74.7–75.0°C. ^1^H NMR (400 MHz, CDCl_3_) *δ* 7.39–7.32 (m, 4H), 7.26–7.11 (m, 7H), 7.04 (d, *J* = 8.1 Hz, 2H), 4.96 (d, *J* = 9.7 Hz, 1H), 4.80 (d, *J* = 9.7 Hz, 1H), 2.37 (d, *J* = 1.7 Hz, 6H), 2.16 (s, 3H) ppm; ^13^C NMR (100 MHz, CDCl_3_) *δ* 147.7, 147.2, 140.4, 137.9, 137.1, 133.3, 130.7 (2C), 129.9 (2C), 129.9, 129.4 (2C), 128.5 (2C), 128.0, 127.7 (2C), 123.3 (2C), 114.0, 113.9, 108.4, 40.1, 27.3, 21.7, 21.1, 15.0 ppm. HRMS (ESI): calculated for C_28_H_24_N_4_NaO_3_S^+^ [M + Na]^+^, 519.1461; found 519.1462.

N-(4-(3-chlorophenyl)-5-cyano-3-methyl-1-phenyl-1,4-dihydropyrano[2,3-c]-pyrazol-6-yl)-4-methylbenzenesulfonamide (4c). Synthesized with **3c** according to the general procedure of **4a**. Yield: 68% white power solid. m. p. 68.4–69.0°C. ^1^H NMR (400 MHz, CDCl_3_) *δ* 7.45 (t, *J* = 1.6 Hz, 1H), 7.43–7.37 (m, 3H), 7.35 (dt, *J* = 8.4, 1.6 Hz, 2H), 7.25–7.12 (m, 5H), 7.05 (t, *J* = 7.8 Hz, 2H), 4.99 (d, *J* = 9.7 Hz, 1H), 4.81 (d, *J* = 9.7 Hz, 1H), 2.38 (s, 3H), 2.19 (s, 3H) ppm; ^13^C NMR (100 MHz, CDCl_3_) *δ* 148.0, 146.9, 139.9, 137.3, 137.1, 135.2, 130.4, 130.2, 129.9 (2C), 129.0, 128.8 (2C), 128.5 (2C), 128.2, 127.6, 125.9, 123.6 (2C), 112.0, 112.0, 107.0, 41.7, 27.1, 21.7, 14.3 ppm. HRMS (ESI): calculated for C_27_H_21_ClN_4_NaO_3_S^+^ [M + Na]^+^, 539.0915; found 539.0916.

N-(4-(4-bromophenyl)-5-cyano-3-methyl-1-phenyl-1,4-dihydropyrano[2,3-c]-pyrazol-6-yl)-4-methylbenzenesulfonamide (4d): Synthesized with **3d** according to the general procedure of **4a**. Yield: 70%, white power solid, m. p. 101.6–102.3°C. ^1^H NMR (400 MHz, CDCl_3_) *δ* 7.59 (dt, *J* = 8.5,2.8 Hz, 2H), 7.40–7.30 (m, 4H), 7.24–7.17 (m, 3H), 7.17–7.12 (m, 2H), 7.04 (dt, *J* = 7.8,1.8 Hz, 2H), 4.98 (d, *J* = 9.7 = Hz, 1H), 4.80 (d, *J* = 9.7 Hz, 1H), 2.37 (s, 3H), 2.17 (s, 3H) ppm; ^13^C NMR (100 MHz, CDCl_3_) *δ* 148.0, 146.9, 139.9, 137.1, 134.4, 132.4 (2C), 130.1, 129.9 (2C), 129.5 (2C), 128.8 (2C), 128.5 (2C), 127.6, 123.6 (2C), 122.9, 112.1, 112.1, 107.1, 41.7, 27.1, 21.8, 14.3 ppm. HRMS (ESI): calculated for C_27_H_21_BrN_4_NaO_3_S^+^ [M + Na]^+^, 583.0410; found 583.0410.

N-(5-cyano-4-(4-fluorophenyl)-3-methyl-1-phenyl-1,4-dihydropyrano[2,3-c]-pyrazol-6-yl)-4-methylbenzenesulfonamide (4e): Synthesized with **3e** according to the general procedure of **4a**. Yield: 70%, white power solid, m. p. 113.0–113.5°C. ^1^H NMR (400 MHz, CDCl_3_) *δ* 7.51–7.45 (m, 2H), 7.33 (dt, *J* = 8.4, 2.2 Hz, 2H), 7.24–7.18 (m, 3H), 7.17–7.11 (m, 4H), 7.05 (dt, *J* = 7.8, 0.8 Hz, 2H), 4.98 (d, *J* = 9.7 Hz, 1H), 4.82 (d, *J* = 9.7 Hz, 1H), 2.38 (s, 3H), 2.16 (s, 3H) ppm; ^13^C NMR (100 MHz, CDCl_3_) *δ* 162.1, 148.0, 146.9, 139.8, 137.1, 131.3, 130.2, 129.9 (2C), 129.7 (2C), 128.8 (2C), 128.5 (2C), 127.6, 123.7 (2C), 116.2 (2C), 112.2, 112.2, 107.4, 41.6, 27.4, 21.7, 14.3 ppm. HRMS (ESI): calculated for C_27_H_21_FN_4_NaO_3_S^+^ [M + Na]^+^, 523.1211; found 523.1216.

N-(4-(3-bromophenyl)-5-cyano-3-methyl-1-phenyl-1,4-dihydropyrano[2,3-c]-pyrazol-6-yl)-4-methylbenzenesulfonamide (4f): Synthesized with **3f** according to the general procedure of **4a.** Yield:75%, white power solid, m. p. 68.4–69.5°C. ^1^H NMR (400 MHz, CDCl_3_) *δ* 7.60 (t, *J* = 1.9 Hz, 1H), 7.54 (ddd, *J* = 8.0, 1.9, 1.0 Hz, 1H), 7.47 (ddd, *J* = 8.8, 1.9, 0.7 Hz, 1H), 7.38–7.30 (m, 3H), 7.24–7.18 (m, 3H), 7.18–7.12 (m, 2H), 7.05 (dt, *J* = 7.8, 0.8 Hz, 2H), 4.99 (d, *J* = 9.8 Hz, 1H), 4.80 (d, *J* = 9.8 Hz, 1H), 2.38 (s, 3H), 2.20 (s, 3H) ppm; ^13^C NMR (100 MHz, CDCl_3_) *δ* 148.0, 146.9, 139.9, 137.6, 137.1, 131.9, 131.1, 130.6, 130.1, 129.9 (2C), 128.8 (2C), 128.5 (2C), 127.6, 126.3, 123.6 (2C), 123.3, 112.0, 112.0, 107.0, 41.7, 27.1, 21.7, 14.3 ppm. HRMS (ESI): calculated for C_27_H_21_BrN_4_NaO_3_S^+^ [M + Na]^+^, 583.0410; found 583.0410.

*N*-(5-cyano-4-(3,4-dichlorophenyl)-3-methyl-1-phenyl-1,4-dihydropyrano[2,3-c]-pyrazol-6-yl)-4-methylbenzenesulfonamide (4g): Synthesized with **3g** according to the general procedure of **4a**. Yield: 69%, white power solid, m. p. 100.9–101.3°C. ^1^H NMR (400 MHz, CDCl_3_) *δ* 7.57 (d, *J* = 2.3 Hz, 1H), 7.55 (d, *J* = 8.4 Hz, 1H), 7.37 (dd, *J* = 8.4, 2.3 Hz, 1H), 7.33 (dt, *J* = 8.4, 2.3 Hz, 2H), 7.25–7.18 (m, 3H), 7.17–7.13 (m, 2H), 7.05 (dt, *J* = 7.7, 0.8 Hz, 2H), 4.99 (d, *J* = 9.9 Hz, 1H), 4.78 (d, *J* = 9.9 Hz, 1H), 2.38 (s, 3H), 2.21 (s, 3H) ppm; ^13^C NMR (100 MHz, CDCl_3_) *δ* 147.9, 147.0, 139.9, 137.0, 135.5, 133.5, 133.2, 131.1, 130.1, 130.1, 129.9 (2C), 128.9 (2C), 128.5 (2C), 127.7, 127.1, 123.7 (2C), 111.9, 111.8, 106.7, 41.4, 27.1, 21.8, 14.3 ppm. HRMS (ESI): calculated for C_27_H_20_Cl_2_N_4_NaO_3_S^+^ [M + Na]^+^, 573.0525; found 573.0523.

*N*-(4-(4-bromo-2-fluorophenyl)-5-cyano-3-methyl-1-phen-yl-1,4-dihydropyrano[2,3-c]-pyrazol-6-yl)-4-methylbenzenesulfonamide (4h): Synthesized with **3h** according to the general procedure of **4a**. Yield: 66%, white power solid, m. p. 73.6–77.4°C. ^1^H NMR (400 MHz, CDCl_3_) *δ* 7.52 (t, *J* = 8.1 Hz, 1H), 7.45 (dd, J = 8.5, 1.9 Hz, 1H), 7.35 (dd, *J* = 9.8, 1.9 Hz, 1H), 7.30 (dt, *J* = 8.4, 2.2 Hz, 2H), 7.22–7.14 (m, 3H), 7.12–7.06 (m, 2H), 7.02 (dt, *J* = 8.5, 2.0 Hz, 2H), 5.24 (d, *J* = 10.9 Hz, 1H), 5.03 (d, J = 10.9 Hz, 1H), 2.37 (s, 3H), 2.32 (s, 3H) ppm; ^13^C NMR (100 MHz, CDCl_3_) *δ* 160.0, 148.4, 146.9, 139.9, 137.0, 130.0, 129.9 (2C), 129.5, 128.8 (2C), 128.5 (2C), 128.2, 127.5, 123.6 (2C), 123.3, 122.0, 120.0, 111.9, 111.9, 105.9, 35.5, 26.4, 21.7, 13.8 ppm. HRMS (ESI): calculated for C_27_H_20_BrFN_4_NaO_3_S^+^ [M + Na]^+^, 601.0316; found 601.0311.

*N*-(5-cyano-4-(4-methoxyphenyl)-3-methyl-1-phenyl-1,4-dihydropyrano[2,3-c]-pyrazol-6-yl)-4-methylbenzenesulfonamide (4i): Synthesized with **3i** according to the general procedure of **4a**. Yield: 80%, white power solid, m. p. 145.2–146.0°C. ^1^H NMR (400 MHz, CDCl_3_) *δ* 7.40 (dt, *J* = 8.6, 3.4 Hz, 2H), 7.34 (dt, *J* = 8.4, 2.2 Hz, 2H), 7.24–7.18 (m, 3H), 7.17–7.13 (m, 2H), 7.04 (dt, J = 7.7, 1.6 Hz, 2H), 6.96 (dt, *J* = 8.8, 3.2 Hz, 2H), 4.95 (d, *J* = 9.7 Hz, 1H), 4.78 (d, *J* = 9.7 Hz, 1H), 3.83 (s, 3H), 2.37 (s, 3H), 2.16 (s, 3H) ppm; ^13^C NMR (100 MHz, CDCl_3_) *δ* 159.4, 147.8, 147.2, 140.3, 137.1, 130.8 (2C), 129.9, 129.5 (2C), 129.2 (2C), 128.6 (2C), 128.2, 128.1, 123.3 (2C), 114.80 (2C), 114.1, 114.0, 108.6, 55.7, 39.8, 27.6, 21.7, 15.0 ppm. HRMS (ESI): calculated for C_28_H_24_N_4_NaO_4_S^+^ [M + Na]^+^, 535.1410; found 535.1417.

*N*-(5-cyano-4-(furan-2-yl)-3-methyl-1-phenyl-1,4-dihydropyrano[2,3-c]-pyrazol-6-yl)-4-methylbenzenesulfonamide (4j): Synthesized with **3j** according to the general procedure of **4a**. Yield: 55%, yellow power solid, m. p. 153.8–154.3°C. ^1^H NMR (400 MHz, CDCl_3_) *δ* 7.49 (d, *J* = 1.4 Hz, 1H), 7.35 (d, *J* = 8.4 Hz, 2H), 7.26 (s, 1H), 7.24–7.18 (m, 3H), 7.4–7.12 (m, 2H), 7.01 (d, *J* = 8.2 Hz, 2H), 6.52 (d, *J* = 3.3 Hz, 1H), 6.45 (dd, *J* = 3.3, 1.9 Hz, 1H), 4.98 (d, *J* = 8.9 Hz, 1H), 4.84 (d, *J* = 8.9 Hz, 1H), 2.29 (s, 3H) ppm; ^13^C NMR (100 MHz, DMSO-d_6_) *δ* 149.1, 147.7, 147.5, 144.1, 140.5, 136.9, 130.7 (2C), 129.7, 129.4 (2C), 128.5 (2C), 128.0, 123.2 (2C), 113.7, 113.4, 111.4, 109.3, 106.0, 35.2, 27.2, 21.7, 14.4 ppm. HRMS (ESI): calculated for C_25_H_20_N_4_NaO_4_S^+^ [M + Na]^+^, 495.1097; found 495.1094.

*N*-(5-cyano-3-methyl-1-phenyl-4-(pyridin-3-yl)-1,4-dihydropyrano[2,3-c]-pyrazol-6-yl)-4-methylbenzenesulfonamide (4k): Synthesized with **3k** according to the general procedure of **4a**. Yield: 60%, white power solid, m. p. 136.3–137.0°C. ^1^H NMR (400 MHz, DMSO-d_6_) *δ* 7.98 (s, 1H), 7.87 (dd, *J* = 8.7, 1.0 Hz, 1H), 7.58 (d, *J* = 8.2 Hz, 2H), 7.52–7.38 (m, 5H), 7.34 (d, *J* = 8.0 Hz, 3H), 7.12 (d, *J* = 7.8 Hz, 3H), 2.36 (s, 3H), 2.29 (s, 3H) ppm; ^13^C NMR (100 MHz, DMSO-d_6_) *δ* 161.6, 151.9, 148.9, 147.7, 146.0, 145.0, 144.3, 142.4, 141.8, 138.2, 129.9 (2C), 129.4, 128.6 (2C), 126.0 (2C), 125.3 (2C), 123.4, 119.8, 118.9, 62.6, 21.4, 21.2, 13.5 ppm. HRMS (ESI): calculated for C_26_H_21_N_5_NaO_3_S^+^ [M + Na]^+^, 506.1257; found 506.1272.

*N*-(5-cyano-3-methyl-1-phenyl-4-(pyridin-4-yl)-1,4-dihydropyrano[2,3-c]-pyrazol-6-yl)-4-methylbenzenesulfonamide (4l): Synthesized with **3l** according to the general procedure of **4a**. Yield: 60%, white power solid, m. p. 100.5–101.1°C. ^1^H NMR (400 MHz, DMSO-d_6_) *δ* 8.70 (dd, *J* = 4.7, 1.4 Hz, 2H), 7.48 (d, *J* = 5.8 Hz, 2H), 7.43 (d, *J* = 8.4 Hz, 2H), 7.36–7.28 (m, 3H), 7.18 (d, *J* = 8.1 Hz, 2H), 7.14–7.07 (m, 2H), 5.95 (d, *J* = 8.9 Hz, 1H), 5.09 (d, *J* = 8.9 Hz, 1H), 2.34 (s, 3H), 2.03 (s, 3H) ppm; ^13^C NMR (100 MHz, DMSO-d_6_) *δ* 150.3, 147.7, 147.4, 145.7, 140.9, 136.9, 130.7 (2C), 129.7, 129.5 (2C), 128.6 (2C), 128.1, 123.3 (2C), 123.2 (2C), 113.7, 113.6, 107.0, 100.0, 39.5, 27.0, 21.7, 14.9 ppm. HRMS (ESI): calculated for C_26_H_21_N_5_NaO_3_S^+^ [M + Na]^+^, 506.1257; found 506.1253.

*N*-(5-cyano-3-methyl-1-phenyl-4-(thiophen-3-yl)-1,4-dihydropyrano[2,3-c]-pyrazol-6-yl)-4-methylbenzenesulfonamide (4m): Synthesized with **3m** according to the general procedure of **4a**. Yield: 65%, yellow power solid, m. p. 67.8–68.4°C. ^1^H NMR (400 MHz, DMSO-d_6_) *δ* 7.68 (dd, *J* = 5.0, 2.9 Hz, 1H), 7.61–7.57 (m, 1H), 7.43 (d, *J* = 8.4 Hz, 2H), 7.36–7.26 (m, 3H), 7.18 (d, *J* = 8.1 Hz, 2H), 7.15–7.05 (m, 3H), 5.82 (d, *J* = 8.9 Hz, 1H), 4.94 (d, *J* = 8.9 Hz, 1H), 2.34 (s, 3H), 2.00 (s, 3H) ppm; ^13^C NMR (100 MHz, DMSO-d_6_) *δ* 147.7, 147.4, 140.4, 137.0, 136.9, 130.7 (2C), 129.8, 129.4 (2C), 128.5 (2C), 128.1, 128.0, 127.8, 123.5, 123.2 (2C), 114.0, 113.8, 108.0, 36.8, 28.0, 21.7, 14.6 ppm. HRMS (ESI): calculated for C_25_H_20_N_4_NaO_3_S_2_^+^ [M + Na]^+^, 511.0869; found 511.0868.

*N*-(5-cyano-3-methyl-1-phenyl-4-(4-(trifluoromethyl)phenyl)-1,4-dihydropyrano[2,3-c]-pyrazol-6-yl)-4-methylbenzenesulfonamide (4n): Synthesized with **3n** according to the general procedure of **4a**. Yield: 73%, white power solid, m. p. 109.3–110.2°C. ^1^H NMR (400 MHz, DMSO-d_6_) *δ* 7.89 (d, *J* = 8.3 Hz, 2H), 7.70 (d, *J* = 8.2 Hz, 2H), 7.44 (d, *J* = 8.3 Hz, 2H), 7.36–7.30 (m, 3H), 7.18 (d, *J* = 8.2 Hz, 2H), 7.11 (dd, *J* = 7.6, 1.9 Hz, 2H), 5.97 (d, *J* = 9.3 Hz, 1H), 5.12 (d, *J* = 9.3 Hz, 1H), 2.34 (s, 3H), 2.07 (s, 3H) ppm; ^13^C NMR (100 MHz, DMSO-d_6_) *δ* 148.0, 147.1, 140.0, 139.4, 137.7, 137.0, 131.0, 130.0 (2C), 129.0 (2C), 128.5 (2C), 128.5 (2C), 127.7, 126.3 (2C), 123.8, 123.7 (2C), 112.1, 112.1, 107.0, 41.9, 27.1, 21.9, 14.4 ppm. HRMS (ESI): calculated for C_28_H_21_F_3_N_4_NaO_3_S^+^ [M + Na]^+^, 573.1179; found 573.1176.

*N*-(5-cyano-3-methyl-1-phenyl-4-(4-(trifluoromethyl)phenyl)-1,4-dihydropyrano[2,3-c]-pyrazol-6-yl)thiophene-2-sulfonamide (4o): Synthesized with **3n** and 4-thiophene-2-sulfonyl chloride according to the general procedure of **4a**. Yield: 54%, white power solid, m. p. 55.9–56.7°C. ^1^H NMR (400 MHz, DMSO-d_6_) *δ* 8.12 (dd, *J* = 5.0, 1.3 Hz, 1H), 7.89 (d, *J* = 8.3 Hz, 2H), 7.70 (d, *J* = 8.2 Hz, 2H), 7.55 (dd, *J* = 3.9, 1.3 Hz, 1H), 7.41–7.34 (m, 3H), 7.24–7.18 (m, 2H), 7.04 (dd, *J* = 4.9, 4.0 Hz, 1H), 5.98 (d, *J* = 9.2 Hz, 1H), 5.17 (d, *J* = 9.2 Hz, 1H), 2.07 (s, 3H) ppm; ^13^C NMR (100 MHz, DMSO-d_6_) *δ* 148.1, 139.9, 139.2, 137.3, 137.0, 136.9, 131.9, 131.0, 129.2 (2C), 128.4 (2C), 128.2, 128.0, 126.3 (2C), 123.8, 123.6 (2C), 112.0, 111.9, 107.1, 41.7, 27.0, 14.5 ppm. HRMS (ESI): calculated for C_25_H_17_F_3_N_4_NaO_3_S_2_^+^ [M + Na]^+^, 565.0586; found 565.0572.

*N*-(5-cyano-3-methyl-1-phenyl-4-(4-(trifluoromethyl) phenyl)-1,4-dihydropyrano[2,3-c]-pyrazol-6-yl)-4-methoxybenzenesulfonamide (4p): Synthesized with **3n** and 4-methoxybenzenesulfonyl chloride according to the general procedure of **4a**. Yield: 76%, white power solid, m. p. 72.0–72.7°C. ^1^H NMR (400 MHz, DMSO-d_6_) *δ* 7.89 (d, *J* = 8.3 Hz, 2H), 7.71 (d, *J* = 8.2 Hz, 2H), 7.47 (d, *J* = 9.0 Hz, 2H), 7.36–7.28 (m, 3H), 7.14 (dd, *J* = 7.8, 1.8 Hz, 2H), 6.86 (d, *J* = 9.0 Hz, 2H), 5.98 (d, *J* = 9.3 Hz, 1H), 5.15 (d, *J* = 9.3 Hz, 1H), 3.83 (s, 3H), 2.07 (s, 3H) ppm; ^13^C NMR (100 MHz, DMSO-d_6_) *δ* 165.1, 148.0, 140.2, 139.4, 137.1, 137.1, 131.0, 130.9 (2C), 129.0 (2C), 128.5 (2C), 127.8, 126.3 (2C), 123.8, 123.6 (2C), 114.6 (2C), 112.2, 112.1, 107.0, 55.9, 41.9, 27.1, 14.4 ppm. HRMS (ESI): calculated for C_28_H_21_F_3_N_4_NaO_4_S^+^ [M + Na]^+^, 589.1128; found 589.1129.

*N*-(5-cyano-3-methyl-1-phenyl-4-(4-(trifluoromethyl)phenyl)-1,4-dihydropyrano[2,3-c]-pyrazol-6-yl)-4-(trifluoromethyl)-benzenesulfonamide (4q): Synthesized with **3n** and 4-(trifluoromethyl) benzenesulfonyl chloride according to the general procedure of **4a**. Yield: 62%, yellow power solid, m. p. 153.7–154.1°C. ^1^H NMR (400 MHz, DMSO-d_6_) *δ* 7.86 (d, *J* = 8.3 Hz, 2H), 7.83–7.76 (m, 5H), 7.73–7.70 (m, 4H), 7.48–7.44 (m, 2H), 6.00 (d, *J* = 11.3 Hz, 1H), 4.94 (d, *J* = 11.3 Hz, 1H), 2.24 (s, 3H) ppm; ^13^C NMR (100 MHz, DMSO-d_6_) *δ* 148.2, 139.7, 139.1, 136.8, 136.7, 136.7, 131.1, 129.3 (2C), 129.1 (2C), 128.3 (2C), 128.2, 126.4 (4C), 123.8, 123.6 (2C), 120.9, 111.9, 111.9, 107.3, 41.8, 27.1, 14.5 ppm. HRMS (ESI): calculated for C_28_H_18_F_6_N_4_NaO_3_S^+^ [M + Na]^+^, 627.0896; found 627.0880.

*N*-(5-cyano-3-methyl-1-phenyl-4-(4-(trifluoromethyl)phenyl)-1,4-dihydropyrano[2,3-c]-pyrazol-6-yl)-2,4-difluorobenzenesulfonamide (4r): Synthesized with **3n** and 2,4-difluorobenzenesulfonyl chloride according to the general procedure of **4a**. Yield: 60%, white power solid, m. p. 65.8–66.5°C. ^1^H NMR (400 MHz, DMSO-d_6_) *δ* 7.89 (d, *J* = 8.3 Hz, 2H), 7.71 (d, *J* = 8.3 Hz, 2H), 7.67–7.61 (m, 1H), 7.37–7.27 (m, 4H), 7.20–7.11 (m, 3H), 5.96 (d, *J* = 9.0 Hz, 1H), 5.21 (d, *J* = 9.0 Hz, 1H), 2.05 (s, 3H) ppm; ^13^C NMR (100 MHz, DMSO-d_6_) *δ* 167.7, 160.5, 148.3, 139.5, 139.1, 136.8, 133.0, 132.9, 130.0, 129.2 (2C), 128.3 (2C), 128.2, 126.4 (2C), 123.8 (1C), 123.6 (2C), 112.6, 112.0, 111.8, 107.4, 106.4, 41.7, 27.0, 14.4 ppm. HRMS (ESI): calculated for C_27_H_17_F_5_N_4_NaO_3_S^+^ [M + Na]^+^, 595.0834; found 595.0822.

*N*-(1-(2-chlorophenyl)-5-cyano-4-(furan-2-yl)-3-methyl-1,4-dihydropyrano[2,3-c]-pyrazol-6-yl)-4-methylbenzenesulfonamide (4s): Synthesized with **3o** according to the general procedure of **4a**. Yield: 50%, yellow power solid, m. p. 62.5–63.9°C. ^1^H NMR (400 MHz, DMSO-d_6_) *δ* 7.77 (d, *J* = 1.2 Hz, 1H), 7.50 (dd, *J* = 15.6, 7.7, 1.4 Hz, 2H), 7.45 (dt, *J* = 8.4, 1.5 Hz, 2H), 7.34 (td, *J* = 7.7, 1.5 Hz, 1H), 7.30 (d, *J* = 8.2 Hz, 2H), 7.08 (d, *J* = 7.1 Hz, 1H), 6.55 (dd, *J* = 3.2, 1.9 Hz, 1H), 6.48 (d, *J* = 3.2 Hz, 1H), 5.69 (d, *J* = 9.1 Hz, 1H), 4.88 (d, *J* = 9.1 Hz, 1H), 2.40 (s, 3H), 2.12 (s, 3H) ppm; ^13^C NMR (100 MHz, DMSO-d_6_) *δ* 149.0, 148.1, 147.6, 144.0, 141.8, 134.3, 131.4, 131.0 (2C), 130.5, 130.5, 130.4, 129.7, 128.4, 128.3 (2C), 113.5, 113.3, 111.4, 109.3, 105.2, 35.1, 27.2, 21.7, 14.3 ppm. HRMS (ESI): calculated for C_25_H_19_ClN_4_NaO_4_S^+^ [M + Na]^+^, 529.0708; found 529.0703.

*N*-(1-(2-chlorophenyl)-5-cyano-3-methyl-4-(pyridin-3-yl)-1,4-dihydropyrano[2,3-c]-pyrazol-6-yl)-4-methylbenzenesulfonamide (4t): Synthesized with **3p** according to the general procedure of **4a**. Yield: 51%, white power solid, m. p. 195.8–196.3°C. ^1^H NMR (400 MHz, DMSO-d_6_) *δ* 8.66 (d, *J* = 1.6 Hz, 1H), 8.62 (d, *J* = 4.6 Hz, 1H), 7.87 (d, *J* = 8.1 Hz, 1H), 7.54 (dd, *J* = 7.9, 4.8 Hz, 1H), 7.51–7.42 (m, 4H), 7.37–7.32 (m, 1H), 7.29 (d, *J* = 8.3 Hz, 2H), 7.14 (d, *J* = 7.7 Hz, 1H), 5.89 (d, *J* = 9.8 Hz, 1H), 5.05 (d, *J* = 9.8 Hz, 1H), 2.39 (s, 3H), 2.10 (s, 3H) ppm; ^13^C NMR (100 MHz, DMSO-d_6_) *δ* 149.6, 149.1, 147.9, 147.6, 141.7, 135.9, 134.4, 132.3, 131.4, 131.0 (2C), 130.4 (2C), 129.8, 128.5, 128.5, 128.3 (2C), 124.3, 113.6, 113.5, 106.6, 38.2, 27.3, 21.7, 14.9 ppm. HRMS (ESI): calculated for C_26_H_20_ClN_5_NaO_3_S^+^ [M + Na]^+^, 540.0868; found 540.0870.

*N*-(1-(2-chlorophenyl)-5-cyano-3-methyl-4-(pyridin-4-yl)-1,4-dihydropyrano[2,3-c]-pyrazol-6-yl)-4-methylbenzenesulfonamide (4u): Synthesized with **3q** according to the general procedure of **4a**. Yield: 53%, white power solid, m. p. 79.0–80.0°C. 1H NMR (400 MHz, DMSO-d_6_) *δ* 8.69 (dd, *J* = 4.6, 1.5 Hz, 2H), 7.52–7.42 (m, 6H), 7.37–7.31 (m, 1H), 7.29 (d, *J* = 8.2 Hz, 2H), 7.11 (dd, *J* = 7.9, 1.3 Hz, 1H), 5.90 (d, *J* = 9.2 Hz, 1H), 5.03 (d, *J* = 9.2 Hz, 1H), 2.39 (s, 3H), 2.04 (s, 3H) ppm; ^13^C NMR (100 MHz, DMSO-d_6_) *δ* 150.5 (2C), 148.0, 147.6, 145.3, 142.0, 134.3, 131.4, 131.0 (2C), 130.4, 130.4, 130.4, 129.7, 128.4, 128.3 (2C), 123.1 (2C), 113.5, 113.5, 106.2, 39.3, 26.9, 21.7, 14.9 ppm. HRMS (ESI): calculated for C_26_H_20_ClN_5_NaO_3_S^+^ [M + Na]^+^, 540.0868; found 540.0867.

*N*-(1-(2-chlorophenyl)-5-cyano-3-methyl-4-(thiophen-3-yl)-1,4-dihydropyrano[2,3-c]-pyrazol-6-yl)-4-methylbenzenesulfonamide (4v): Synthesized with **3r** according to the general procedure of **4a**. Yield: 57%, yellow power solid, m. p. 67.4–68.8°C. ^1^H NMR (400 MHz, DMSO-d_6_) *δ* 7.67 (dd, *J* = 5.0, 2.9 Hz, 1H), 7.59–7.55 (m, 1H), 7.52–7.44 (m, 4H), 7.33 (td, *J* = 7.7, 1.6 Hz, 1H), 7.29 (d, *J* = 8.2 Hz, 2H), 7.11–7.05 (m, 2H), 5.79 (d, *J* = 9.3 Hz, 1H), 4.88 (d, *J* = 9.4 Hz, 1H), 2.40 (s, 3H), 2.00 (s, 3H) ppm; ^13^C NMR (100 MHz, DMSO-d_6_) *δ* 148.0, 147.6, 141.5, 136.8, 134.4, 131.3, 131.0 (2C), 130.4 (2C), 129.7, 128.4, 128.3 (2C), 128.2, 128.0, 127.8, 123.5, 113.9, 113.6, 107.1, 36.7, 27.9, 21.7, 14.6 ppm. HRMS (ESI): calculated for C_25_H_19_ClN_4_NaO_3_S_2_^+^ [M + Na]^+^, 545.0479; found 545.0485.

*N*-(1-(2-chlorophenyl)-5-cyano-4-(3-methoxyphenyl)-3-methyl-1,4-dihydropyrano[2,3-c]-pyrazol-6-yl)-4-methylbenzenesulfonamide (4w): Synthesized with **3s** according to the general procedure of **4a**. Yield: 68%, white power solid, m. p. 66.4–67.7°C. ^1^H NMR (400 MHz, CDCl_3_) *δ* 7.36–7.27 (m, 7H), 7.15 (d, *J* = 8.2 Hz, 2H), 7.06 (d, *J* = 7.8 Hz, 1H), 7.05 (t, *J* = 1.8 Hz, 1H), 6.91 (dd, *J* = 8.3, 2.2 Hz, 1H), 4.93 (d, *J* = 10.0 Hz, 1H), 4.69 (d, *J* = 10.0 Hz, 1H), 3.82 (s, 3H), 2.42 (s, 3H), 2.13 (s, 3H) ppm; ^13^C NMR (100 MHz, CDCl_3_) *δ* 160.2, 148.8, 146.6, 140.6, 136.9, 134.8, 131.9, 131.0, 130.2, 130.2, 130.1, 130.1 (2C), 129.8, 128.0 (2C), 127.3, 120.3, 114.6, 113.5, 112.3, 112.0, 106.7, 5.4, 42.4, 27.1, 21.8, 14.1 ppm. HRMS (ESI): calculated for C_28_H_23_ClN_4_NaO_4_S^+^ [M + Na]^+^, 569.1021; found 569.1022.

*N*-(1-(2-chlorophenyl)-5-cyano-4-(3-methoxyphenyl)-3-methyl-1,4-dihydropyrano[2,3-c]-pyrazol-6-yl)benzenesulfonamide (4x). Synthesized with **3s** and benzenesulfonyl chloride according to the general procedure of **4a**. Yield: 70%, white power solid, m. p. 66.0–67.5°C. ^1^H NMR (400 MHz, CDCl_3_) *δ* 7.63 (t, *J* = 7.4 Hz, 1H), 7.49–7.44 (m, 2H), 7.41–7.26 (m, 7H), 7.05 (dd, *J* = 13.9, 4.9 Hz, 2H), 7.07 (d, *J* = 7.6 Hz, 1H), 7.03 (t, *J* = 2.0 Hz, 1H), 4.92 (d, *J* = 10.1 Hz, 1H), 4.70 (d, *J* = 10.1 Hz, 1H), 2.14 (s, 3H), 1.56 (s, 3H) ppm; ^13^C NMR (100 MHz, DMSO-d_6_) *δ* 159.9, 148.0, 141.4, 137.7, 136.4, 134.4, 133.9, 133.7, 131.6, 130.6, 130.5 (2C), 129.9, 128.6, 128.2 (2C), 128.1, 120.1, 114.2, 113.8, 113.6, 107.4, 65.7, 55.6, 46.4, 27.2, 14.8 ppm. HRMS (ESI): calculated for C_27_H_21_ClN_4_NaO_4_S^+^ [M + Na]^+^, 555.0864; found 555.0869.

*N*-(1-(2-chlorophenyl)-5-cyano-4-(3-methoxyphenyl)-3-methyl-1,4-dihydropyrano[2,3-c]-pyrazol-6-yl)-4-methoxybenzenesulfonamide (4y): Synthesized with **3s** and 4-methoxybenzenesulfonyl chloride according to the general procedure of **4a**. Yield: 64%, white power solid, m. p. 67.6–68.2°C. ^1^H NMR (400 MHz, DMSO-d_6_) *δ* 7.53–7.45 (m, 4H), 7.42–7.34 (m, 2H), 7.19–7.16 (m, 1H), 7.07–6.93 (m, 5H), 5.86 (d, *J* = 10.1 Hz, 1H), 4.86 (d, *J* = 10.2 Hz, 1H), 3.87 (s, 3H), 3.78 (s, 3H), 2.05 (s, 3H) ppm; ^13^C NMR (100 MHz, DMSO-d_6_) *δ* 165.3, 159.9, 147.9, 141.7, 137.7, 134.5, 131.5, 130.7 (2C), 130.5, 130.4, 129.9, 128.5, 124.3, 120.1, 115.8 (2C), 115.1, 114.2, 113.9, 113.7, 113.6, 107.3, 65.7, 56.6, 55.6, 27.2, 14.8 ppm. HRMS (ESI): calculated for C_28_H_23_N_4_NaO_5_S^+^ [M + Na]^+^, 585.0970; found 585.0973.

*N*-(1-(2-chlorophenyl)-5-cyano-4-(3-methoxyphenyl)-3-methyl-1,4-dihydropyrano[2,3-c]-pyrazol-6-yl)-2-nitrobenzenesulfonamide (4z): Synthesized with **3s** and 2-nitrobenzenesulfonyl chloride according to the general procedure of **4a**. Yield: 68%, white power solid. m. p. 72.6–73.7°C. ^1^H NMR (400 MHz, CDCl_3_) *δ* 7.83 (dd, *J* = 7.9, 1.2 Hz, 1H), 7.79 (td, *J* = 7.1, 1.4 Hz, 1H), 7.79 (td, *J* = 7.9, 1.3 Hz, 1H), 7.51 (dd, *J* = 7.9, 1.0 Hz, 1H), 7.36 (t, *J* = 8.0 Hz, 1H), 7.31 (d, *J* = 7.1 Hz, 1H), 7.26–7.17 (m, 3H), 7.07 (d, *J* = 7.5 Hz, 1H), 6.99 (t, *J* = 1.9 Hz, 1H), 6.92 (dd, *J* = 8.2, 2.2 Hz, 1H), 4.82 (d, *J* = 10.3 Hz, 1H), 4.72 (d, *J* = 10.3 Hz, 1H), 3.82 (s, 3H), 2.18 (s, 3H) ppm; ^13^C NMR (100 MHz, DMSO-d_6_) *δ* 159.9, 148.1, 147.5, 141.3, 138.2, 137.5, 134.3, 134.1, 131.8, 131.6, 131.0, 130.5, 130.4, 130.3, 129.6, 128.4, 126.4, 126.3, 122.8, 120.0, 114.1, 113.8, 113.6, 107.7, 55.6, 27.3, 14.9 ppm. HRMS (ESI): calculated for C_27_H_20_N_5_NaO_6_S^+^ [M + Na]^+^, 600.0715; found 600.0720.

*N*-(1-(2-chlorophenyl)-5-cyano-4-(3-methoxyphenyl)-3-methyl-1,4-dihydropyrano[2,3-c]-pyrazol-6-yl)-3-nitrobenzenesulfonamide (4aa): Synthesized with **3s** and 3-nitrobenzenesulfonyl chloride according to the general procedure of **4a**. Yield: 73%, white power solid, m. p. 78.9–79.2°C. ^1^H NMR (400 MHz, CDCl_3_) *δ* 8.44 (ddd, *J* = 8.2, 2.1, 0.9 Hz, 1H), 8.14 (t, *J* = 1.8 Hz, 1H), 7.97 (dt, *J* = 7.9, 1.0 Hz, 1H), 7.67 (t, *J* = 8.1 Hz, 1H), 7.38 (t, *J* = 8.0 Hz, 1H), 7.32 (d, *J* = 9.0 Hz, 1H), 7.26–7.18 (m, 3H), 7.05 (d, *J* = 7.7 Hz, 1H), 7.00 (t, *J* = 2.0 Hz, 1H), 6.93 (dd, *J* = 8.2, 2.2 Hz, 1H), 4.83 (d, *J* = 10.1 Hz, 1H), 4.76 (d, *J* = 10.1 Hz, 1H), 3.83 (s, 3H), 2.16 (s, 3H) ppm; ^13^C NMR (100 MHz, DMSO-d_6_) *δ* 159.9, 148.2, 141.0, 137.5, 135.2, 134.2, 134.1, 132.8, 132.4, 131.6, 130.7, 130.5, 130.4, 130.2, 129.8, 128.7, 123.8, 122.8, 120.5, 120.1, 114.2, 113.8, 113.7, 107.6, 55.6, 27.4, 14.8 ppm. HRMS (ESI): calculated for C_27_H_20_N_5_NaO_6_S^+^ [M + Na]^+^, 600.0715; found 600.0717.

*N*-(1-(2-chlorophenyl)-5-cyano-4-(3-methoxyphenyl)-3-methyl-1,4-dihydropyrano[2,3-c]-pyrazol-6-yl)-4-nitrobenzenesulfonamide (4ab). Synthesized with **3s** and 4-nitrobenzenesulfonyl chloride according to the general procedure of **4a**. Yield: 60%, white power solid, m. p. 142.5–143.6°C. ^1^H NMR (400 MHz, CDCl_3_) *δ* 8.16 (dt, J = 8.8, 2.2 Hz, 2H), 7.67 (dt, *J* = 8.8, 2.2 Hz, 2H), 7.37 (t, *J* = 8.0 Hz, 1H), 7.32 (dd, *J* = 7.2, 2.0 Hz, 1H), 7.28–7.24 (m, 3H), 7.04 (d, *J* = 7.7 Hz, 1H), 6.99 (t, *J* = 2.0 Hz, 1H), 6.93 (dd, *J* = 8.2, 2.3 Hz, 1H), 4.82 (d, *J* = 10.0 Hz, 1H), 4.75 (d, *J* = 10.0 Hz, 1H), 3.83 (s, 3H), 2.16 (s, 3H) ppm; ^13^C NMR (100 MHz, DMSO-d_6_) *δ* 159.9, 151.6, 148.2, 138.8, 137.4, 134.3, 131.7, 130.6, 130.5, 130.4, 130.0 (2C), 129.9, 128.6, 127.4, 125.5 (2C), 123.8, 120.1, 114.3, 113.8, 113.7, 113.6, 107.6, 55.6, 27.4, 14.9 ppm. HRMS (ESI): calculated for C_27_H_21_N_5_O_6_S^+^ [M + H]^+^, 578.0896; found 578.0885.

*N*-(1-(4-chlorophenyl)-5-cyano-3-methyl-4-phenyl-1,4-dihydropyrano[2,3-c]-pyrazol-6-yl)-4-methylbenzenesulfonamide (4ac): Synthesized with **3t** according to the general procedure of **4a**. Compound (**4ac**) was synthesized from compound (**3t**) and 4-methylbenzenesulfonyl chloride, in a manner similar to (**4a**). Yield: 75%, white power solid. m. p. 181.4–181.7°C. ^1^H NMR (400 MHz, CDCl_3_) *δ* 7.49–7.36 (m, 7H), 7.19–7.07 (m, 6H), 4.95 (d, *J* = 9.6 Hz, 1H), 4.82 (d, *J* = 9.6 Hz, 1H), 2.43 (s, 3H), 2.15 (s, 3H) ppm; ^13^C NMR (100 MHz, CDCl_3_) *δ* 148.5, 147.2, 140.1, 135.6, 135.2, 133.4, 130.7, 129.9 (2C), 129.2 (2C), 128.9 (2C), 128.7, 128.4 (2C), 127.8 (2C), 124.7 (2C), 112.2, 112.2, 108.1, 42.0, 27.2, 21.8, 14.3 ppm. HRMS (ESI): calculated for C_27_H_21_ClN_4_NaO_3_S^+^ [M + Na]^+^, 539.0915; found 539.0914.

*N*-(1-(2-chlorophenyl)-5-cyano-3-methyl-4-phenyl-1,4-dihydropyrano[2,3-c]-pyrazol-6-yl)-4-methylbenzenesulfonamide (4ad). Synthesized with **3u** according to the general procedure of **4a**. Yield: 60%, white power solid, m. p. 77.4–78.0°C. ^1^H NMR (400 MHz, CDCl_3_) *δ* 7.50–7.41 (m, 4H), 7.41–7.33 (m, 3H), 7.32–7.29 (m, 2H), 7.16 (d, *J* = 8.1 Hz, 2H), 4.93 (d, *J* = 9.7 Hz, 1H), 4.74 (d, *J* = 9.6 Hz, 1H), 2.43 (s, 3H), 2.10 (s, 3H) ppm; ^13^C NMR (100 MHz, CDCl_3_) *δ* 148.8, 146.6, 140.7, 135.4, 134.4, 131.8, 131.0, 130.2, 130.1, 130.1 (2C), 129.7, 129.2 (2C), 128.6, 128.1 (2C), 127.9 (2C), 127.3, 112.3, 112.2, 106.9, 42.2, 27.1, 21.8, 14.2 ppm. HRMS (ESI): calculated for C_27_H_21_ClN_4_NaO_3_S^+^ [M + Na]^+^, 539.0915; found 539.0916.

*N*-(5-cyano-3-methyl-4-phenyl-1-(p-tolyl)-1,4-dihydro-pyrano[2,3-c]-pyrazol-6-yl)-4-methylbenzenesulfonamide (4ae). Synthesized with **3v** according to the general procedure of **4a**. Yield: 70%, white power solid. m. p. 142.5–143.0°C. ^1^H NMR (400 MHz, CDCl_3_) *δ* 7.50–7.42 (m, 4H), 7.40–7.33 (m, 3H), 7.07–6.97 (m, 6H), 4.97 (d, *J* = 9.6 Hz, 1H), 4.82 (d, *J* = 9.6 Hz, 1H), 2.39 (s, 3H), 2.34 (s, 3H), 2.14 (s, 3H) ppm; ^13^C NMR (100 MHz, CDCl_3_) *δ* 147.9, 147.6, 141.7, 136.3, 134.5, 131.5, 131.1 (2C), 130.7, 130.6, 130.5, 129.9, 129.4 (2C), 128.6, 128.5, 128.3 (2C), 128.0 (2C), 113.9, 113.8, 107.5, 40.6, 27.3, 21.8, 21.7, 14.9 ppm. HRMS (ESI): calculated for C_28_H_24_N_4_NaO_3_S^+^ [M + Na]^+^, 519.1461; found 519.1461.

*N*-(5-cyano-1-(4-methoxyphenyl)-3-methyl-4-phenyl-1,4-dihydropyrano[2,3-c]-pyrazol-6-yl)-4-methylbenzenesulfonamide (4af): Synthesized with **3w** according to the general procedure of **4a**. Yield: 70%, white power solid, m. p. 69.0–70.4°C. ^1^H NMR (400 MHz, CDCl_3_) *δ* 7.50–7.42 (m, 4H), 7.41–7.35 (m, 3H), 7.11–7.03 (m, 4H), 6.70 (dt, *J* = 8.9,3.4 Hz, 2H), 4.96 (d, *J* = 9.7 Hz, 1H), 4.81 (d, *J* = 9.7 Hz, 1H), 3.81 (s, 3H), 2.40 (s, 3H), 2.14 (s, 3H) ppm; ^13^C NMR (100 MHz, CDCl_3_) *δ* 147.7, 147.0, 140.5, 137.8, 136.5, 134.7, 130.7 (2C), 130.3, 129.9 (2C), 129.5 (2C), 128.6, 128.6 (2C), 127.9 (2C), 123.3 (2C), 114.0, 113.9, 108.1, 40.5, 27.4, 21.8, 21.1, 14.9 ppm. HRMS (ESI): calculated for C_28_H_24_N_4_NaO_4_S^+^ [M + Na]^+^, 535.1410; found 535.1412.

*N*-(5-cyano-1-(4-fluorophenyl)-3-methyl-4-phenyl-1,4-dihydropyrano[2,3-c]-pyrazol-6-yl)-4-methylbenzenesulfonamide (4ag): Synthesized with **3x** according to the general procedure of **4a**. Yield: 68%, white power solid, m. p. 138.6–139.3°C. ^1^H NMR (400 MHz, CDCl_3_) *δ* 7.50–7.37 (m, 7H), 7.17–7.10 (m, 4H), 6.93–6.86 (m, 2H), 4.95 (d, *J* = 9.6 Hz, 1H), 4.82 (d, *J* = 9.6 Hz, 1H), 2.41 (s, 3H), 2.14 (s, 3H) ppm; ^13^C NMR (100 MHz, CDCl_3_) *δ* 161.7 (1C), 148.2, 147.1, 134.0, 135.3, 133.3 (1C), 130.6, 129.9 (2C), 129.2 (2C), 128.7, 128.4 (2C), 127.8 (2C), 125.5 (2C), 115.7 (2C), 112.3, 112.2, 107.7, 42.0, 27.2, 21.7, 14.3 ppm. HRMS (ESI): calculated for C_27_H_21_FN_4_NaO_3_S^+^ [M + Na]^+^, 523.1211; found 523.1210.

## Data Availability

The original contributions presented in the study are included in the article/Supplementary Material; further inquiries can be directed to the corresponding authors.
