# Supplementary material for: Design, Synthesis, and Biological Evaluation of Pyrano[2,3-c]-pyrazole–Based RalA Inhibitors Against Hepatocellular Carcinoma
Source: Front Chem. 2021 Nov 15;9:700956. doi: 10.3389/fchem.2021.700956 (PMC8634879; doi:10.3389/fchem.2021.700956)
Supplement: Supplementary file 2 [file DataSheet1.PDF]

Supplementary Material

Design, Synthesis and Biological Evaluation of Pyrano[2,3-c]-pyrazole Based RalA Inhibitors Against Hepatocellular Carcinoma

Yuting Wang[1‡], Mingyao He[2‡], Xiang Li[1,2], Jinlong Chai[2], Qinglin Jiang[3*], Cheng Peng[2], Gu He[2*], Wei Huang[1*]

[1]State Key Laboratory of Southwestern Chinese Medicine Resources, School of Pharmacy, Chengdu University of Traditional Chinese Medicine, Chengdu 611137, China

[2]State Key Laboratory of Biotherapy and Department of Urology, West China Hospital, Sichuan University, Chengdu 610041, China

[3]School of Pharmacy and Sichuan Province College Key Laboratory of Structure-Specific Small Molecule Drugs, Chengdu Medical College, Chengdu 610500, China

‡ These authors contribute equally.

*** Correspondence:**

Qinglin Jiang jql_cmc@163.com
Gu He hegu@scu.edu.cn
Wei Huang huangwei@cdutcm.edu.cn

Table of Contents

1. **Autophagy assay**.......... **S2**
2. **Western blotting analysis** **S2**
3. **Immunohistochemistry and immunofluorescent assays**.......... **S2**
4. **NMR and MS spectra** **S3**

1. Autophagy assay

The autophagy assay is relied on HepG2 cells transfected by GFP-LC3. In brief, the HepG2 cells after transfecting are processed by saline or compound 4p for 6 h, after that the cells are going to fix by paraformaldehyde and the phenomenon of autophagosome punctures can be observed through the accumulated of GFP-LC3 under laser confocal microscopy.

2. Western blotting analysis

For western blotting, the extracts of total protein from each sample were treated in accordance with the method we described previously. In short, the total proteins were loaded into the SDS-PAGE gel and separated through the different of their molecular weight in electrophoresis, after that the separated proteins were shifted into a PVDF membrane (Millipore, Burlington, MA, USA) and incubated through corresponding membrane react pathways. The resulted membranes reacted with major antibodies and HRP (horseradish peroxidase)-conjugated secondary antibodies. The expression profiles of target proteins can be identified through an enhanced chemiluminescence (ECL) substrate.

3. Immunohistochemistry and immunofluorescent assays

The histological sections of tumor tissue were immersed into EDTA antigen recovery buffer (pH 8.0) or citrate buffer (pH 6.0), and then microwave was used to recover antigen. Then the slide was incubated with the corresponding primary antibody at 37 ℃ for 30-40 min. Normal anti-rabbit or anti-mouse IgG was used as a negative control group. Immunohistochemical analysis was performed with diaminobenzidine solution after the sections was treated with HRP polymer as well as the second antibody for 30 min. Immunofluorescence (IF) test was carried out by treating slides with fluorescein combined with secondary antibody and detecting under fluorescence microscope. The IHC staining method is to use the corresponding primary antibody, as we reported in previous articles.

4. NMR and MS spectra

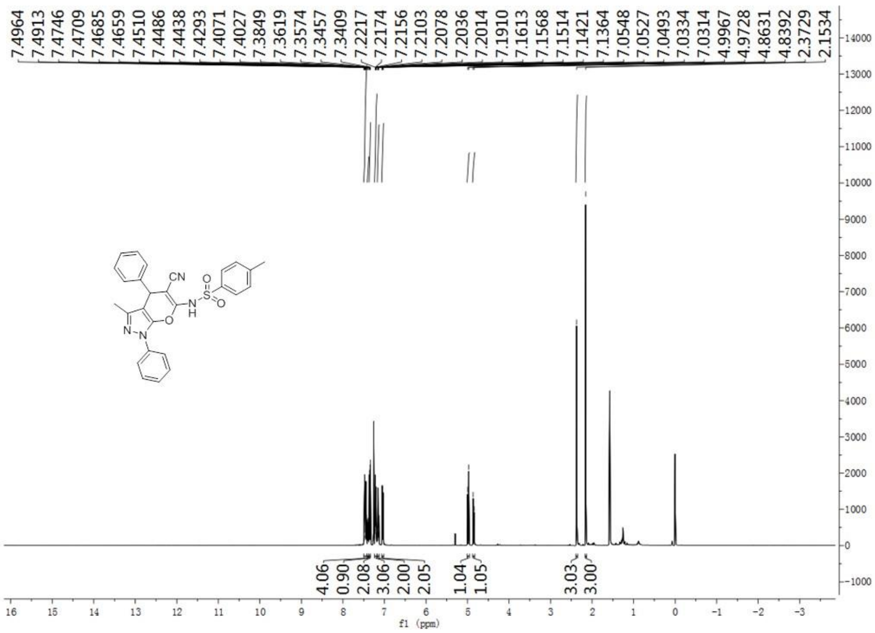

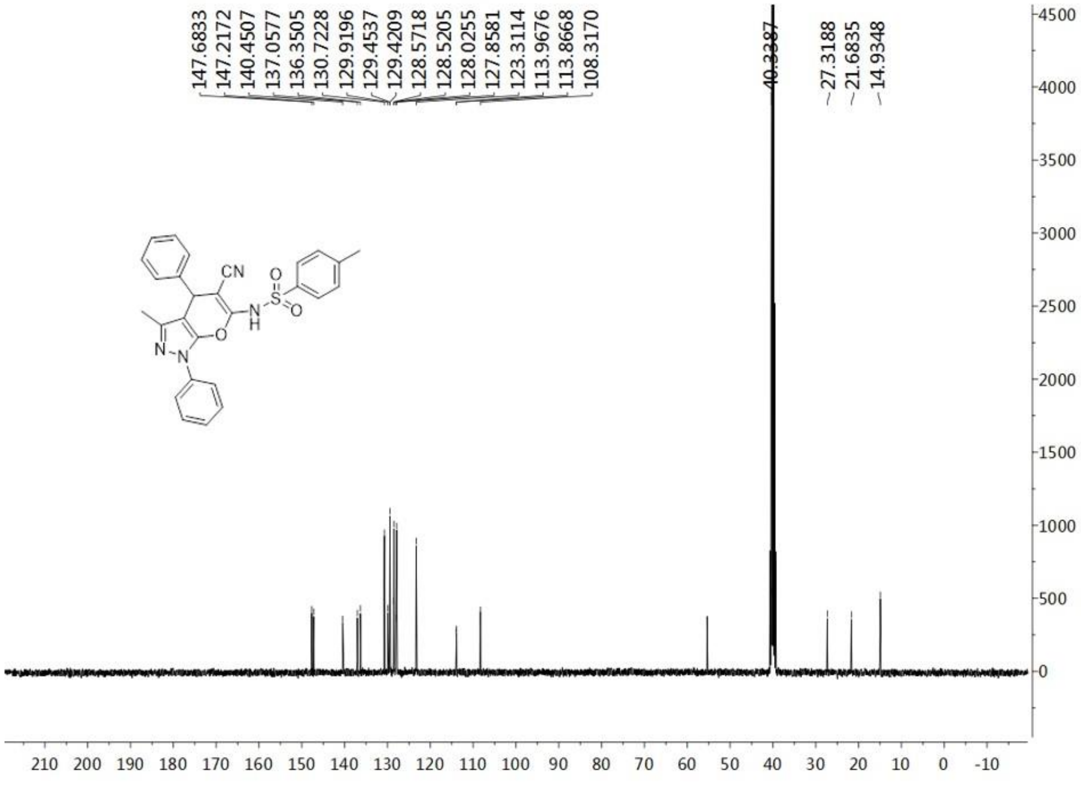

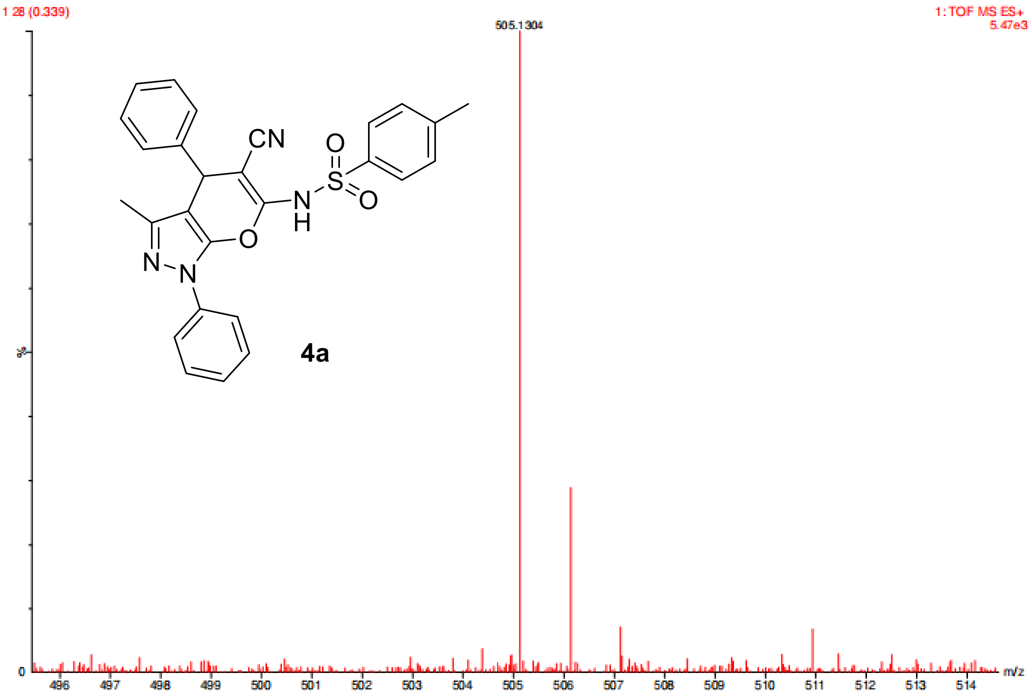

1 28 (0.339)
505.1304
1: TOF MS ES+
5.47e3
%
m/z
4a

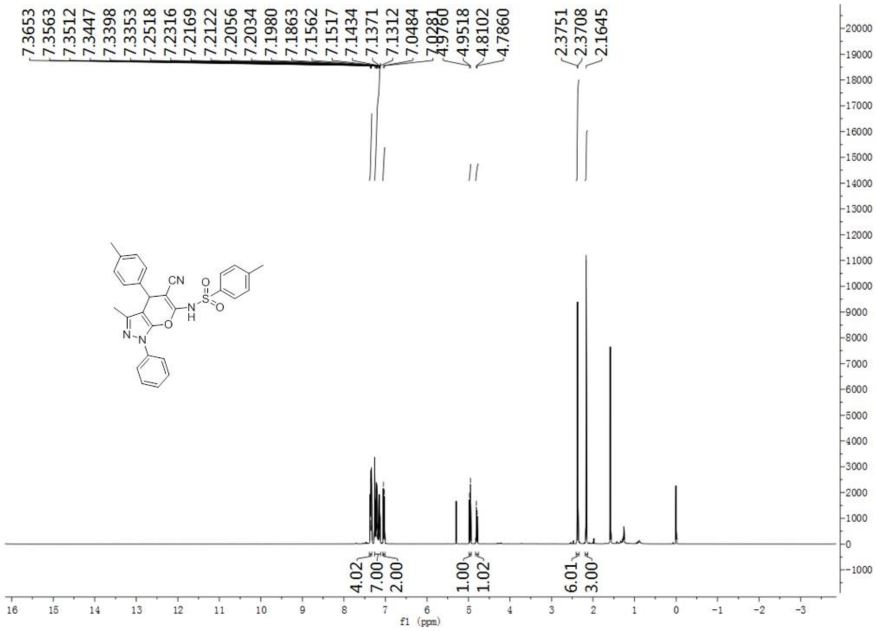

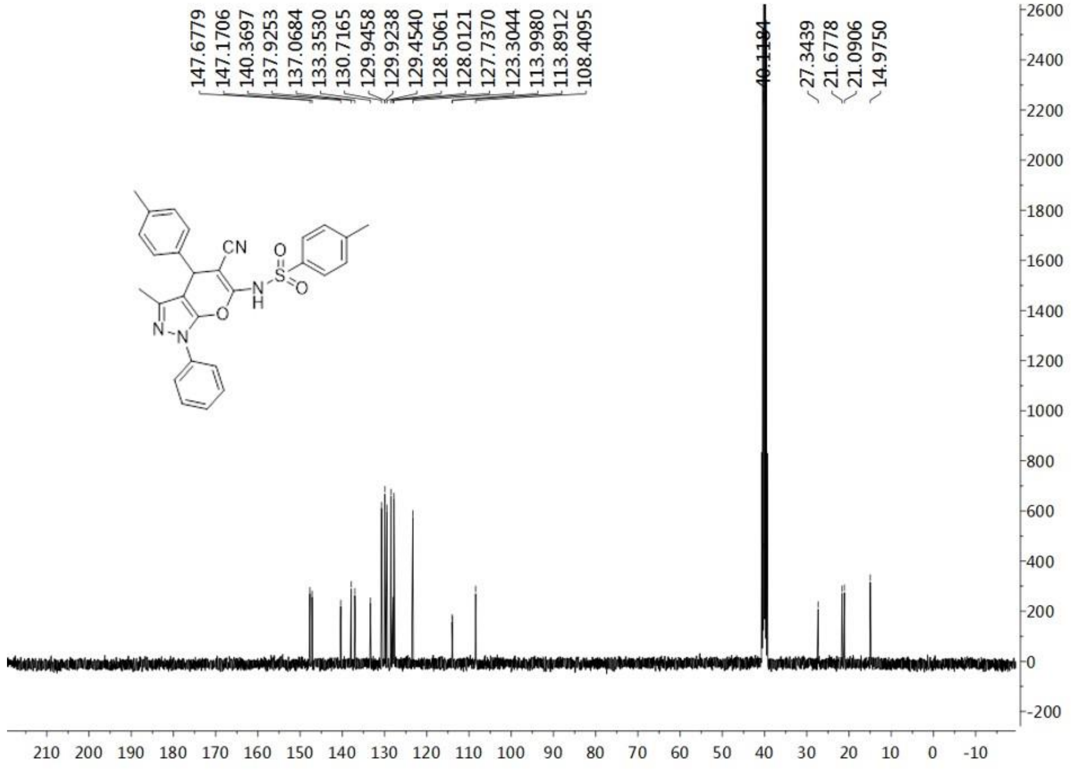
147.6779
147.1706
140.3697
137.9253
137.0684
133.3530
130.7165
129.9458
129.9238
129.4540
128.5061
128.0121
127.7370
123.3044
113.9980
113.8912
108.4095
40.1184
27.3439
21.6778
21.0906
14.9750
CN
N
H
S
O
O
N
N
O

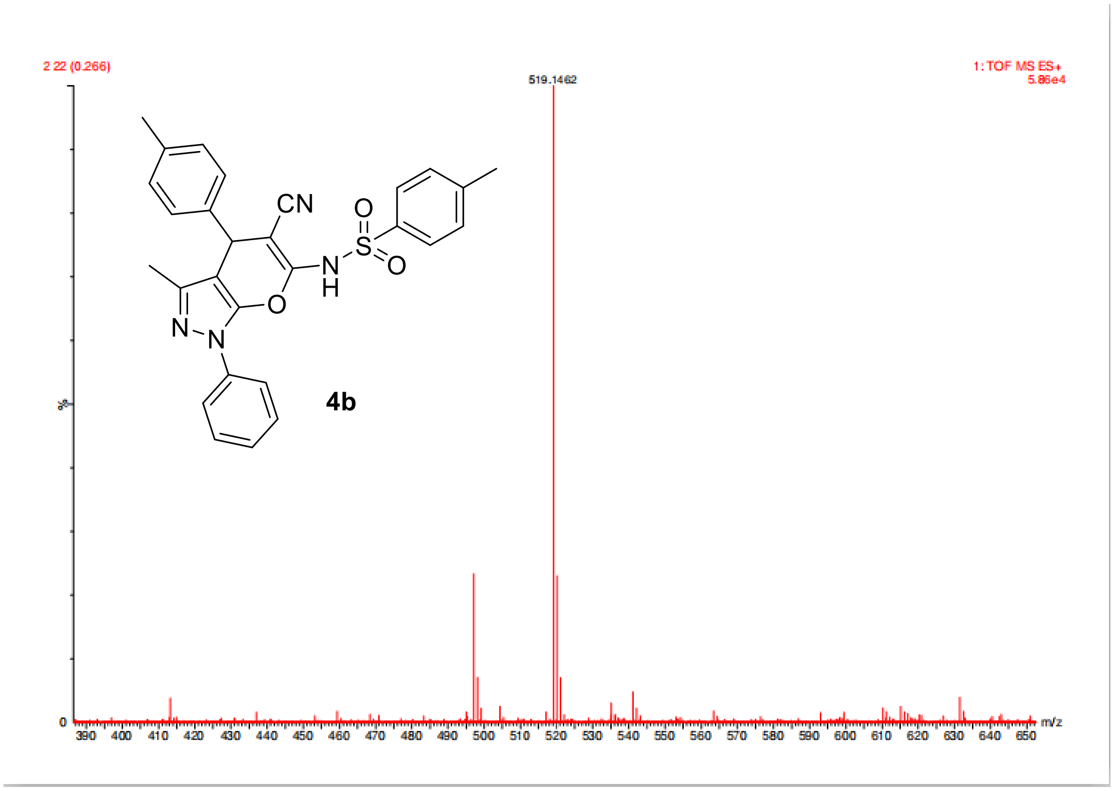
2 22 (0.266)
519.1462
1: TOF MS ES+
5.86e4
%
m/z
CN
N
H
S
O
O
N
N
O
4b

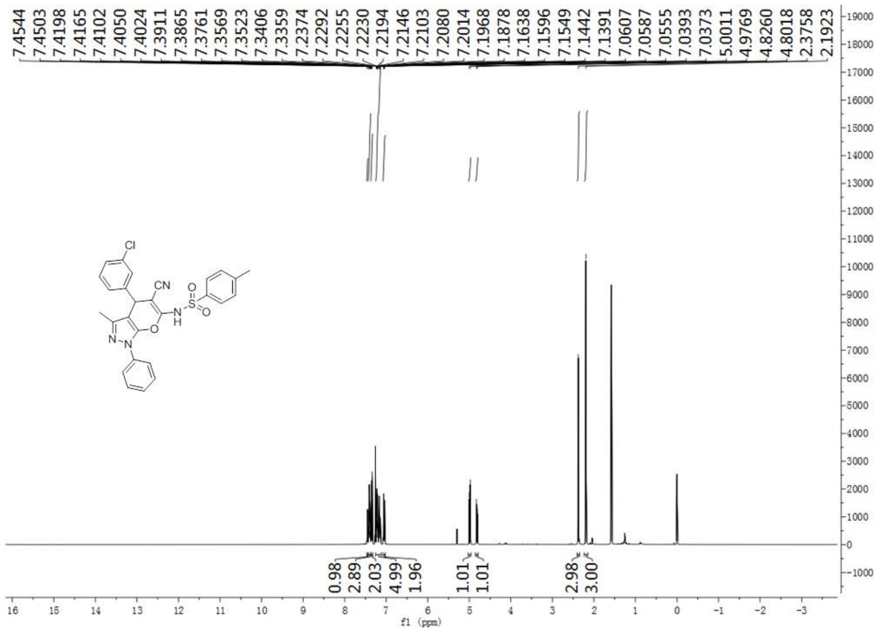

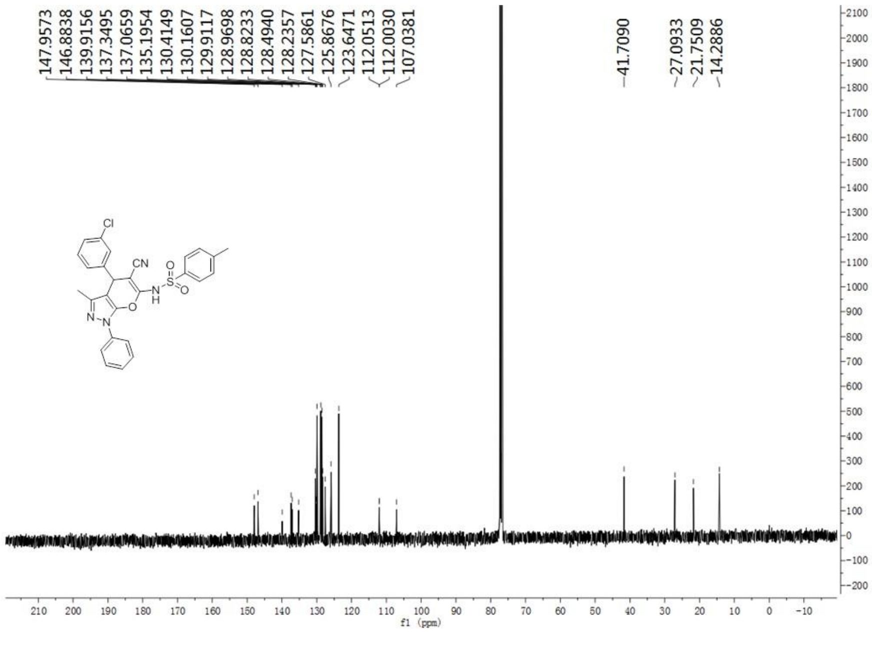

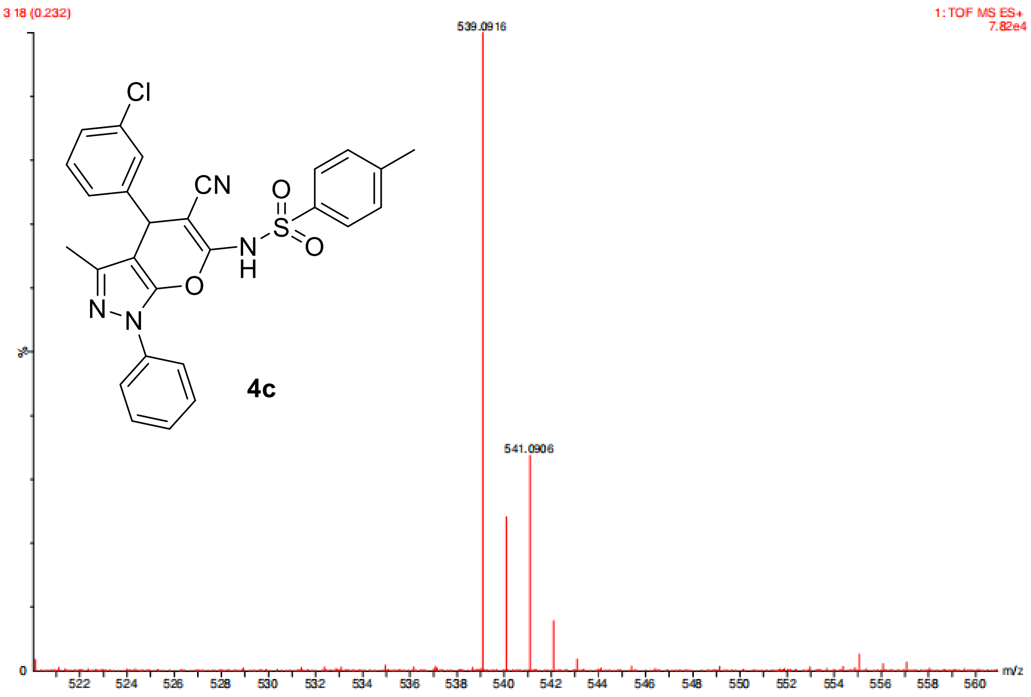
3 18 (0.232)
1: TOF MS ES+
7.82e4
539.0916
541.0906
4c
m/z
%

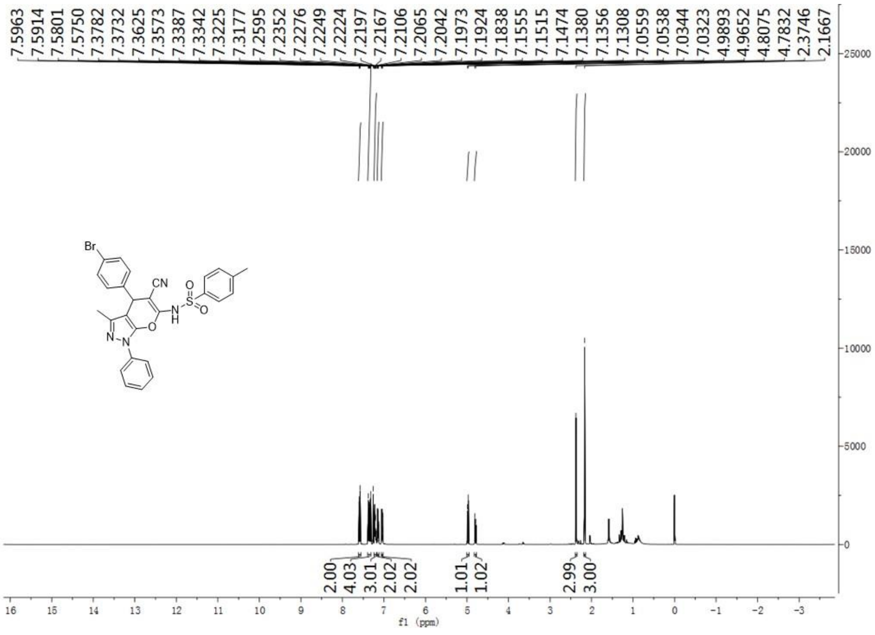

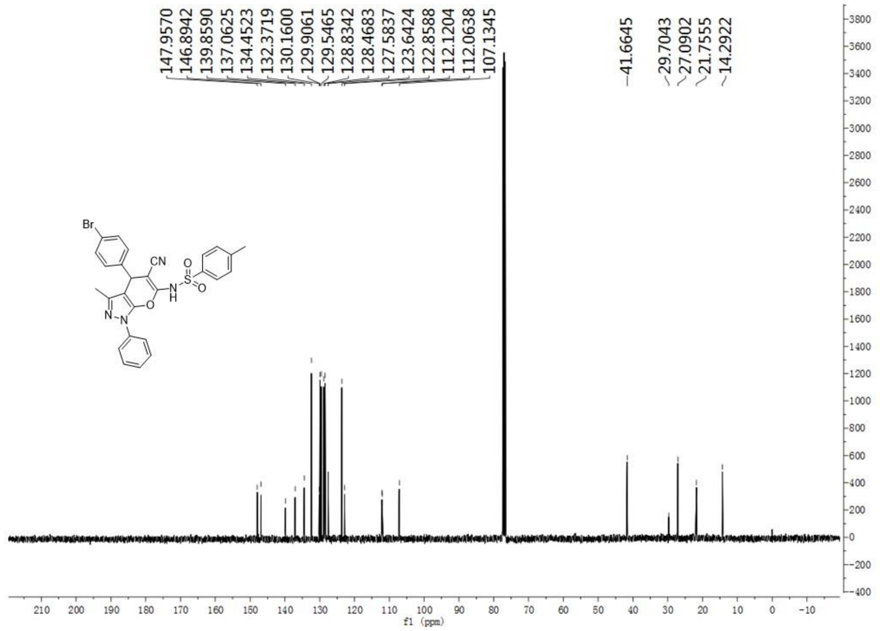
147.9570
146.8942
139.8590
137.0625
134.4523
132.3719
130.1600
129.9061
129.5465
128.8342
128.4683
127.5837
123.6424
122.8588
112.1204
112.0638
107.1345
41.6645
29.7043
27.0902
21.7555
14.2922
f1 (ppm)

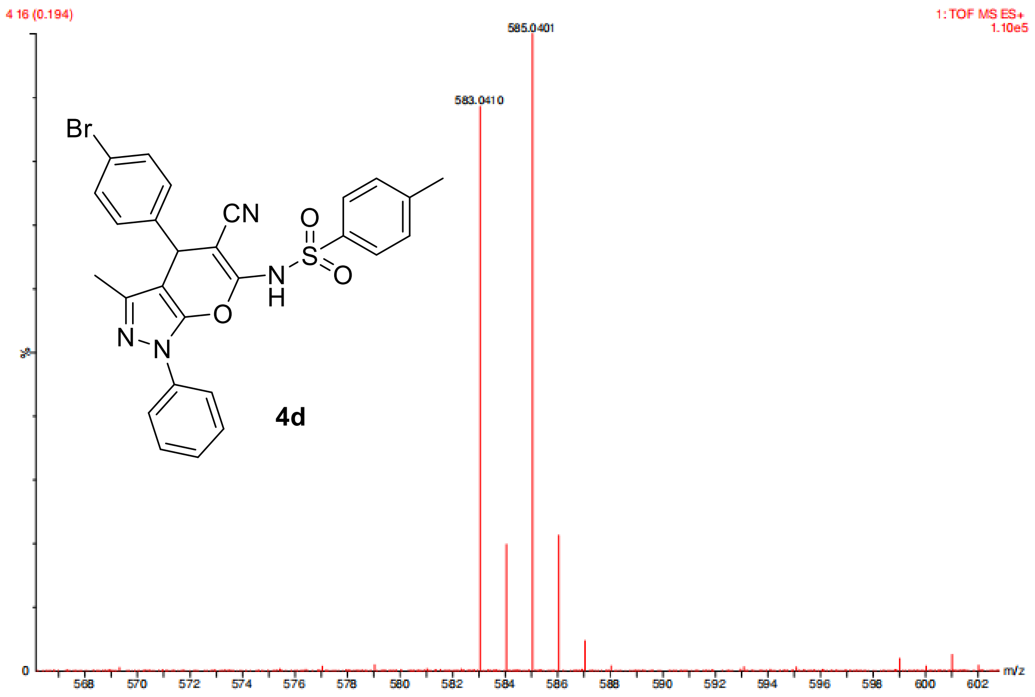
4 16 (0.194)
1: TOF MS ES+
1.10e5
585.0401
583.0410
4d
m/z

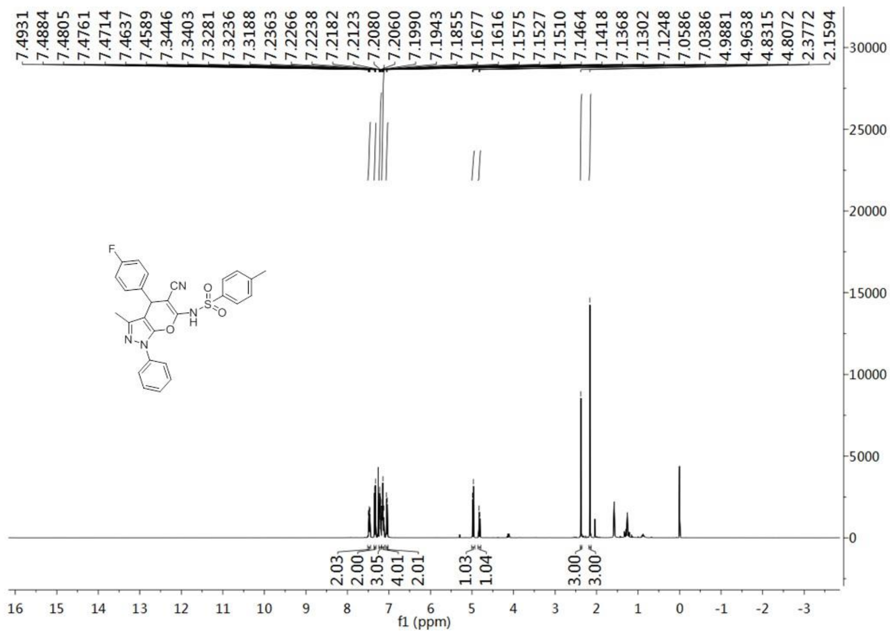

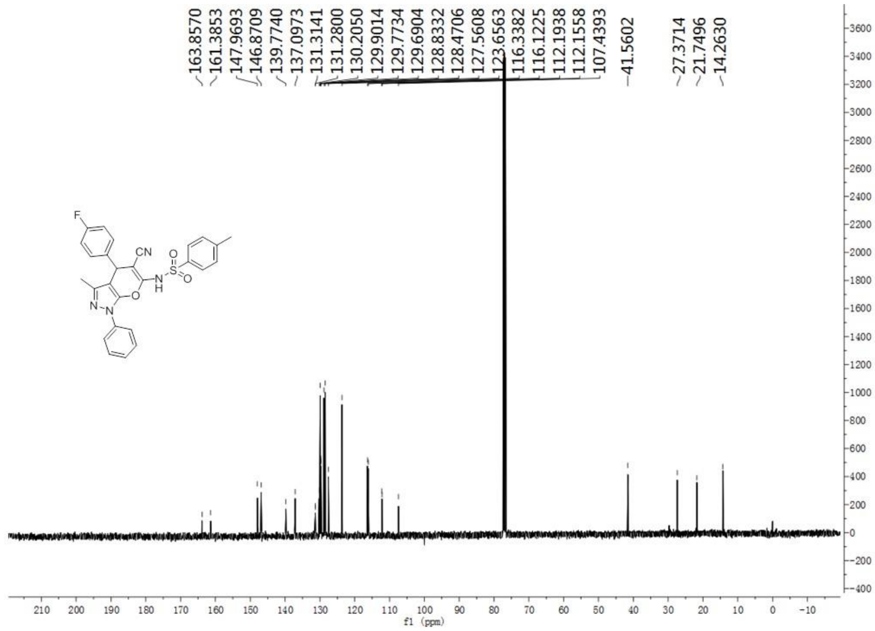

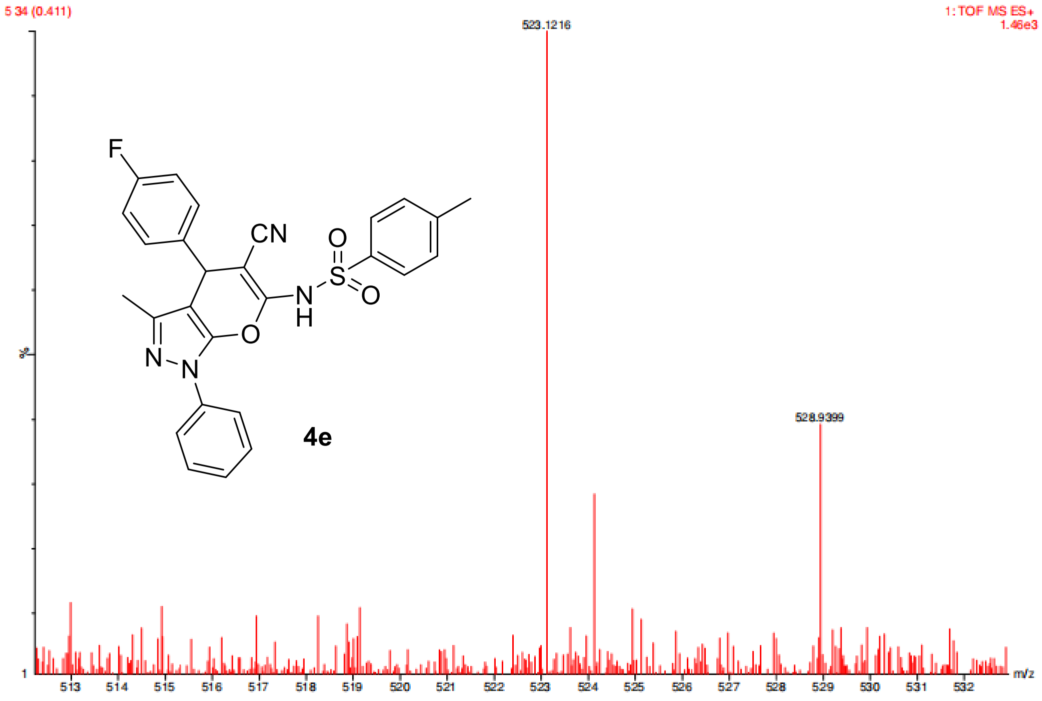

5 34 (0.411)
1: TOF MS ES+
1.46e3
523.1216
528.9399
%
4e
m/z

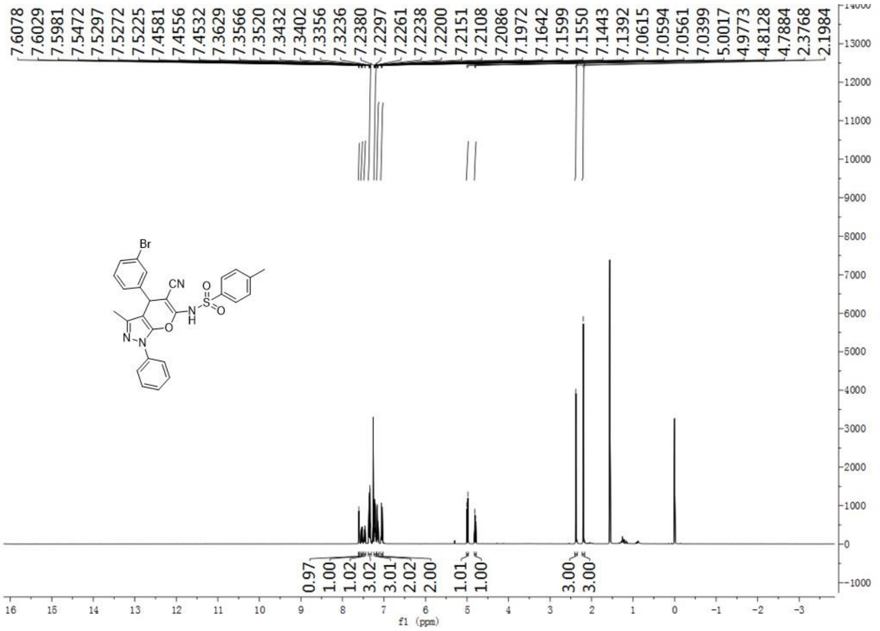

7.6078
7.6029
7.5981
7.5472
7.5297
7.5272
7.5225
7.4581
7.4556
7.4532
7.3629
7.3566
7.3520
7.3432
7.3402
7.3356
7.3236
7.2380
7.2297
7.2261
7.2238
7.2200
7.2151
7.2108
7.2086
7.1972
7.1642
7.1599
7.1550
7.1443
7.1392
7.0615
7.0594
7.0561
7.0399
5.0017
4.9773
4.8128
4.7884
2.3768
2.1984
0.97
1.00
1.02
3.02
3.01
2.02
2.00
1.01
1.00
3.00
3.00
f1 (ppm)

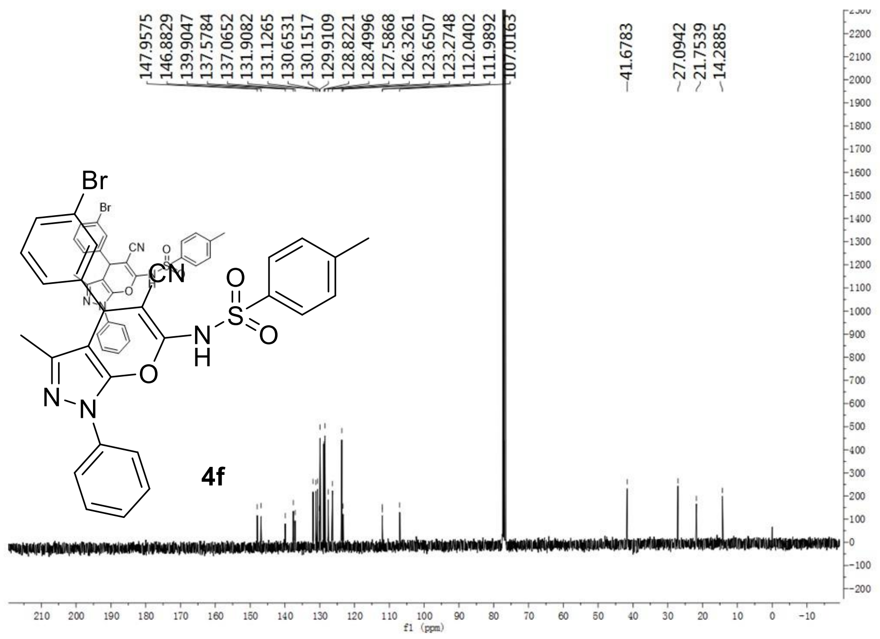
147.9575
146.8829
139.9047
137.5784
137.0652
131.9082
131.1265
130.6531
130.1517
129.9109
128.8221
128.4996
127.5868
126.3261
123.6507
123.2748
112.0402
111.9892
107.0163
41.6783
27.0942
21.7539
14.2885
Br
CN
N
H
4f
f1 (ppm)

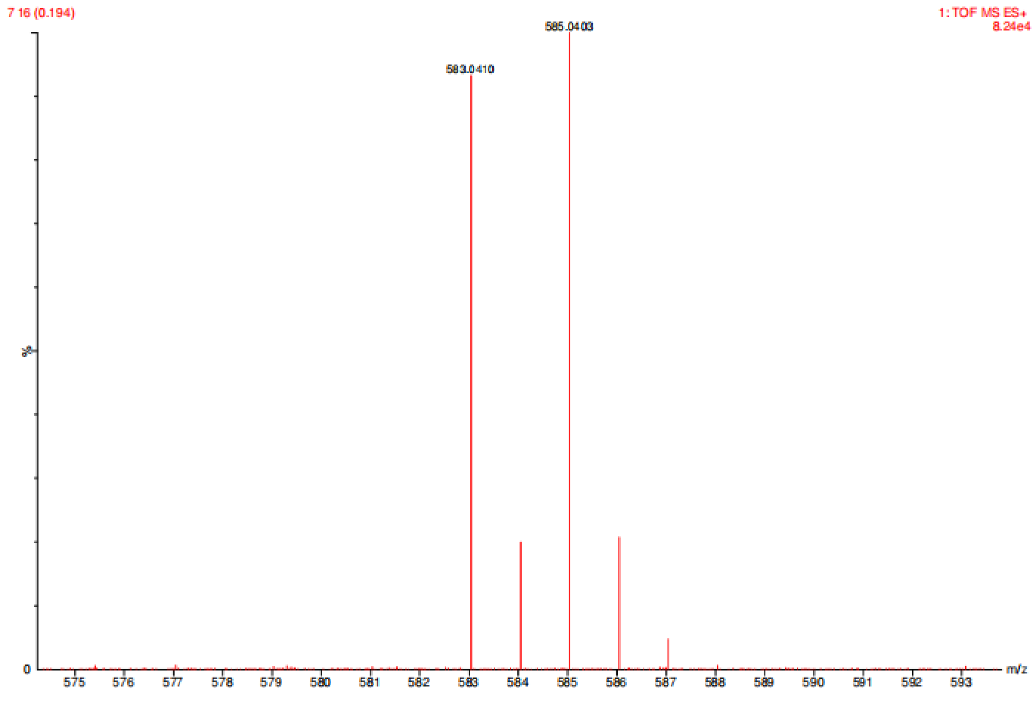
7 16 (0.194)
1: TOF MS ES+
8.24e4
585.0403
583.0410
%
m/z

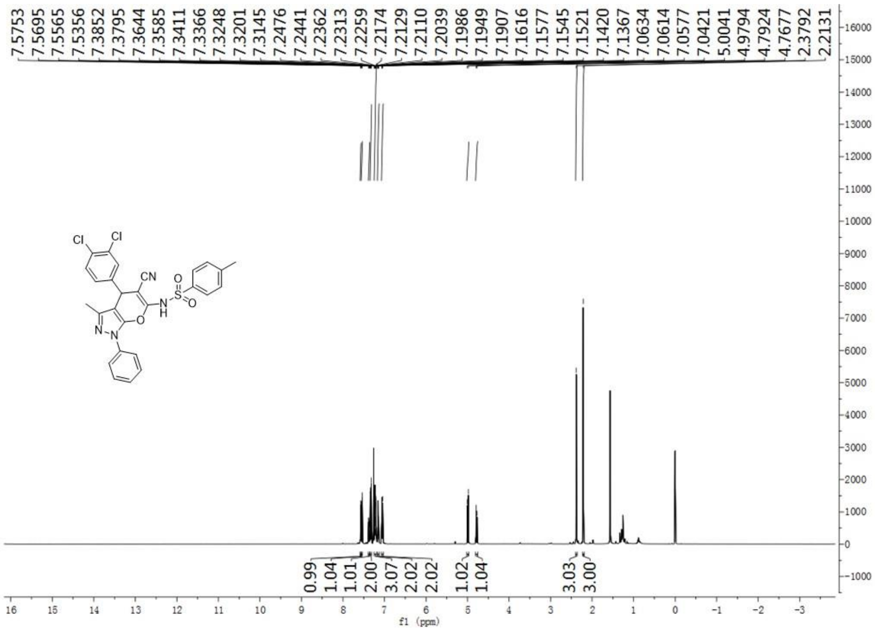
7.5753
7.5695
7.5565
7.5356
7.3852
7.3795
7.3644
7.3585
7.3411
7.3366
7.3248
7.3201
7.3145
7.2476
7.2441
7.2362
7.2313
7.2259
7.2174
7.2129
7.2110
7.2039
7.1986
7.1949
7.1907
7.1616
7.1577
7.1545
7.1521
7.1420
7.1367
7.0634
7.0614
7.0577
7.0421
5.0041
4.9794
4.7924
4.7677
2.3792
2.2131
Cl
Cl
CN
O
S
O
N
H
N
N
O
0.99
1.04
1.01
2.00
3.07
2.02
2.02
1.02
1.04
3.03
3.00
f1 (ppm)

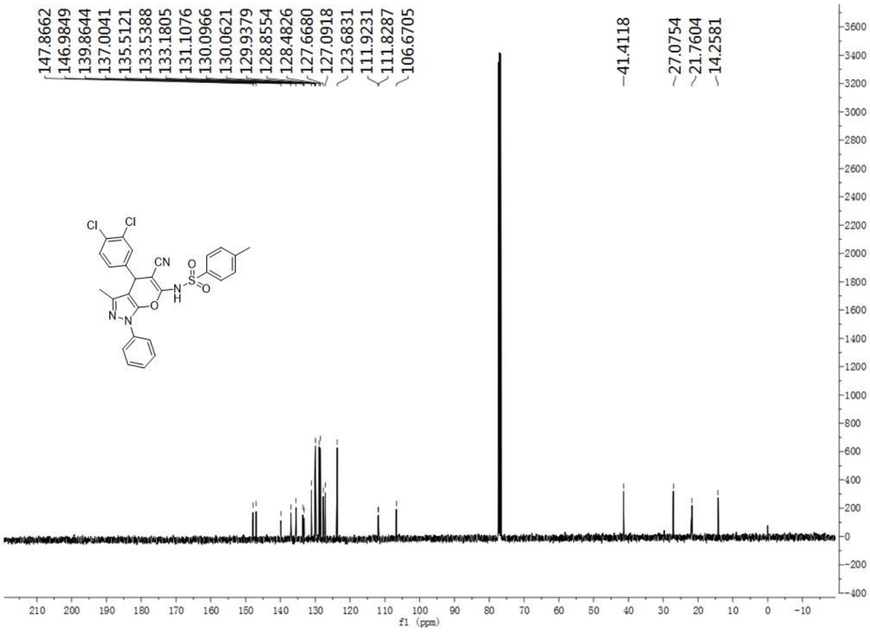
147.8662
146.9849
139.8644
137.0041
135.5121
133.5388
133.1805
131.1076
130.0966
130.0621
129.9379
128.8554
128.4826
127.6680
127.0918
123.6831
111.9231
111.8287
106.6705
41.4118
27.0754
21.7604
14.2581
Cl
Cl
CN
O
S
O
N
H
N
N
O
f1 (ppm)

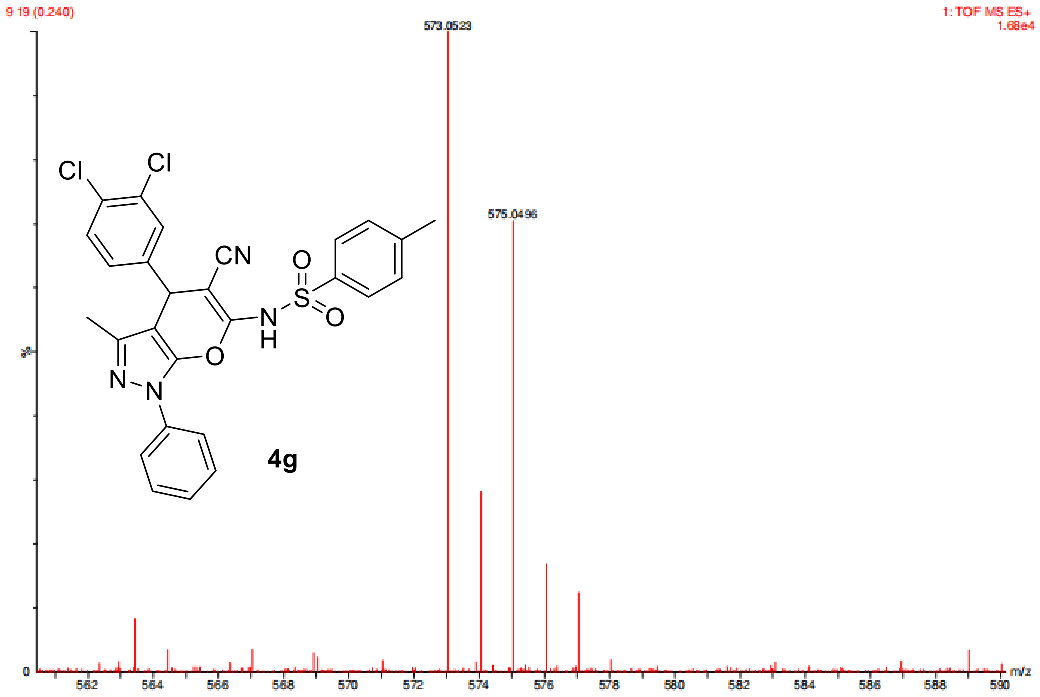

9 19 (0.240)
1: TOF MS ES+
1.68e4
573.0523
575.0496
%
m/z
4g

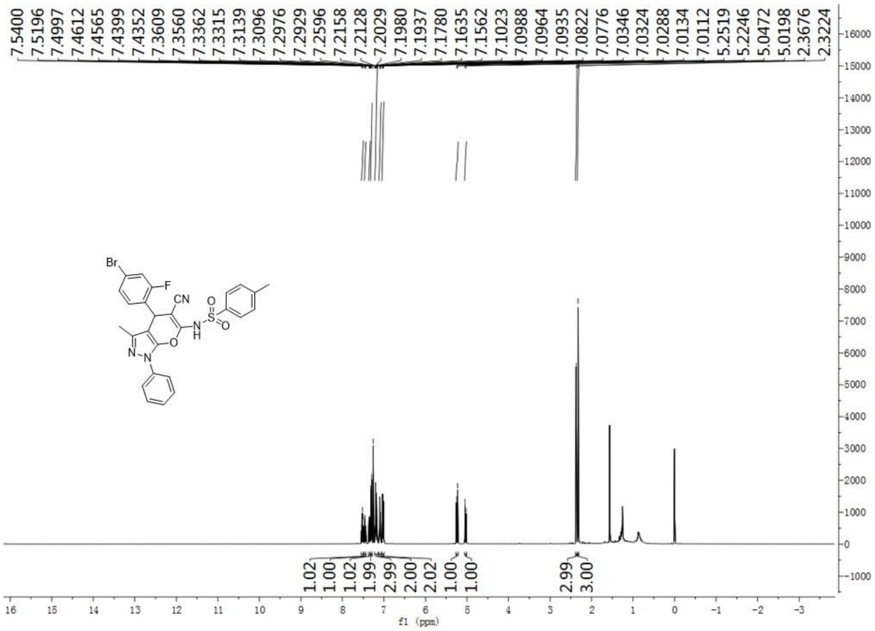

7.5400
7.5196
7.4997
7.4612
7.4565
7.4399
7.4352
7.3609
7.3560
7.3362
7.3315
7.3139
7.3096
7.2976
7.2929
7.2596
7.2158
7.2128
7.2029
7.1980
7.1937
7.1780
7.1635
7.1562
7.1023
7.0988
7.0964
7.0935
7.0822
7.0776
7.0346
7.0324
7.0288
7.0134
7.0112
5.2519
5.2246
5.0472
5.0198
2.3676
2.3224
1.02
1.00
1.02
1.99
2.99
2.00
2.02
1.00
1.00
2.99
3.00
f1 (ppm)

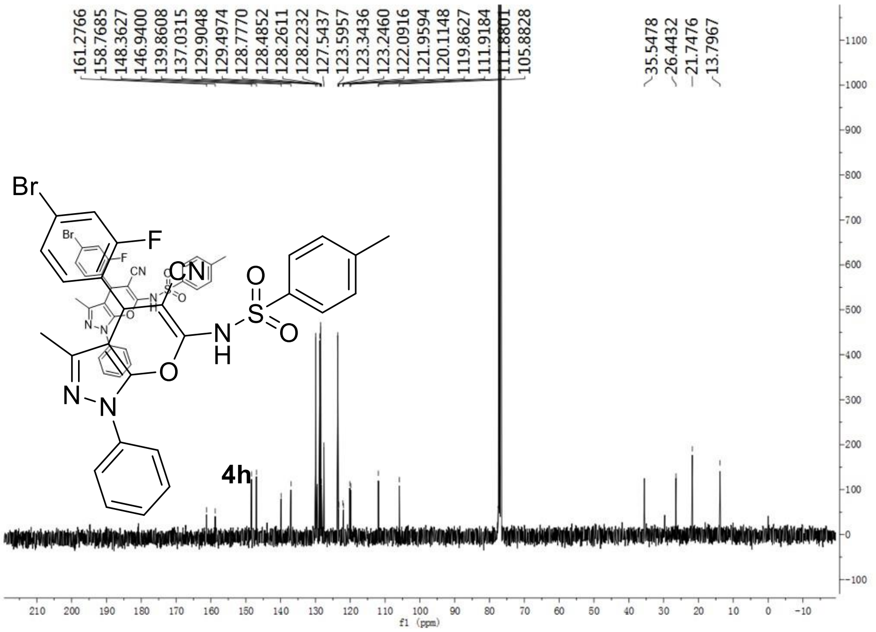
161.2766
158.7685
148.3627
146.9400
139.8608
137.0315
129.9048
129.4974
128.7770
128.4852
128.2611
128.2232
127.5437
123.5957
123.3436
123.2460
122.0916
121.9594
120.1148
119.8627
111.9184
111.8801
105.8828
35.5478
26.4432
21.7476
13.7967
Br
F
CN
O
S
O
N
H
O
N
N
4h
f1 (ppm)

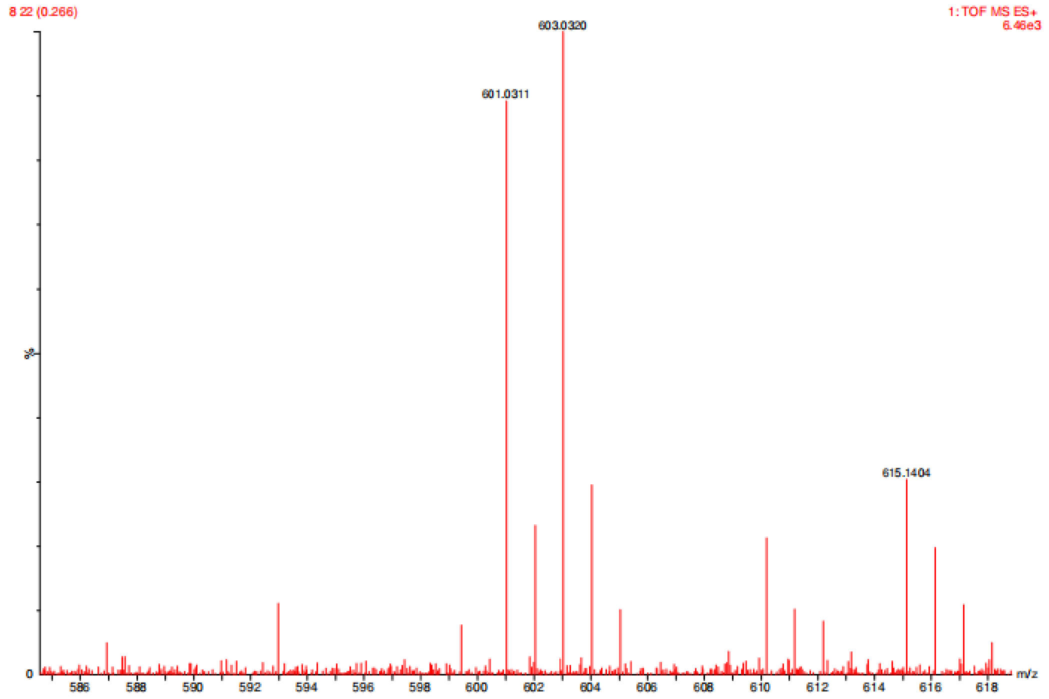
8 22 (0.266)
1: TOF MS ES+
6.46e3
603.0320
601.0311
615.1404
%
m/z

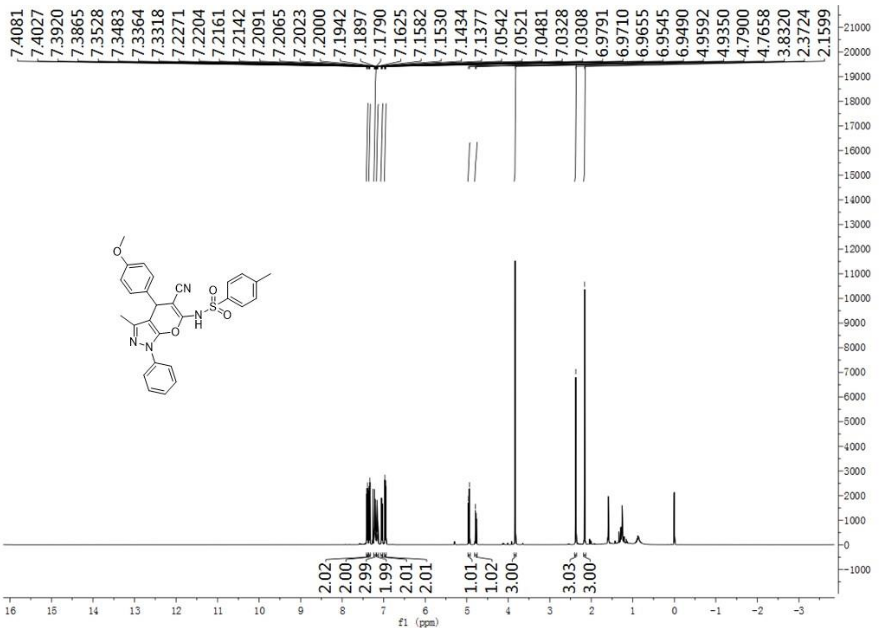
7.4081
7.4027
7.3920
7.3865
7.3528
7.3483
7.3364
7.3318
7.2271
7.2204
7.2161
7.2142
7.2091
7.2065
7.2023
7.2000
7.1942
7.1897
7.1790
7.1625
7.1582
7.1530
7.1434
7.1377
7.0542
7.0521
7.0481
7.0328
7.0308
6.9791
6.9710
6.9655
6.9545
6.9490
4.9592
4.9350
4.7900
4.7658
3.8320
2.3724
2.1599
CN
N
S
O
O
H
N
N
O
O
2.02
2.00
2.99
1.99
2.01
2.01
1.01
1.02
3.00
3.03
3.00
f1 (ppm)

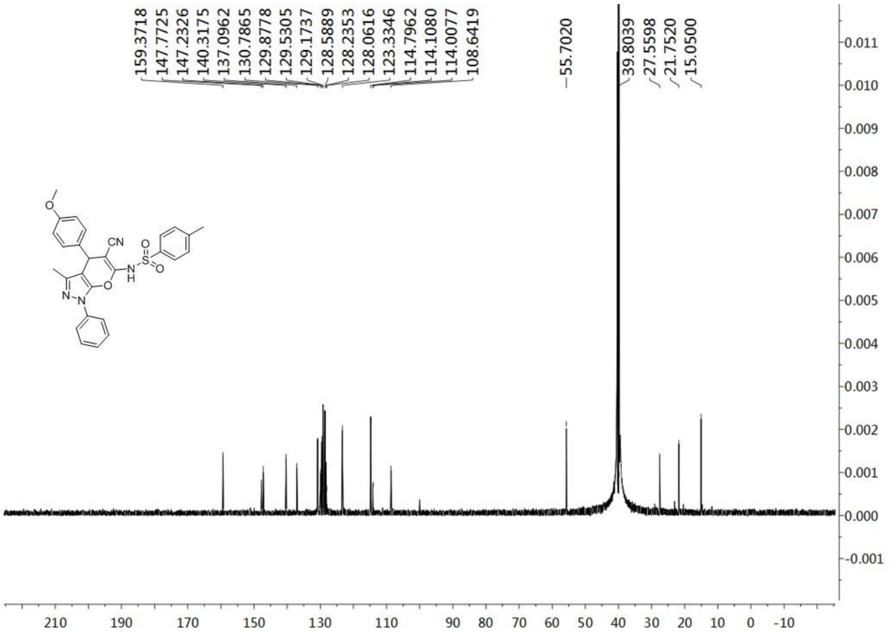
159.3718
147.7725
147.2326
140.3175
137.0962
130.7865
129.8778
129.5305
129.1737
128.5889
128.2353
128.0616
123.3346
114.7962
114.1080
114.0077
108.6419
55.7020
39.8039
27.5598
21.7520
15.0500
CN
N
S
O
O
H
N
N
O
O

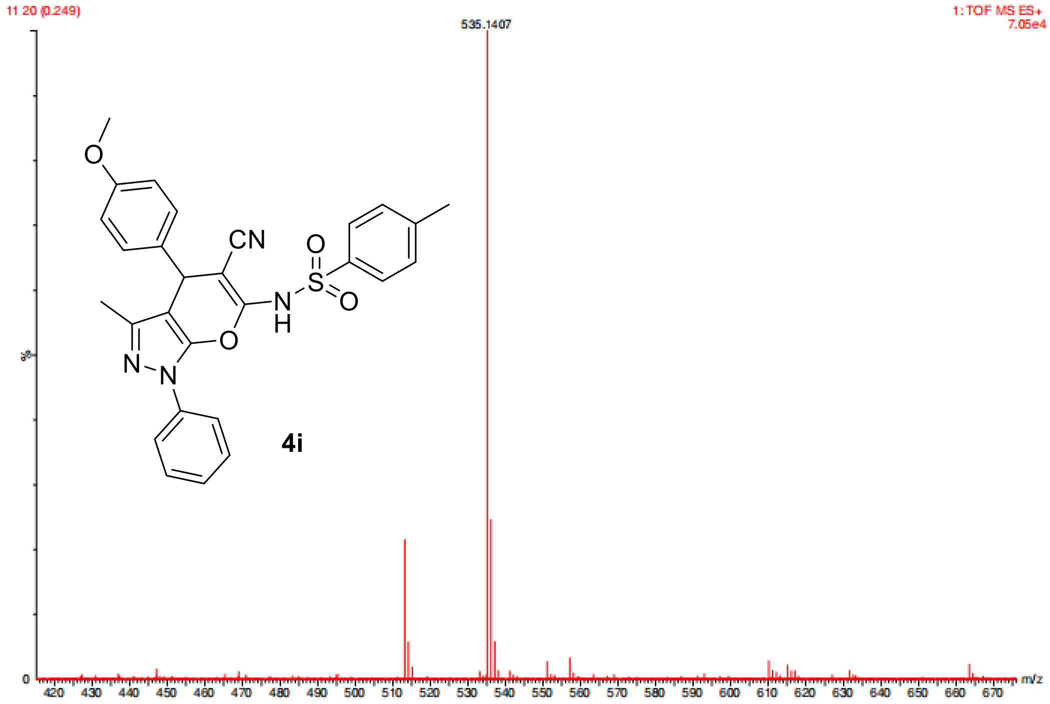

11 20 (0.249)
1: TOF MS ES+
7.05e4
535.1407
CN
O
S
O
N
H
O
O
N
N
4i
%
m/z

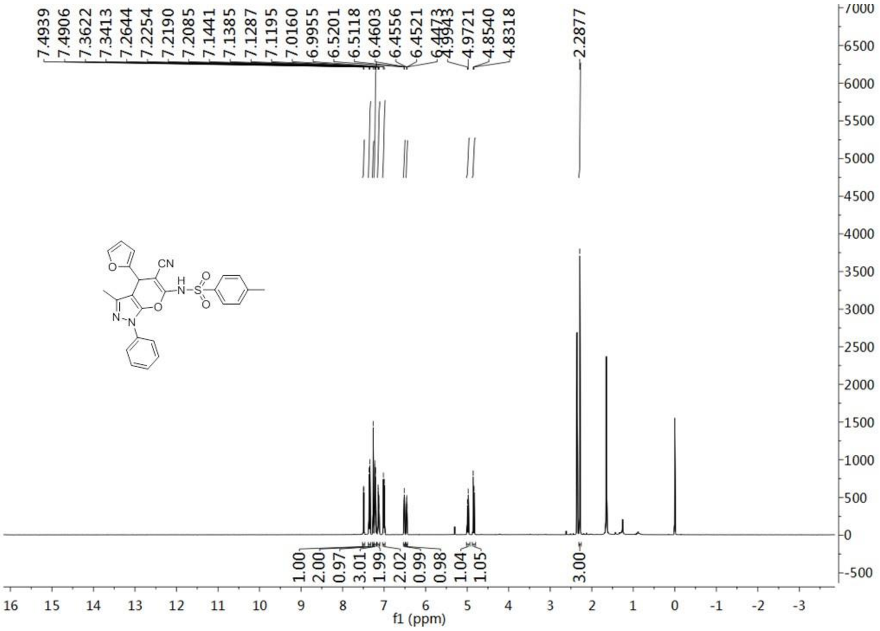

f1 (ppm)

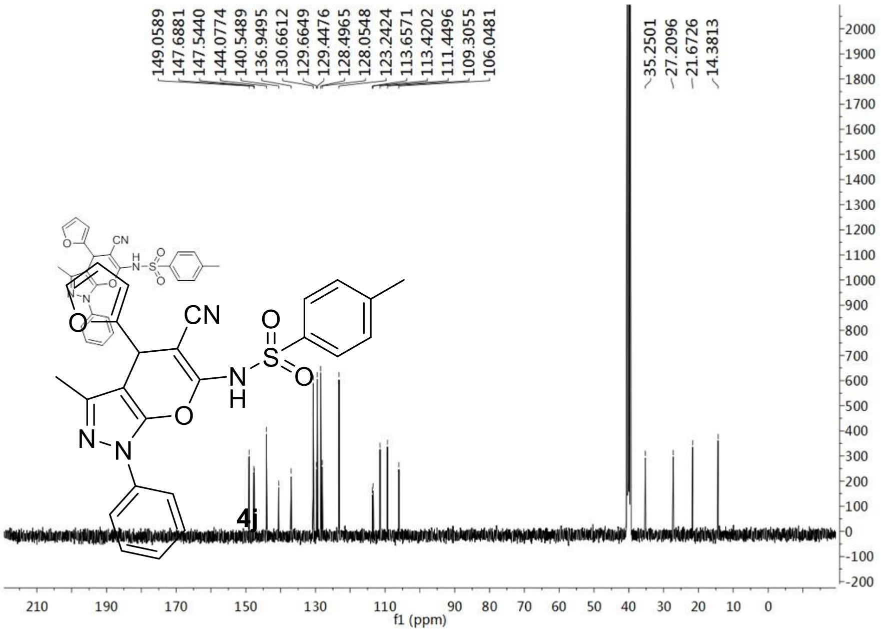
149.0589
147.6881
147.5440
144.0774
140.5489
136.9495
130.6612
129.6649
129.4476
128.4965
128.0548
123.2424
113.6571
113.4202
111.4496
109.3055
106.0481
35.2501
27.2096
21.6726
14.3813
CN
O
S
N
H
O
N
N
O
O
4i
f1 (ppm)

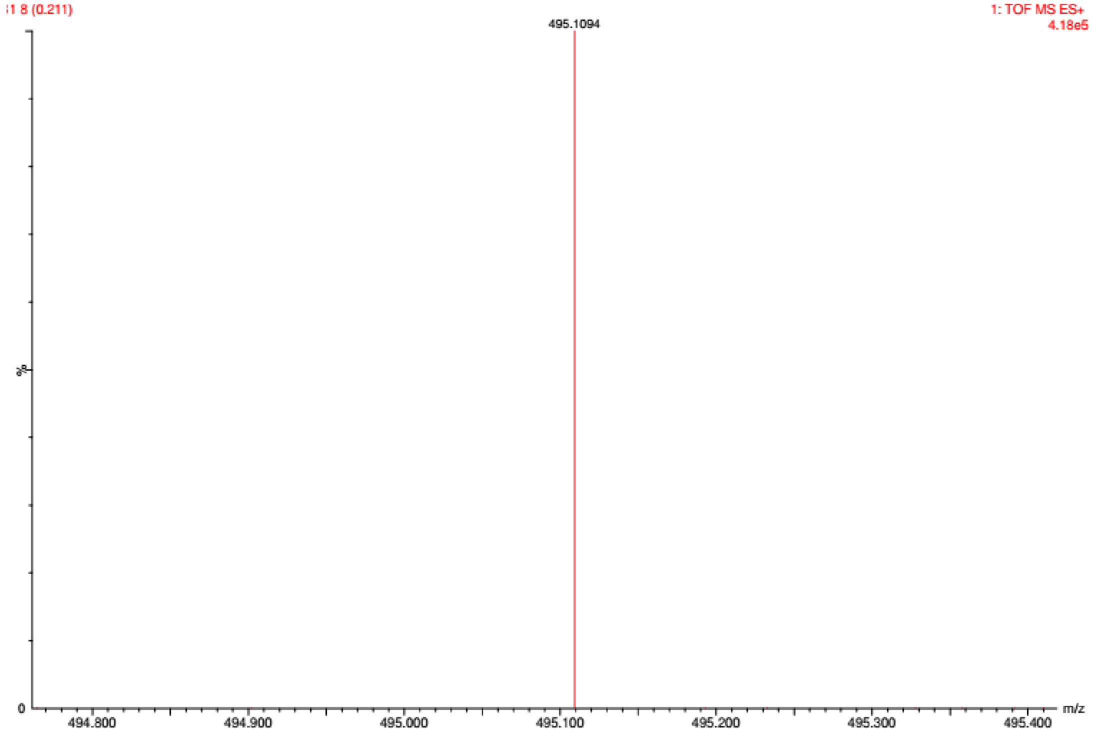
1 8 (0.211)
495.1094
1: TOF MS ES+
4.18e5
%
m/z

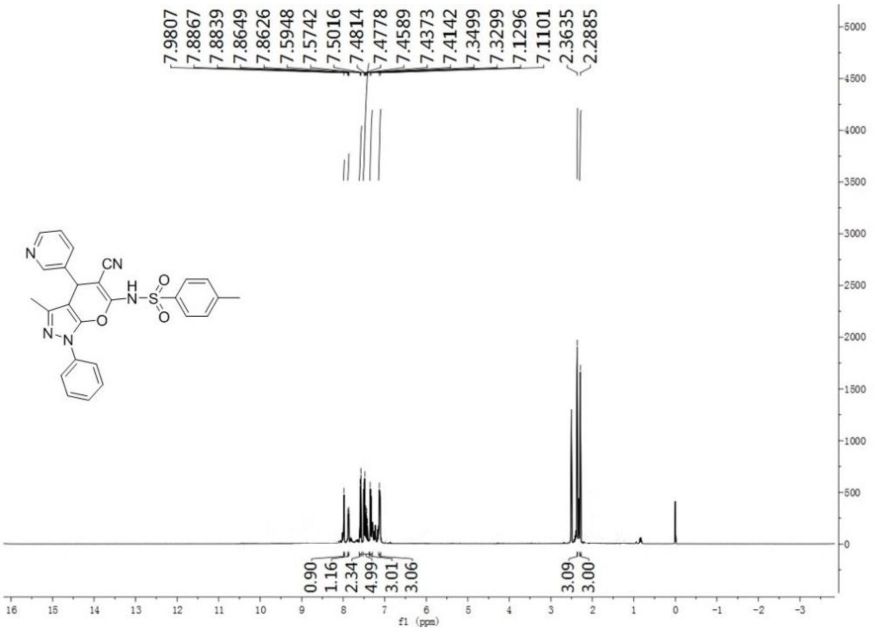
7.9807
7.8867
7.8839
7.8649
7.8626
7.5948
7.5742
7.5016
7.4814
7.4778
7.4589
7.4373
7.4142
7.3499
7.3299
7.1296
7.1101
2.3635
2.2885
0.90
1.16
2.34
4.99
3.01
3.06
3.09
3.00
f1 (ppm)

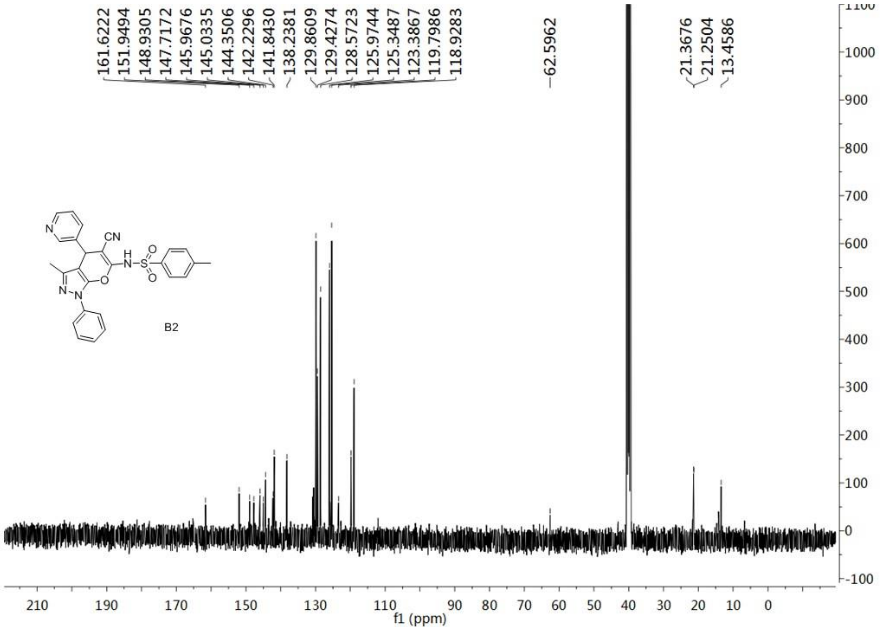
161.6222
151.9494
148.9305
147.7172
145.9676
145.0335
144.3506
142.2296
141.8430
138.2381
129.8609
129.4274
128.5723
125.9744
125.3487
123.3867
119.7986
118.9283
62.5962
21.3676
21.2504
13.4586
B2
f1 (ppm)

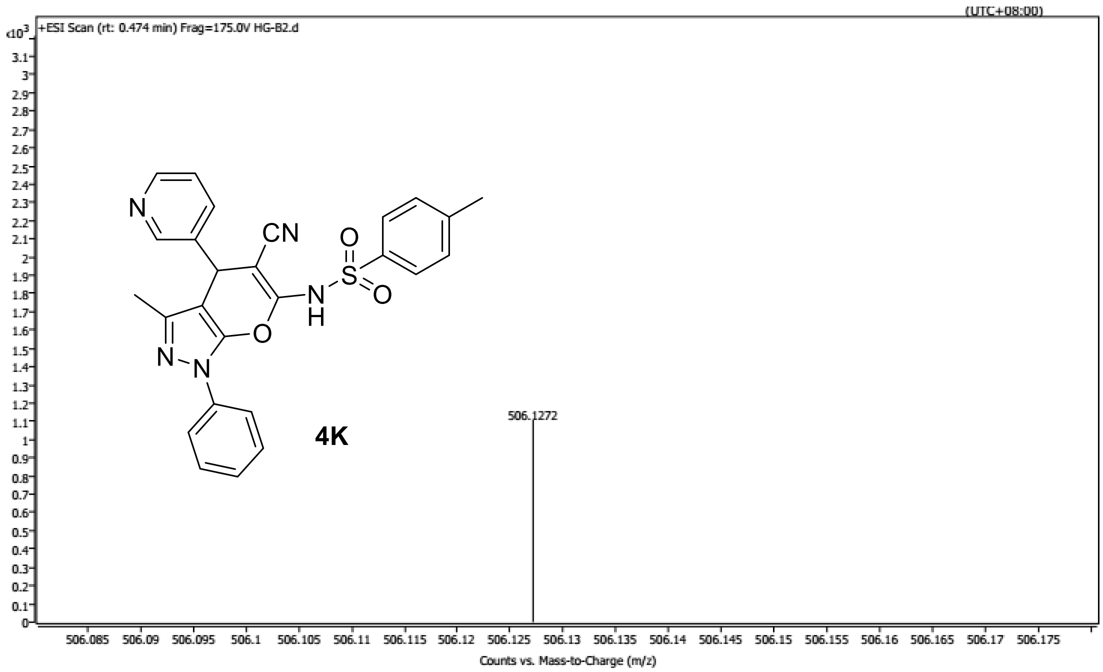
(UTC+08:00)
x10^3 +ESI Scan (rt: 0.474 min) Frag=175.0V HG-B2.d
506.1272
4K
Counts vs. Mass-to-Charge (m/z)

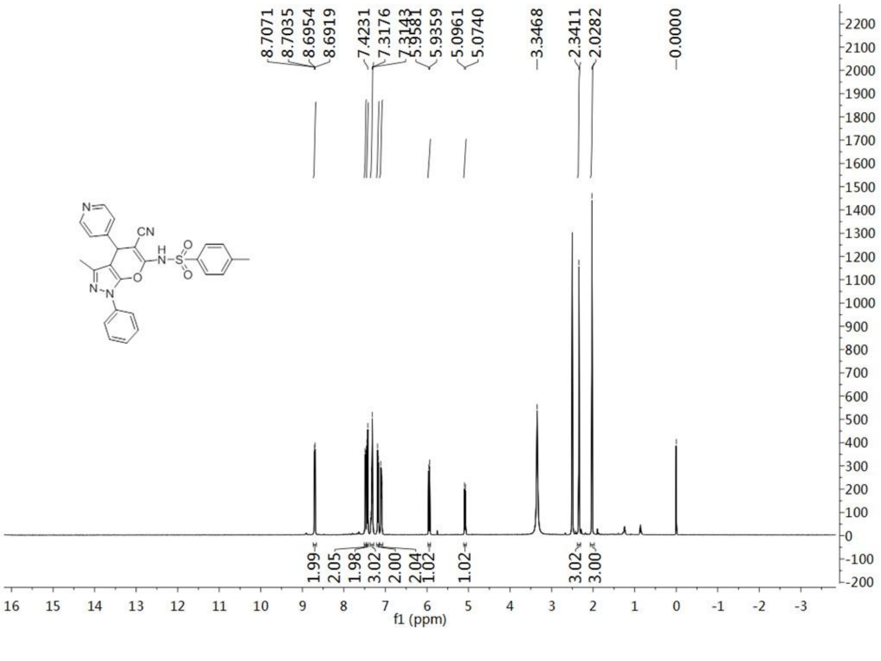
8.7071
8.7035
8.6954
8.6919
7.4231
7.3176
7.3143
5.9581
5.9359
5.0961
5.0740
3.3468
2.3411
2.0282
0.0000
1.99
2.05
1.98
3.02
2.00
2.04
1.02
1.02
3.02
3.00
f1 (ppm)

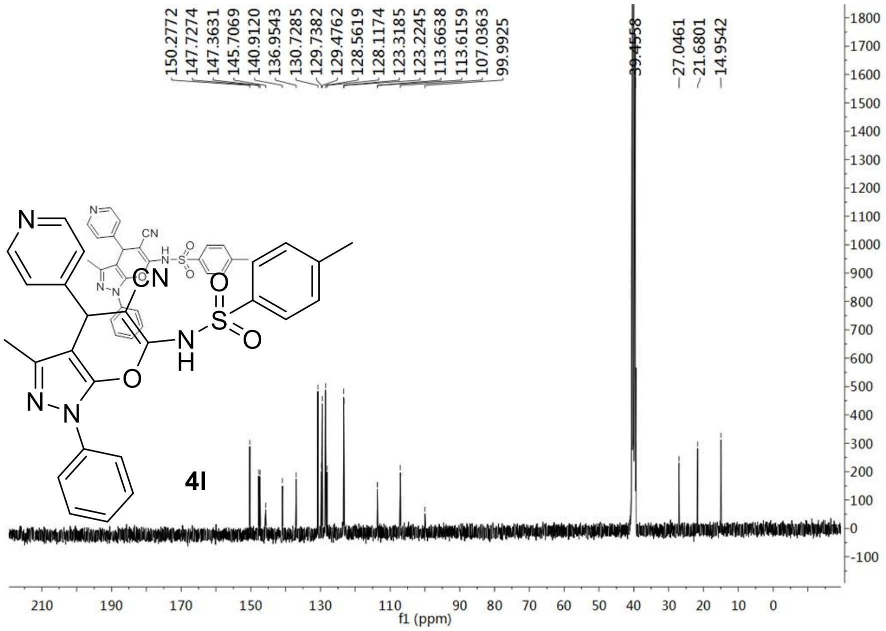
150.2772
147.7274
147.3631
145.7069
140.9120
136.9543
130.7285
129.7382
129.4762
128.5619
128.1174
123.3185
123.2245
113.6638
113.6159
107.0363
99.9925
39.4558
27.0461
21.6801
14.9542
4l
f1 (ppm)

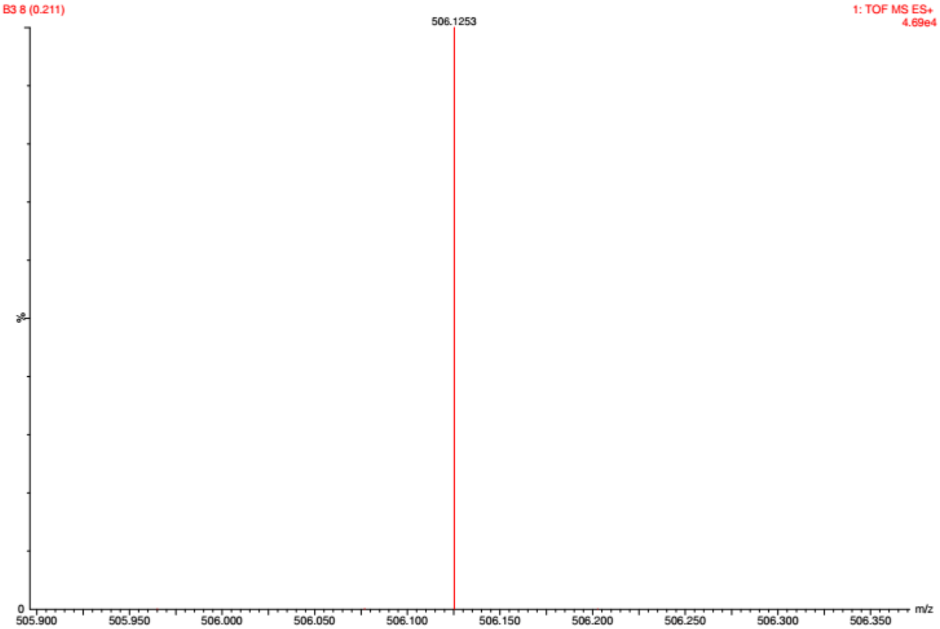
B3 8 (0.211)
506.1253
1: TOF MS ES+
4.69e4
%
m/z

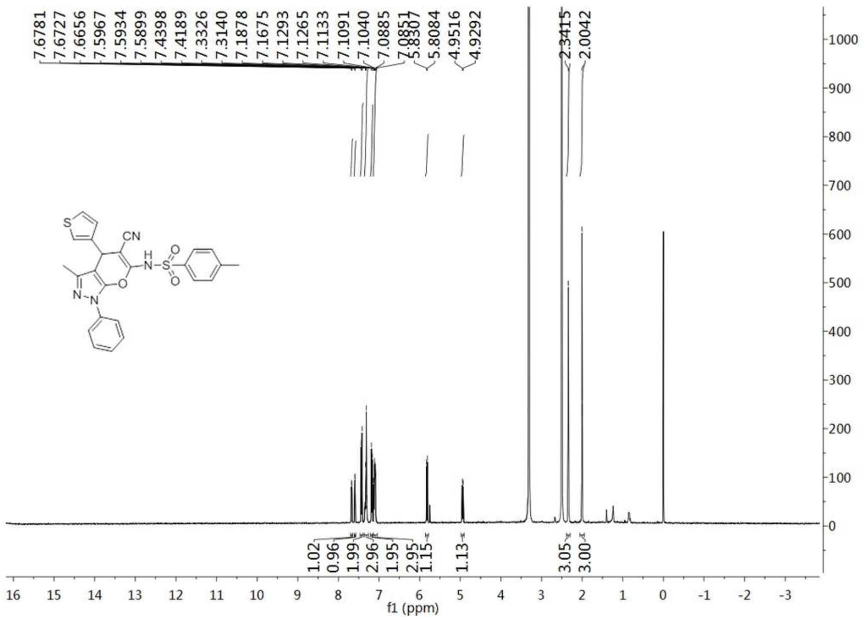
7.6781
7.6727
7.6656
7.5967
7.5934
7.5899
7.4398
7.4189
7.3326
7.3140
7.1878
7.1675
7.1293
7.1265
7.1133
7.1091
7.1040
7.0885
7.0851
5.8307
5.8084
4.9516
4.9292
2.3415
2.0042
CN
H
N
S
O
1.02
0.96
1.99
2.96
1.95
2.95
1.15
1.13
3.05
3.00
f1 (ppm)

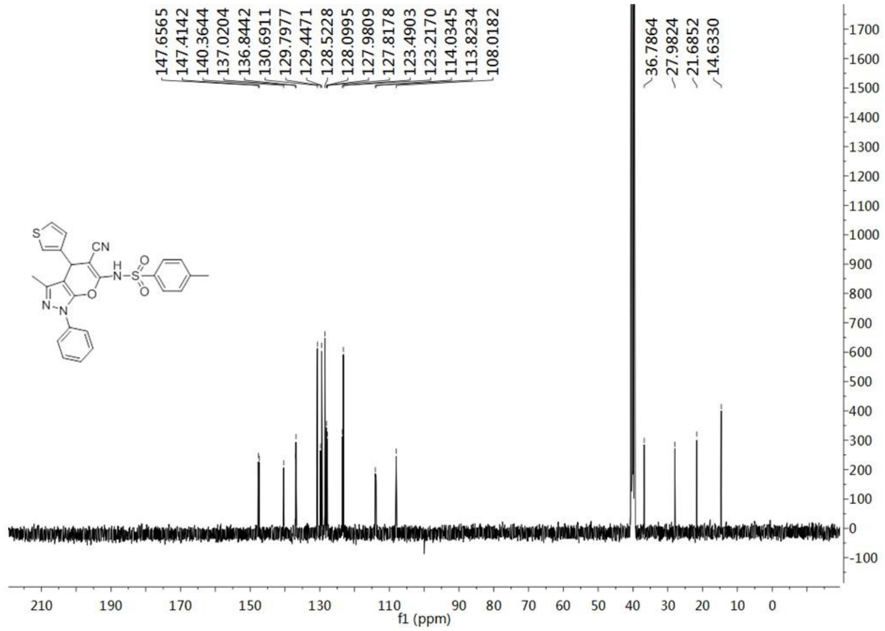
147.6565
147.4142
140.3644
137.0204
136.8442
130.6911
129.7977
129.4471
128.5228
128.0995
127.9809
127.8178
123.4903
123.2170
114.0345
113.8234
108.0182
36.7864
27.9824
21.6852
14.6330
CN
H
N
S
O
f1 (ppm)

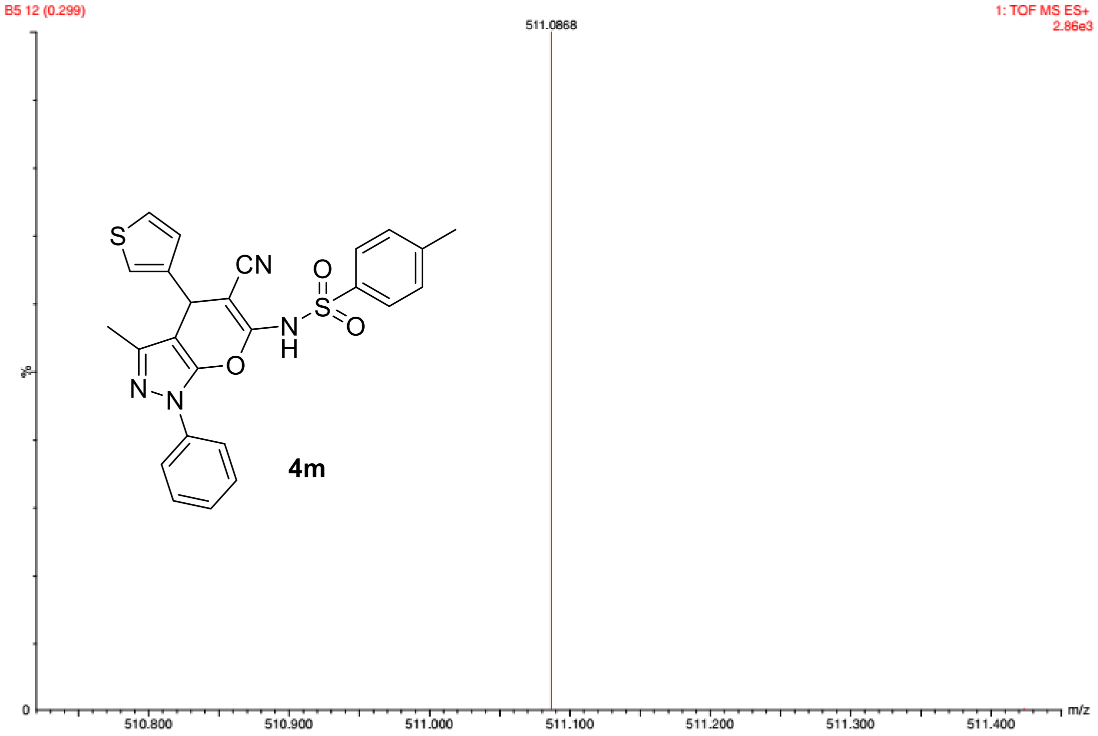
B5 12 (0.299)
511.0868
1: TOF MS ES+
2.86e3
CN
4m
%
m/z
510.800
510.900
511.000
511.100
511.200
511.300
511.400

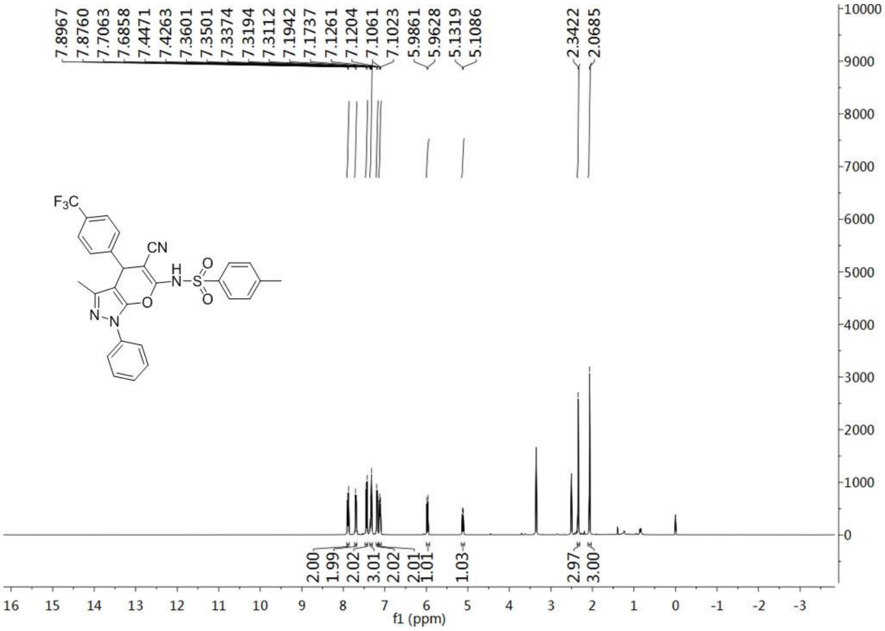
7.8967
7.8760
7.7063
7.6858
7.4471
7.4263
7.3601
7.3501
7.3374
7.3194
7.3112
7.1942
7.1737
7.1261
7.1204
7.1061
7.1023
5.9861
5.9628
5.1319
5.1086
2.3422
2.0685
F3C
CN
2.00
1.99
2.02
3.01
2.02
2.01
1.01
1.03
2.97
3.00
f1 (ppm)

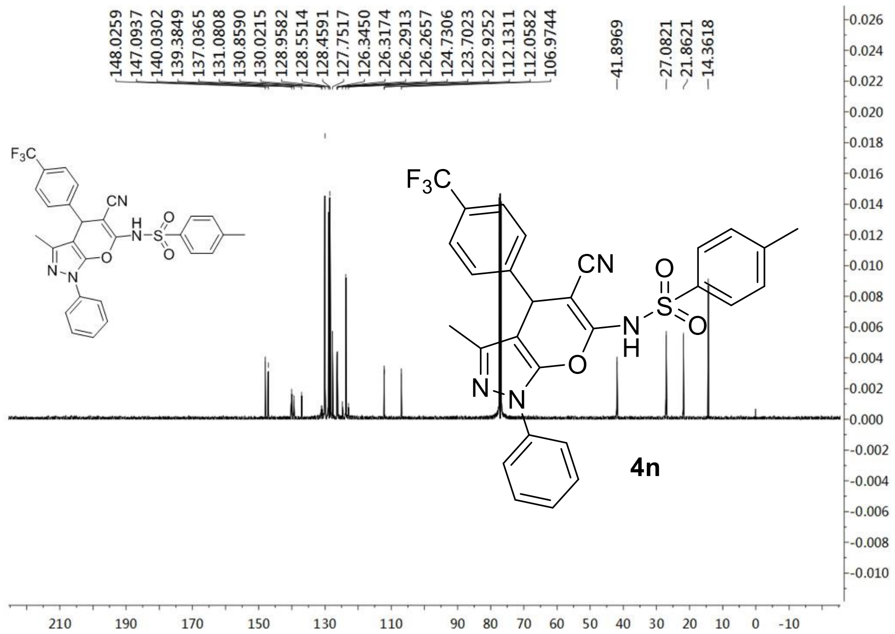
148.0259
147.0937
140.0302
139.3849
137.0365
131.0808
130.8590
130.0215
128.9582
128.5514
128.4591
127.7517
126.3450
126.3174
126.2913
126.2657
124.7306
123.7023
122.9252
112.1311
112.0582
106.9744
41.8969
27.0821
21.8621
14.3618
4n

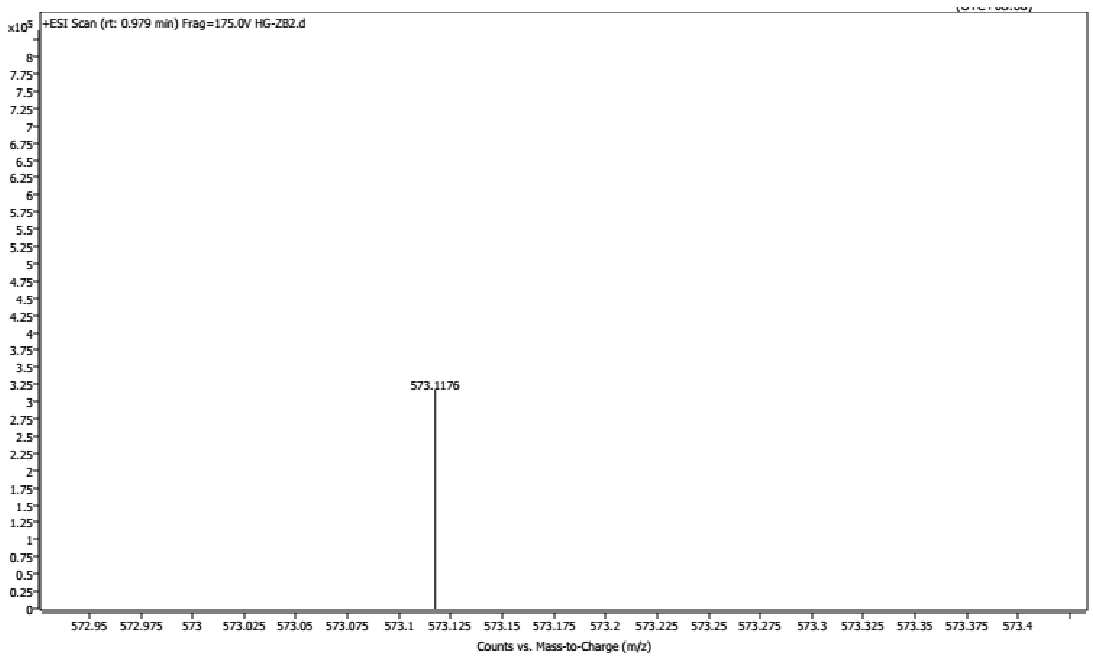
+ESI Scan (rt: 0.979 min) Frag=175.0V HG-ZB2.d
573.1176
Counts vs. Mass-to-Charge (m/z)

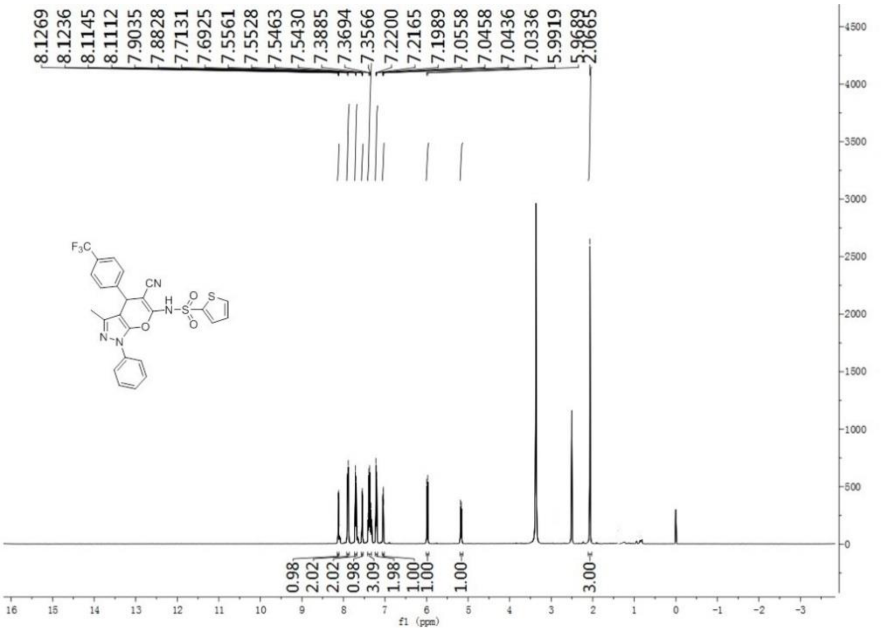
8.1269
8.1236
8.1145
8.1112
7.9035
7.8828
7.7131
7.6925
7.5561
7.5528
7.5463
7.5430
7.3885
7.3694
7.3566
7.2200
7.2165
7.1989
7.0558
7.0458
7.0436
7.0336
5.9919
5.9689
2.0665
0.98
2.02
2.02
0.98
3.09
1.98
1.00
1.00
1.00
3.00
f1 (ppm)

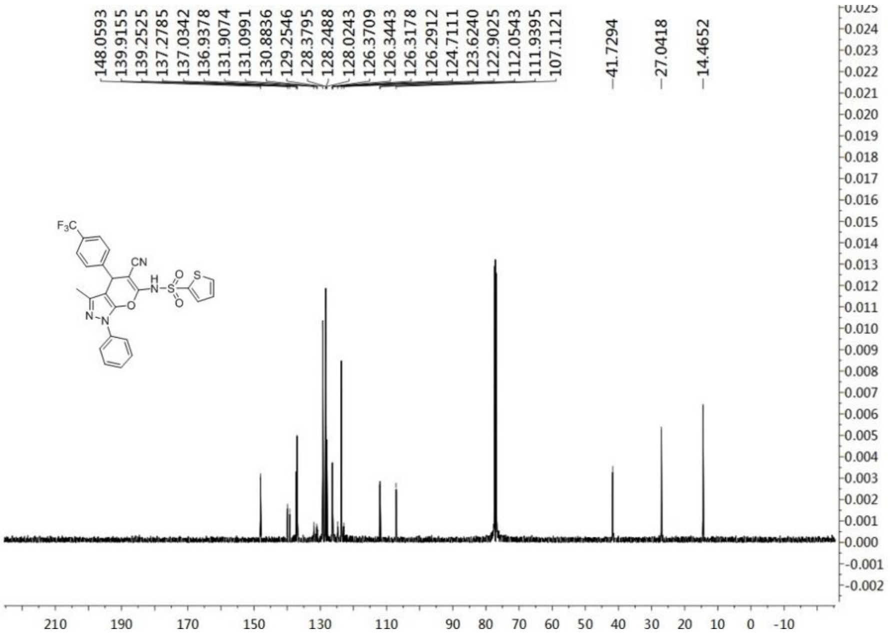
148.0593
139.9155
139.2525
137.2785
137.0342
136.9378
131.9074
131.0991
130.8836
129.2546
128.3795
128.2488
128.0243
126.3709
126.3443
126.3178
126.2912
124.7111
123.6240
122.9025
112.0543
111.9395
107.1121
41.7294
27.0418
14.4652

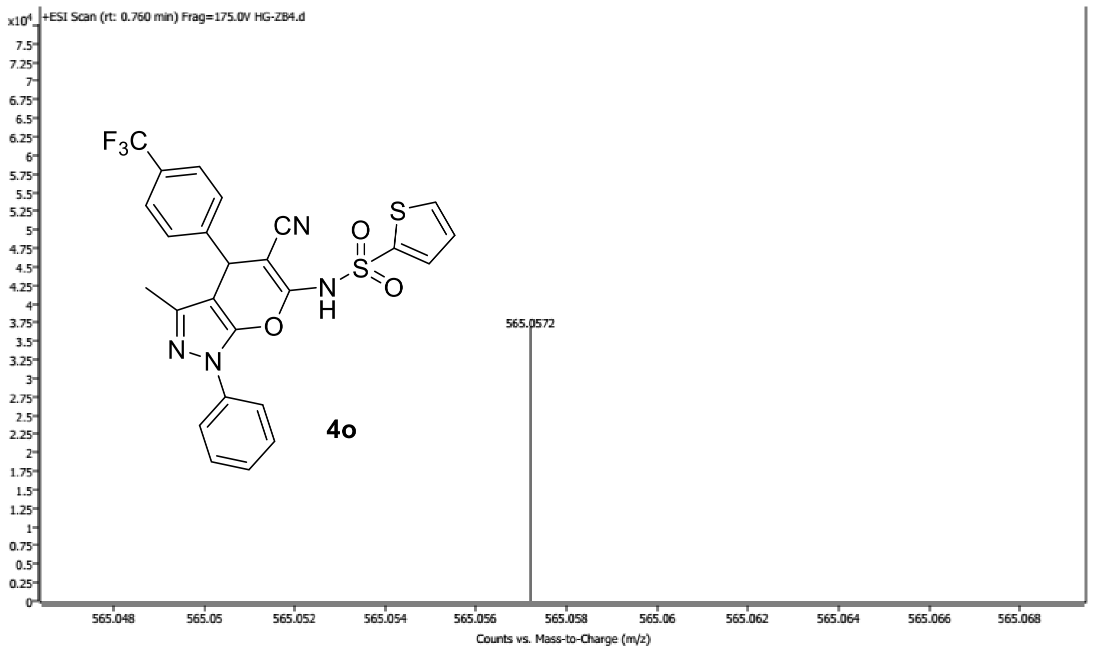
x10^4 +ESI Scan (rt: 0.760 min) Frag=175.0V HG-ZB4.d
565.0572
4o
Counts vs. Mass-to-Charge (m/z)

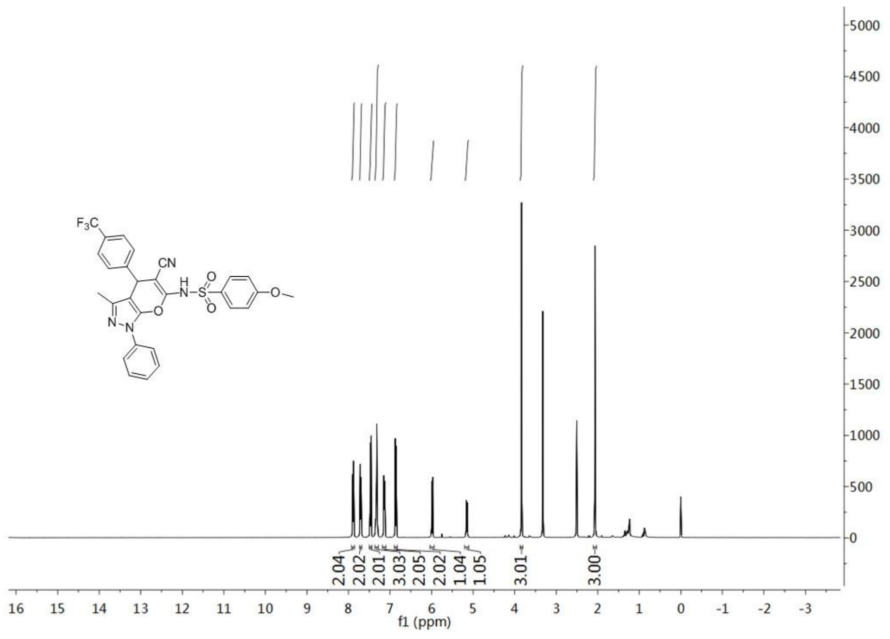
2.04
2.02
2.01
3.03
2.05
2.02
1.04
1.05
3.01
3.00
f1 (ppm)

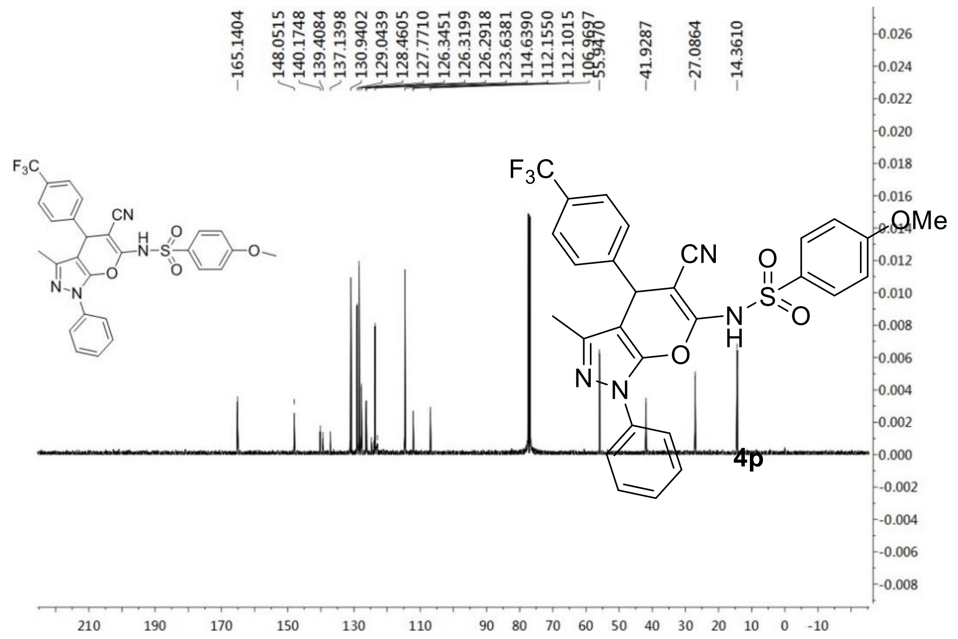
165.1404
148.0515
140.1748
139.4084
137.1398
130.9402
129.0439
128.4605
127.7710
126.3451
126.3199
126.2918
123.6381
114.6390
112.1550
112.1015
106.9697
55.9470
41.9287
27.0864
14.3610
4p
OMe

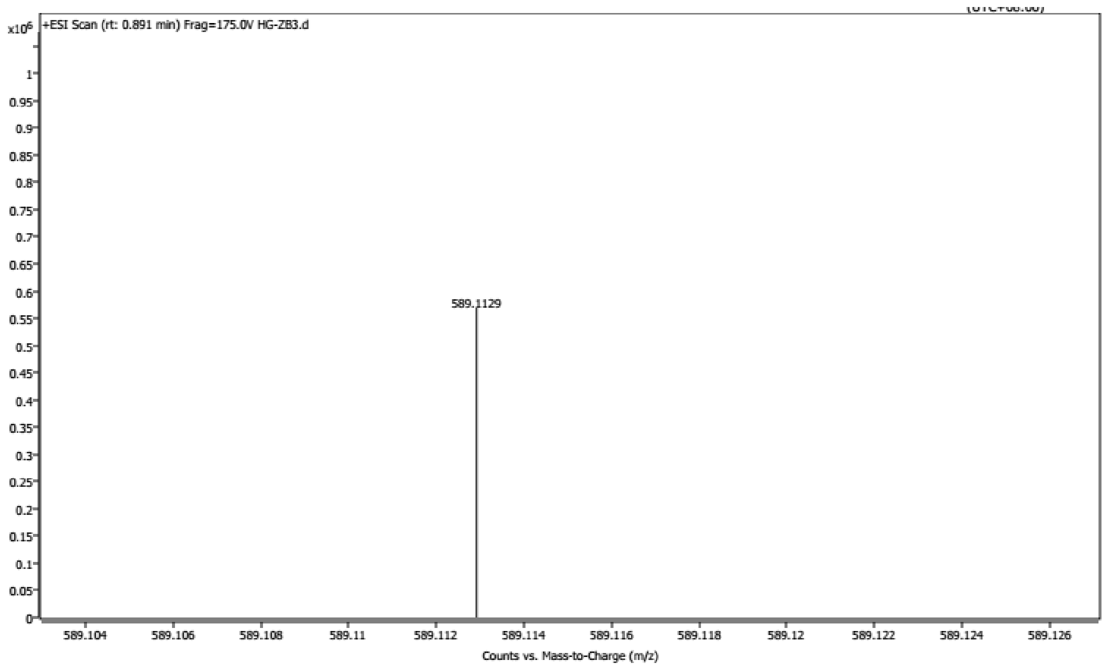
+ESI Scan (rt: 0.891 min) Frag=175.0V HG-ZB3.d
589.1129
Counts vs. Mass-to-Charge (m/z)

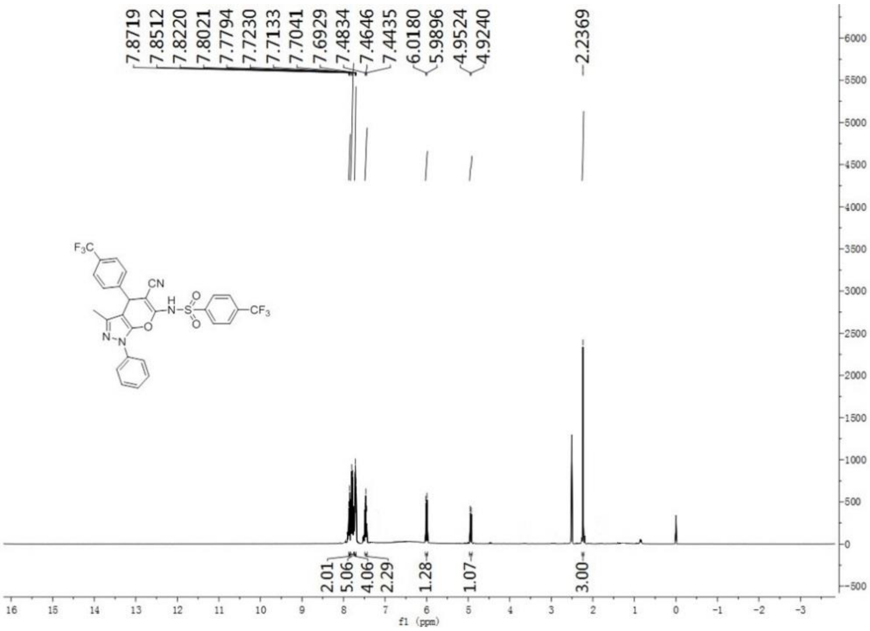
7.8719
7.8512
7.8220
7.8021
7.7794
7.7230
7.7133
7.7041
7.6929
7.4834
7.4646
7.4435
6.0180
5.9896
4.9524
4.9240
2.2369
2.01
5.06
4.06
2.29
1.28
1.07
3.00
f1 (ppm)
F3C
CN
H
N
S
O
CF3
N
N
O

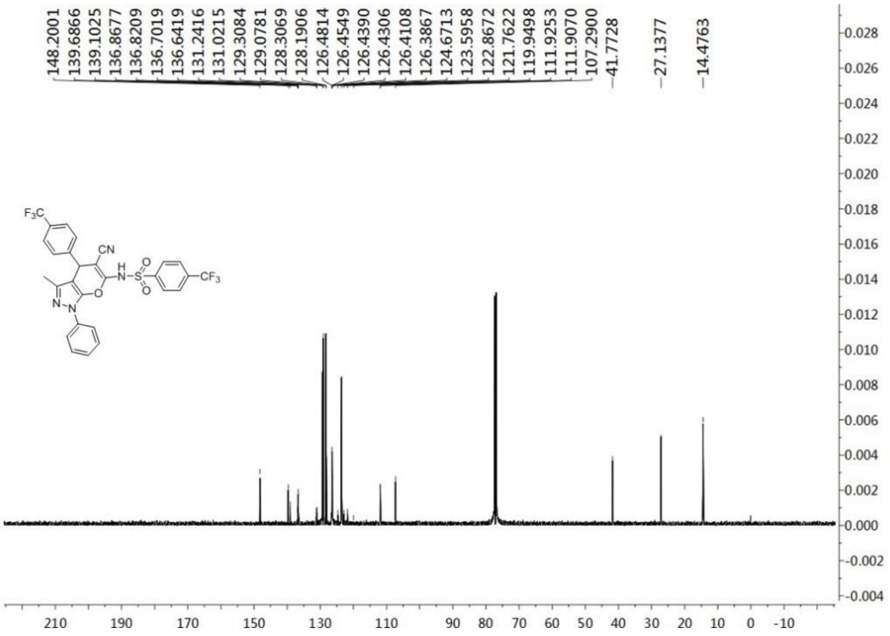
148.2001
139.6866
139.1025
136.8677
136.8209
136.7019
136.6419
131.2416
131.0215
129.3084
129.0781
128.3069
128.1906
126.4814
126.4549
126.4390
126.4306
126.4108
126.3867
124.6713
123.5958
122.8672
121.7622
119.9498
111.9253
111.9070
107.2900
41.7728
27.1377
14.4763
F3C
CN
H
N
S
O
CF3
N
N
O

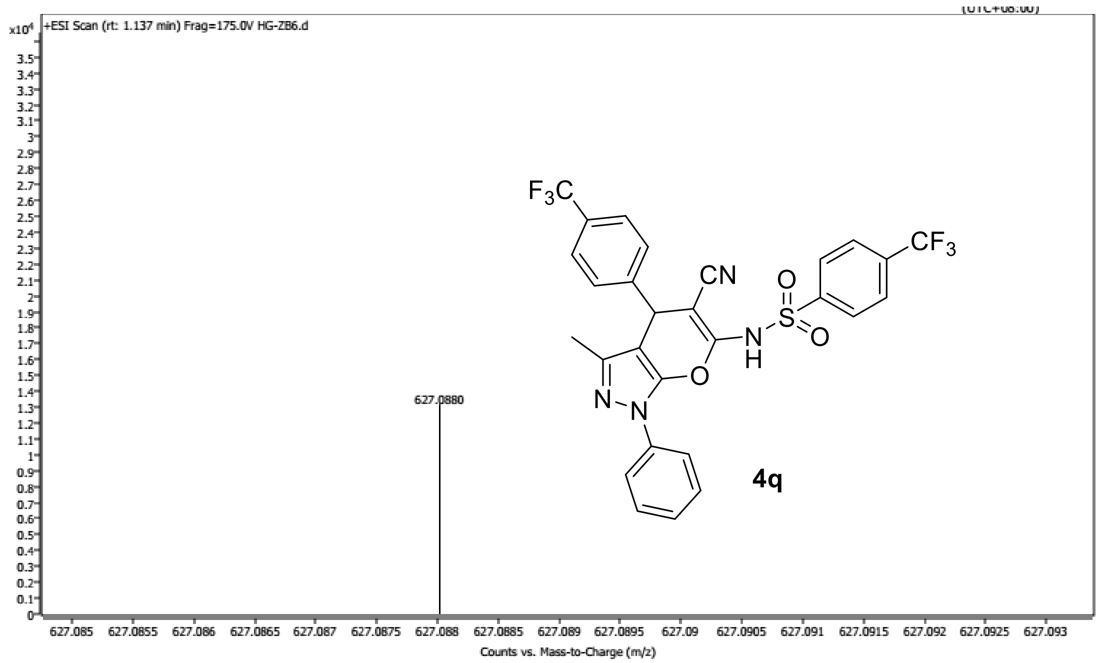
+ESI Scan (rt: 1.137 min) Frag=175.0V HG-ZB6.d
x10^4
627.0880
Counts vs. Mass-to-Charge (m/z)
4q

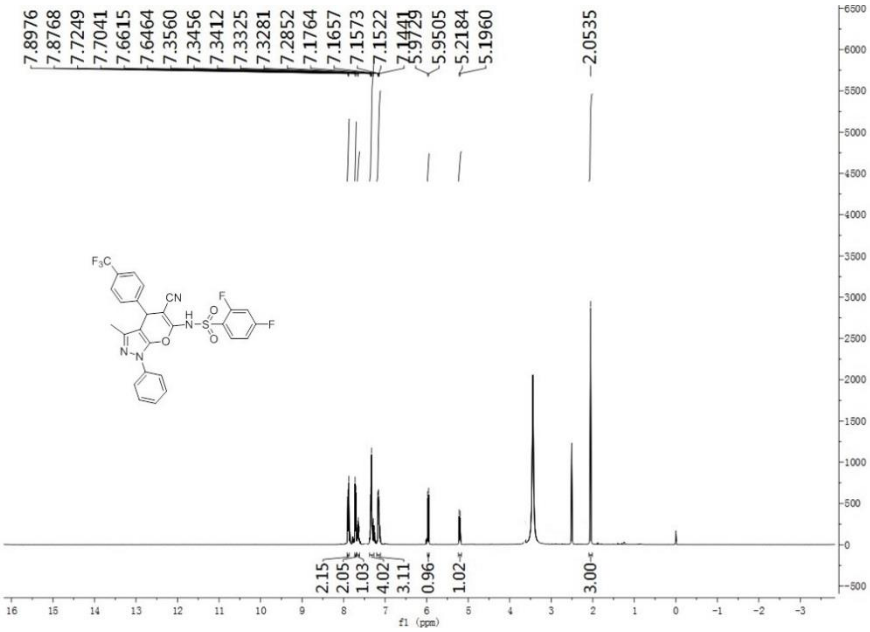
7.8976
7.8768
7.7249
7.7041
7.6615
7.6464
7.3560
7.3456
7.3412
7.3325
7.3281
7.2852
7.1764
7.1657
7.1573
7.1522
7.1441
5.9729
5.9505
5.2184
5.1960
2.0535
2.15
2.05
1.03
4.02
3.11
0.96
1.02
3.00
f1 (ppm)

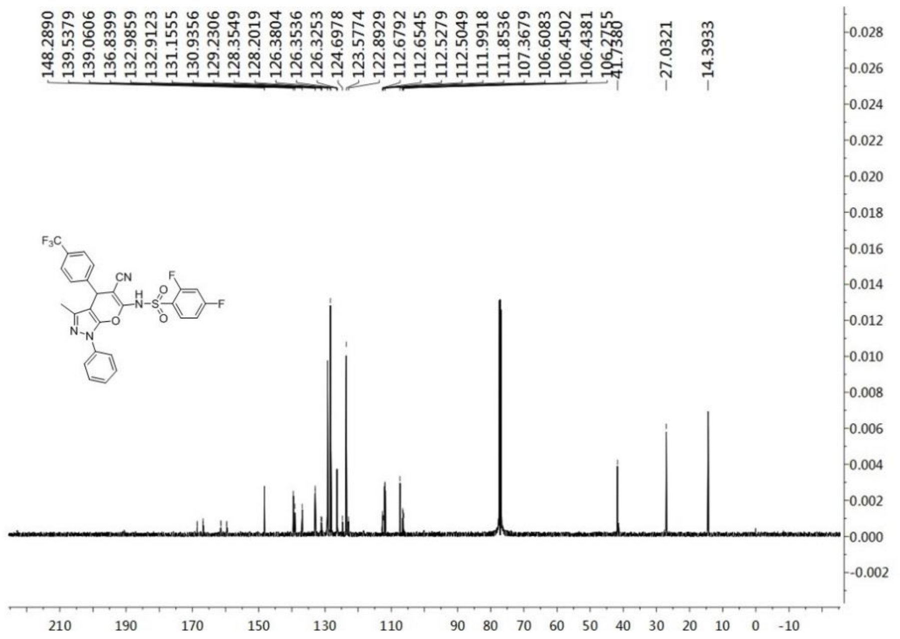

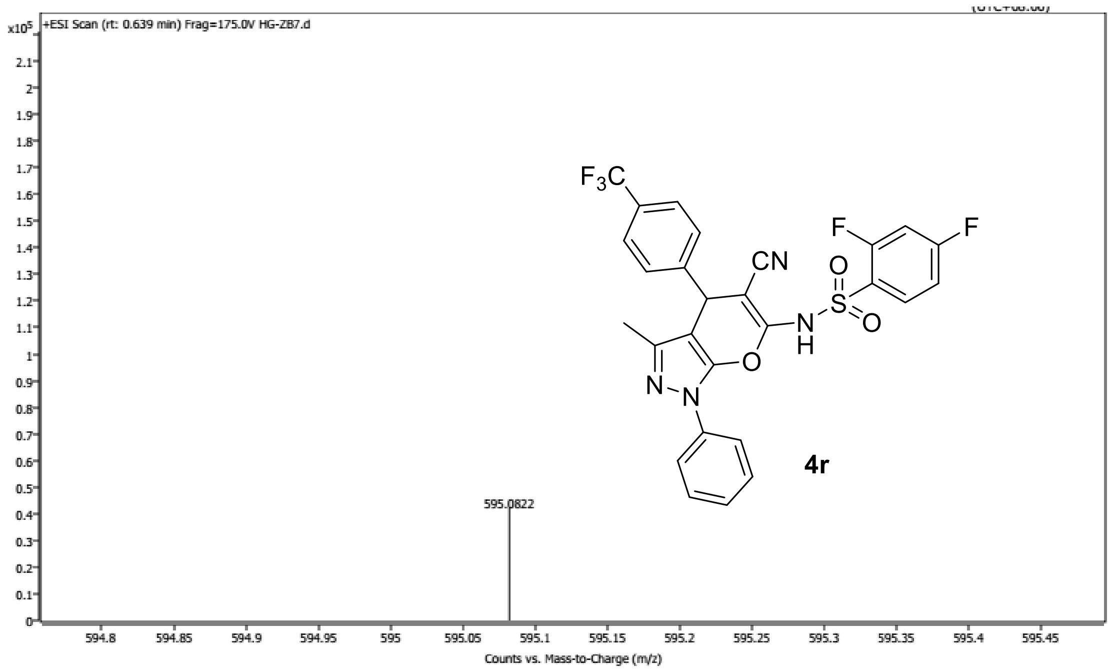
+ESI Scan (rt: 0.639 min) Frag=175.0V HG-ZB7.d
595.0822
Counts vs. Mass-to-Charge (m/z)
4r

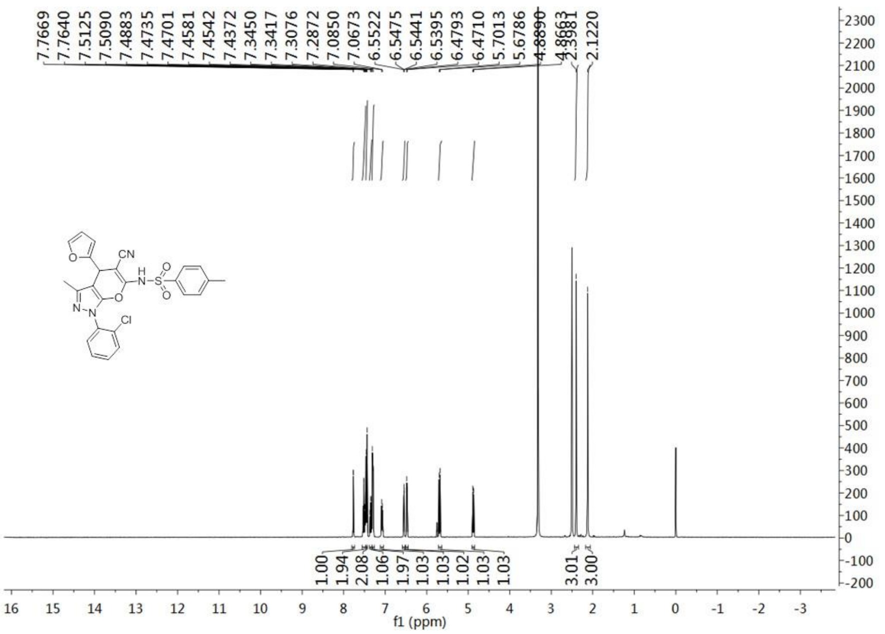

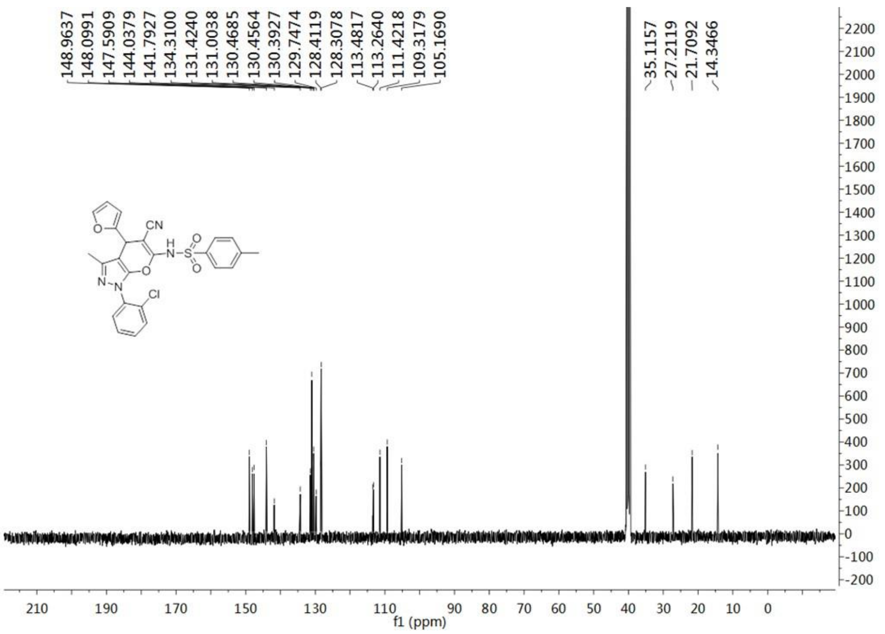

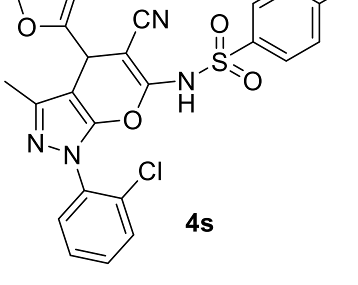
4s

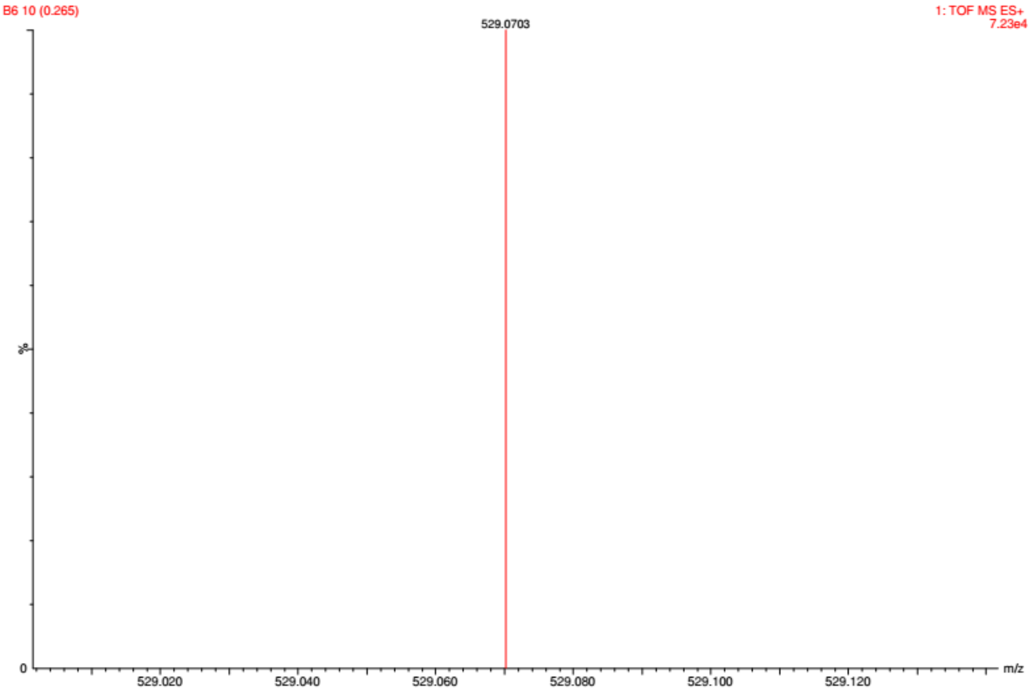
B6 10 (0.265)
529.0703
1: TOF MS ES+
7.23e4
%
m/z
529.020
529.040
529.060
529.080
529.100
529.120

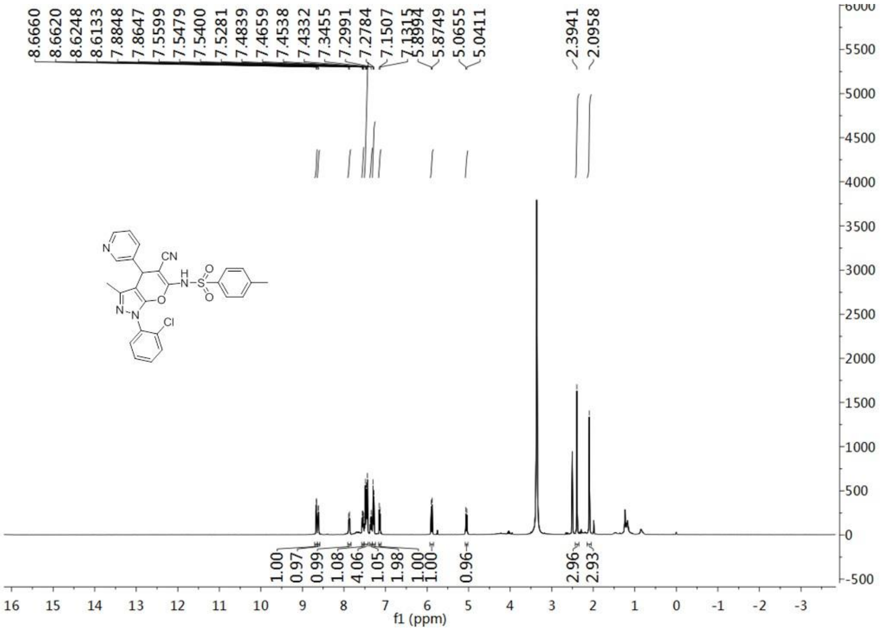
f1 (ppm)

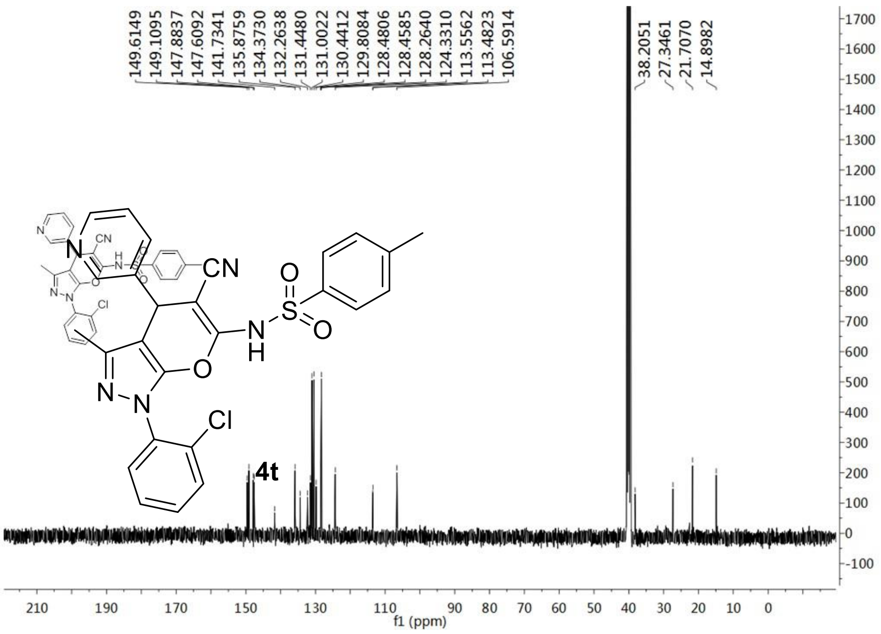
149.6149
149.1095
147.8837
147.6092
141.7341
135.8759
134.3730
132.2638
131.4480
131.0022
130.4412
129.8084
128.4806
128.4585
128.2640
124.3310
113.5562
113.4823
106.5914
38.2051
27.3461
21.7070
14.8982
4t
f1 (ppm)

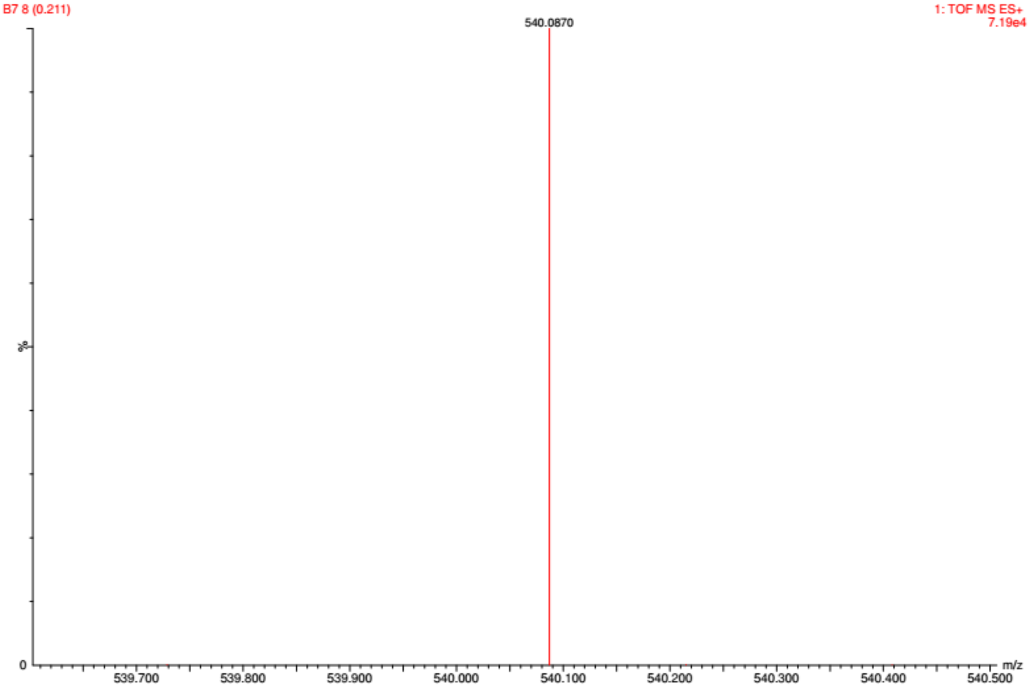
B7 8 (0.211)
540.0870
1: TOF MS ES+
7.19e4
%
m/z

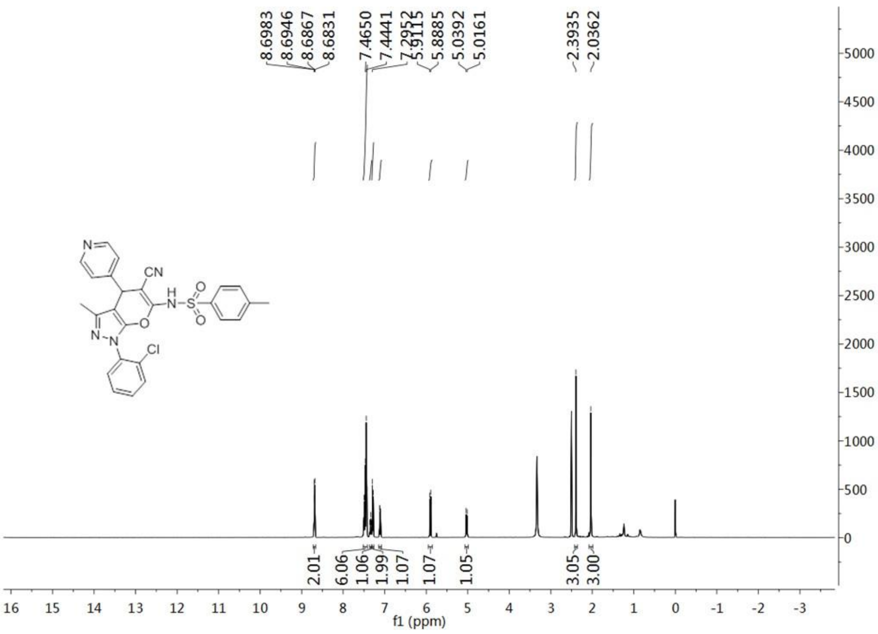

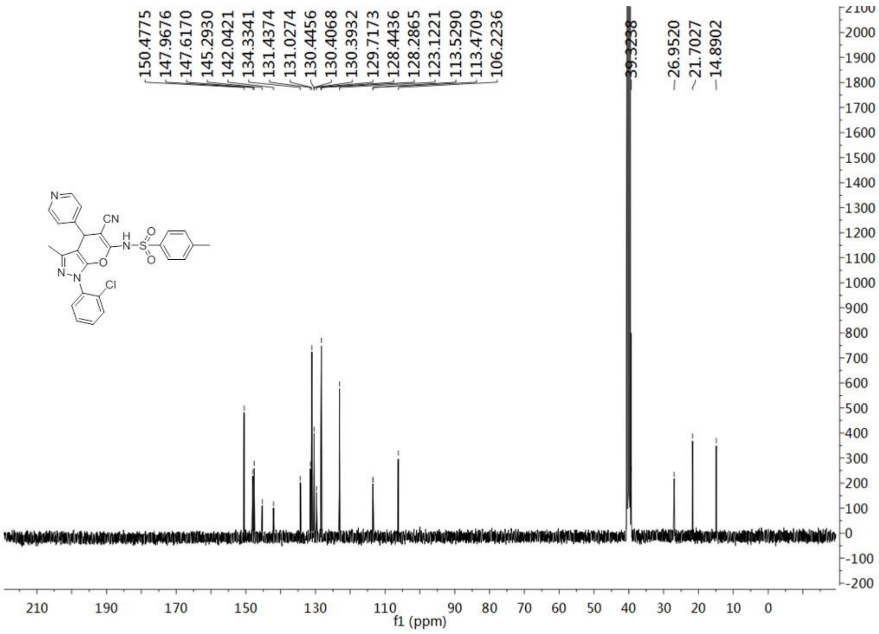

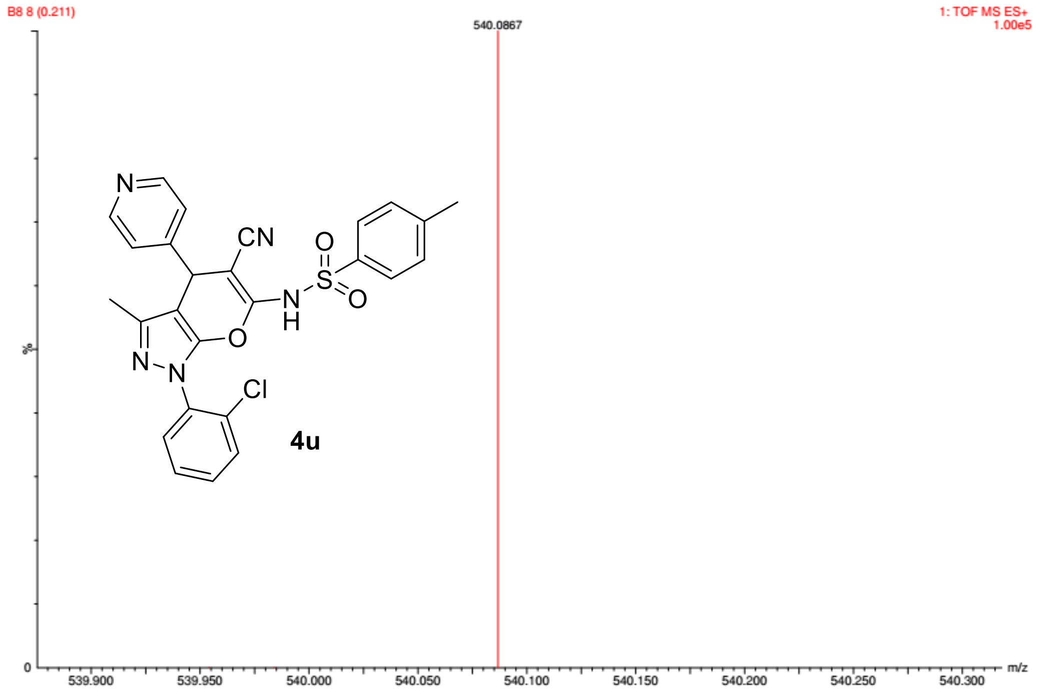
B8 8 (0.211)
540.0867
1: TOF MS ES+
1.00e5
%
0
539.900
539.950
540.000
540.050
540.100
540.150
540.200
540.250
540.300
m/z
N
CN
O
S
O
N
H
O
N
N
Cl
4u

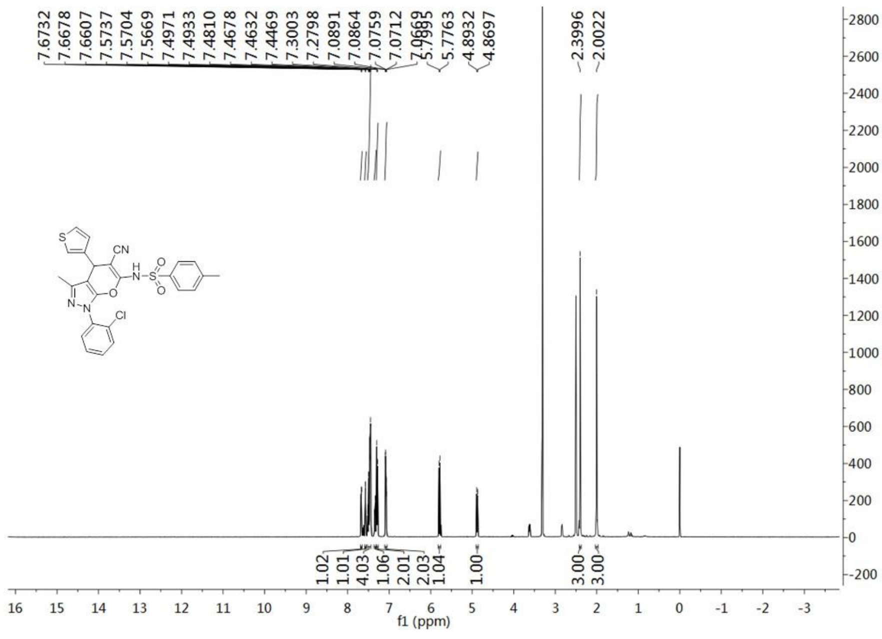

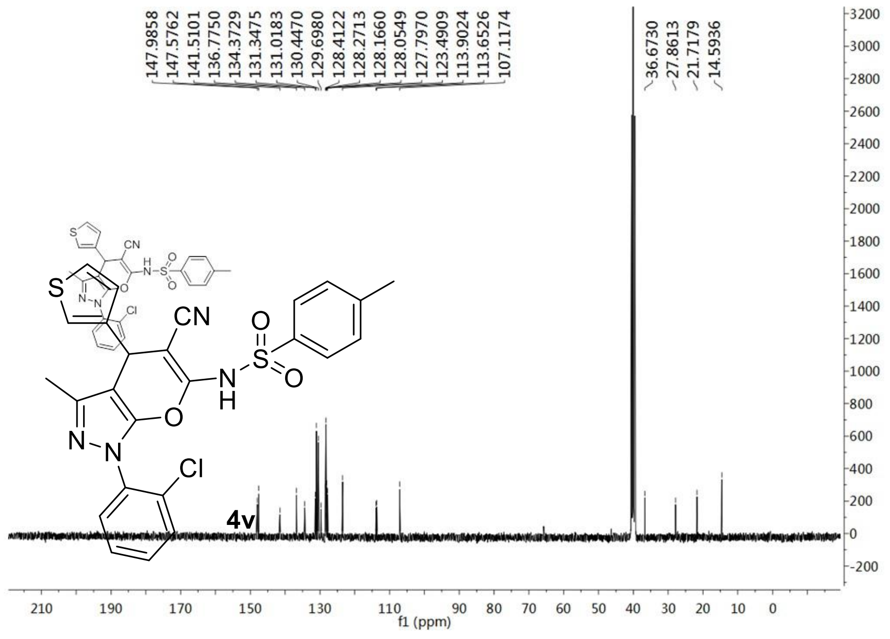
147.9858
147.5762
141.5101
136.7750
134.3729
131.3475
131.0183
130.4470
129.6980
128.4122
128.2713
128.1660
128.0549
127.7970
123.4909
113.9024
113.6526
107.1174
36.6730
27.8613
21.7179
14.5936
4v
f1 (ppm)

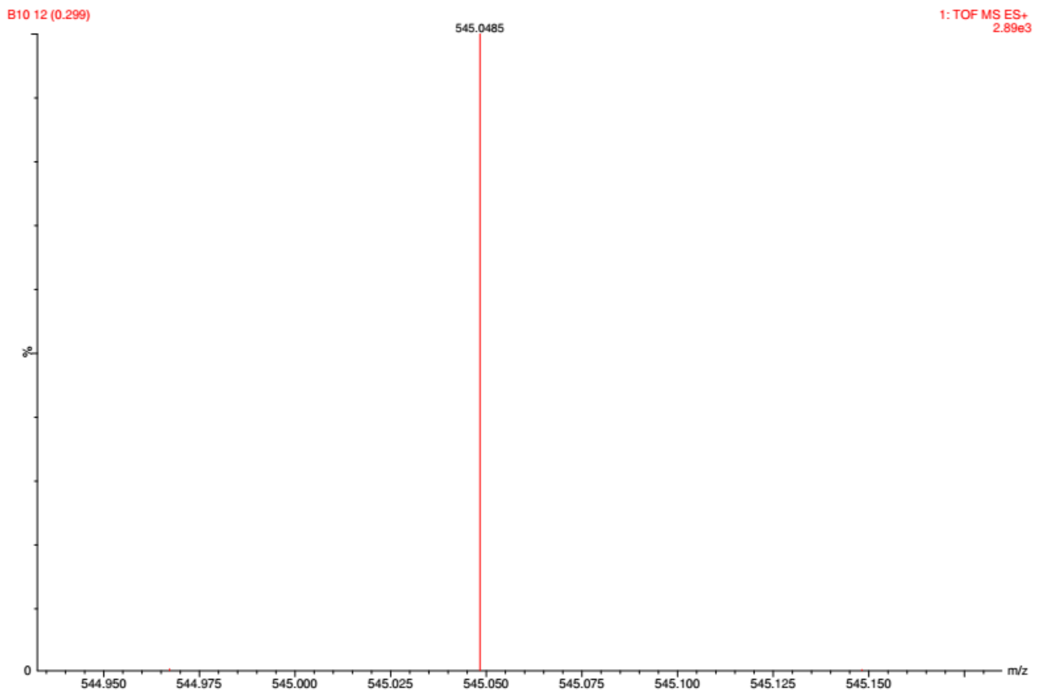
B10 12 (0.299)
545.0485
1: TOF MS ES+
2.89e3
%
m/z

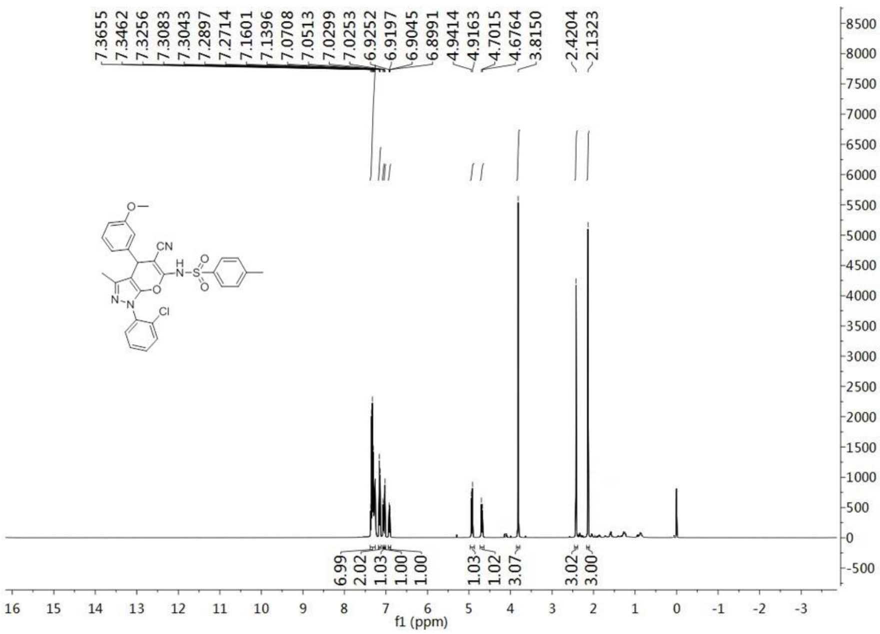

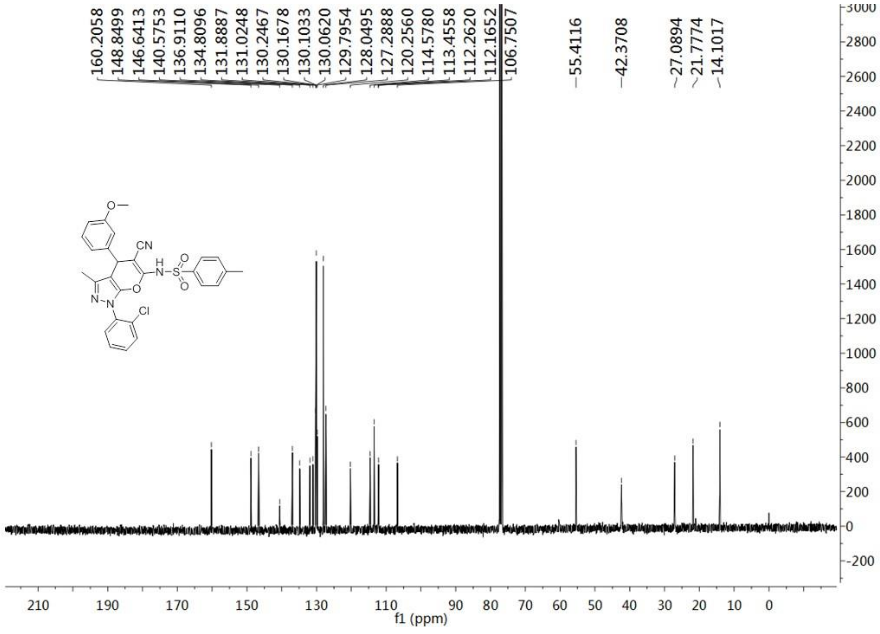

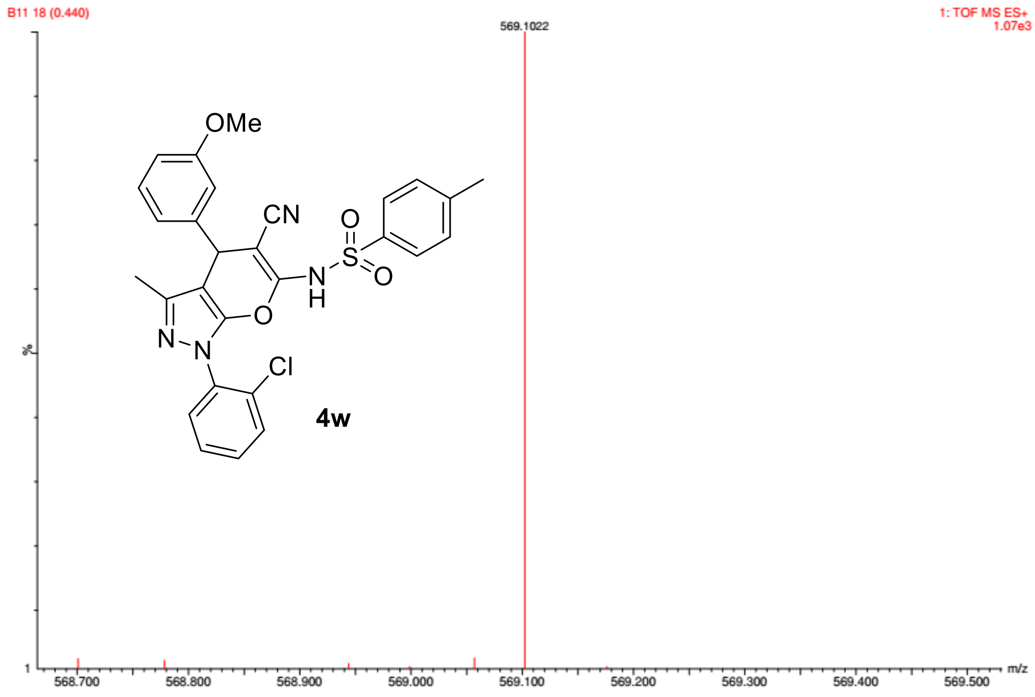
B11 18 (0.440)
1: TOF MS ES+
1.07e3
569.1022
OMe
CN
O
S
N
H
O
N
N
O
Cl
4w
%
m/z

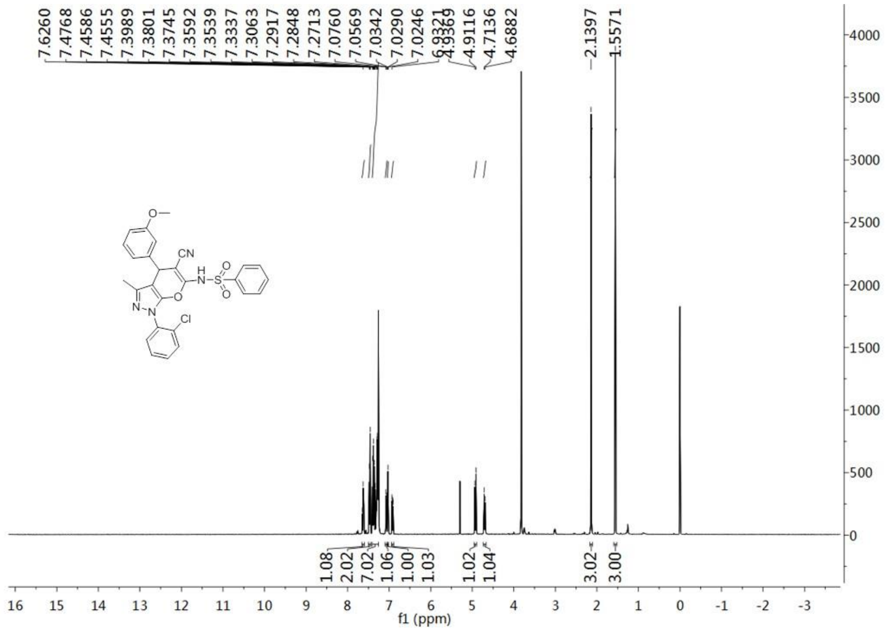
f1 (ppm)

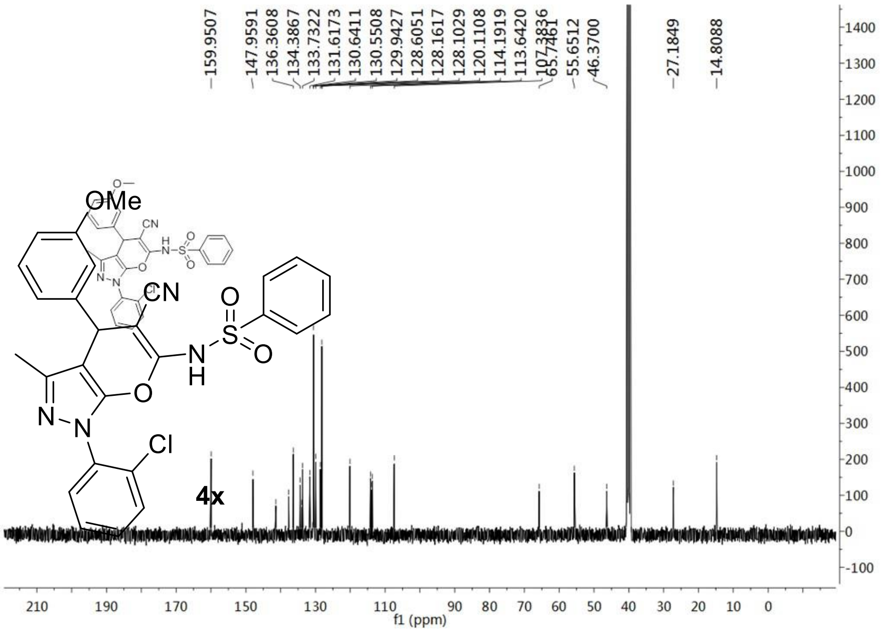
159.9507
147.9591
136.3608
134.3867
133.7322
131.6173
130.6411
130.5508
129.9427
128.6051
128.1617
128.1029
120.1108
114.1919
113.6420
107.3836
65.7461
55.6512
46.3700
27.1849
14.8088
OMe
CN
O
S
N
H
O
N
N
Cl
4x
f1 (ppm)

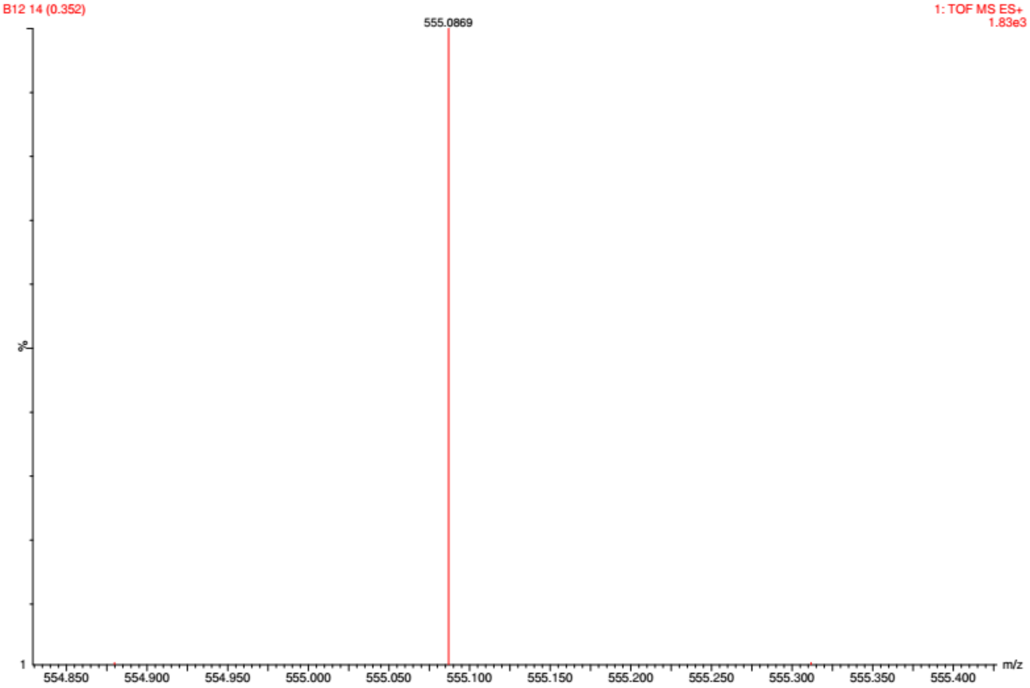
B12 14 (0.352)
1: TOF MS ES+
1.83e3
555.0869
%
m/z

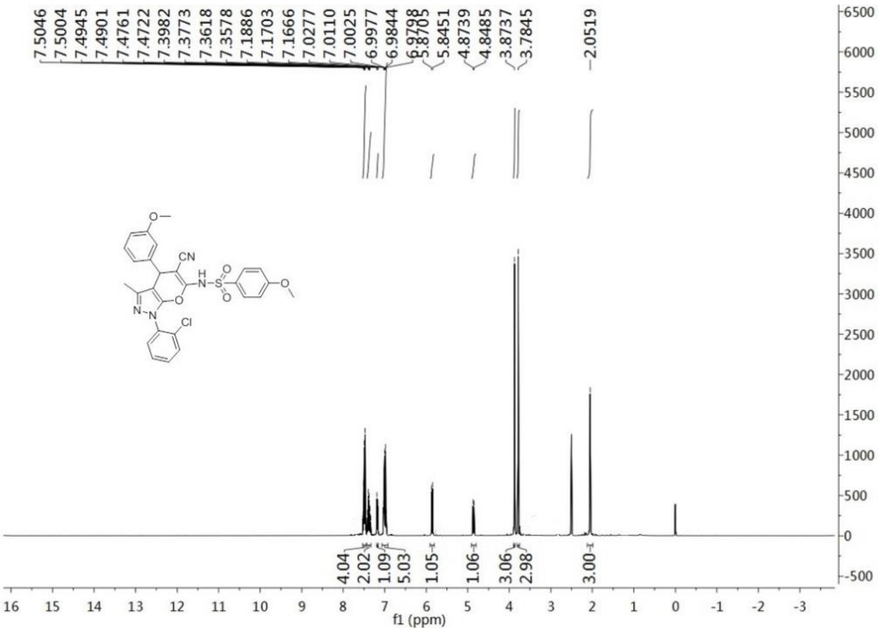

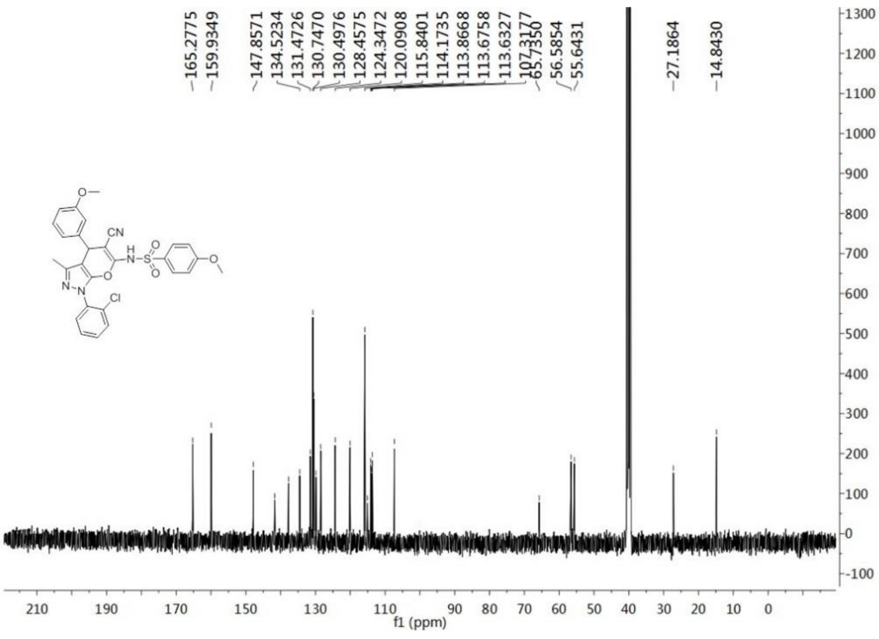

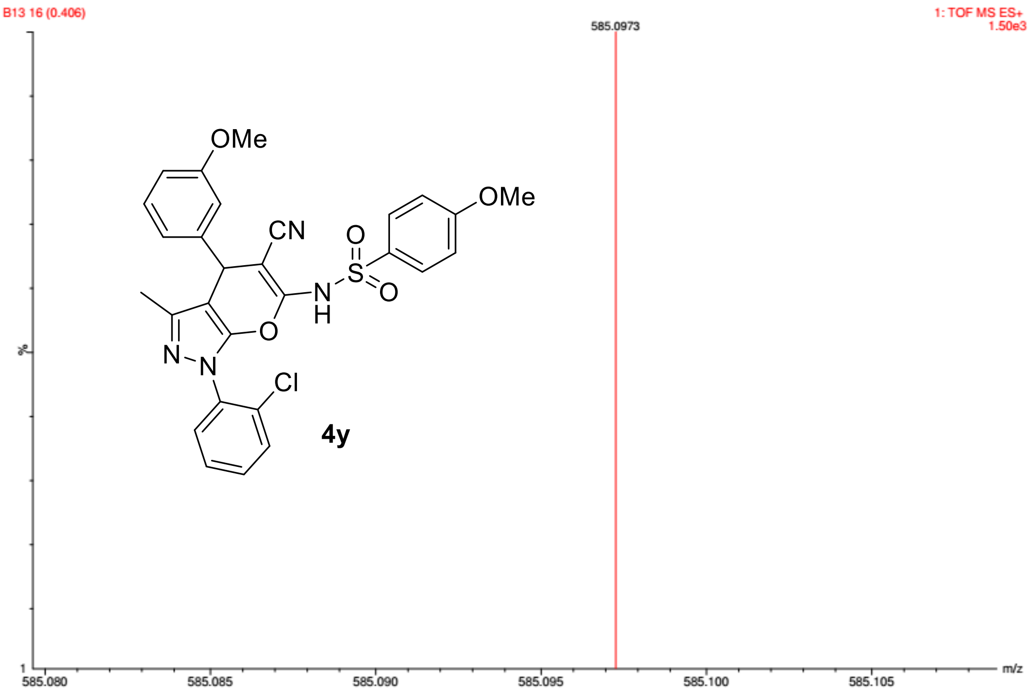
B13 16 (0.406)
1: TOF MS ES+
1.50e3
585.0973
%
m/z
585.080
585.085
585.090
585.095
585.100
585.105
OMe
OMe
CN
N
H
O
S
O
N
N
O
Cl
4y

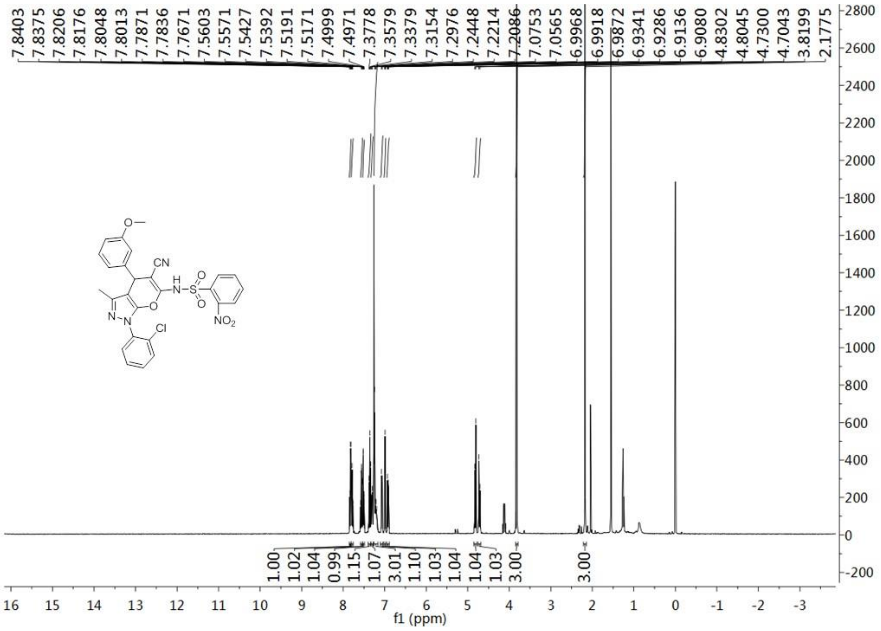
7.8403
7.8375
7.8206
7.8176
7.8048
7.8013
7.7871
7.7836
7.7671
7.5603
7.5571
7.5427
7.5392
7.5191
7.5171
7.4999
7.4971
7.3778
7.3579
7.3379
7.3154
7.2976
7.2448
7.2214
7.2086
7.0753
7.0565
6.9968
6.9918
6.9872
6.9341
6.9286
6.9136
6.9080
4.8302
4.8045
4.7300
4.7043
3.8199
2.1775
1.00
1.02
1.04
0.99
1.15
1.07
3.01
1.10
1.03
1.04
1.04
1.03
3.00
3.00
f1 (ppm)
CN
Cl
NO2

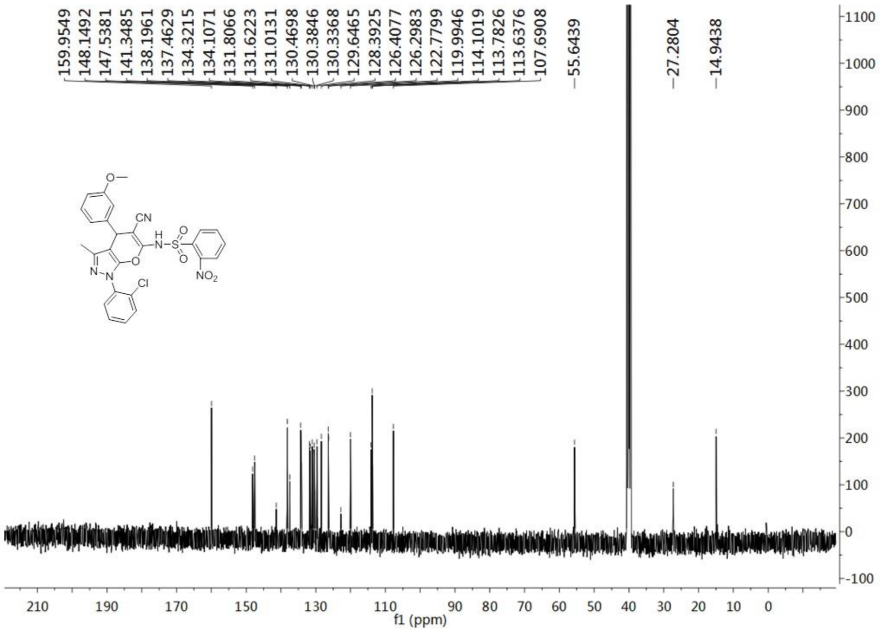
159.9549
148.1492
147.5381
141.3485
138.1961
137.4629
134.3215
134.1071
131.8066
131.6223
131.0131
130.4698
130.3846
130.3368
129.6465
128.3925
126.4077
126.2983
122.7799
119.9946
114.1019
113.7826
113.6376
107.6908
55.6439
27.2804
14.9438
CN
NO2
Cl
f1 (ppm)

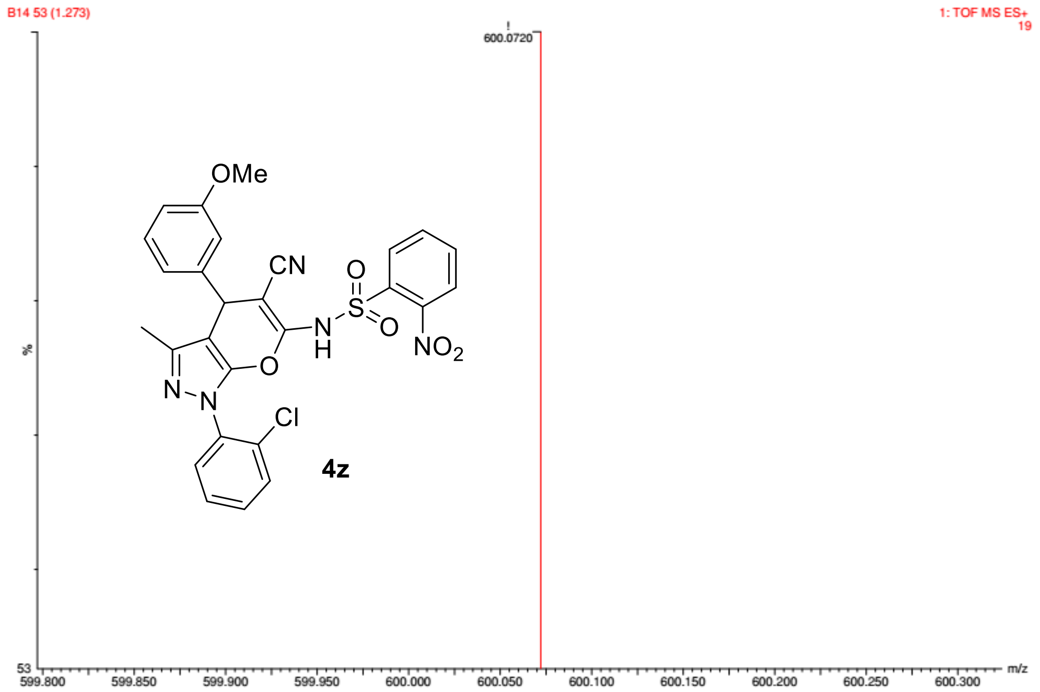
B14 53 (1.273)
1: TOF MS ES+
19
600.0720
%
53
OMe
CN
O
S
O
N
H
NO2
N
N
O
Cl
4z
599.800
599.850
599.900
599.950
600.000
600.050
600.100
600.150
600.200
600.250
600.300
m/z

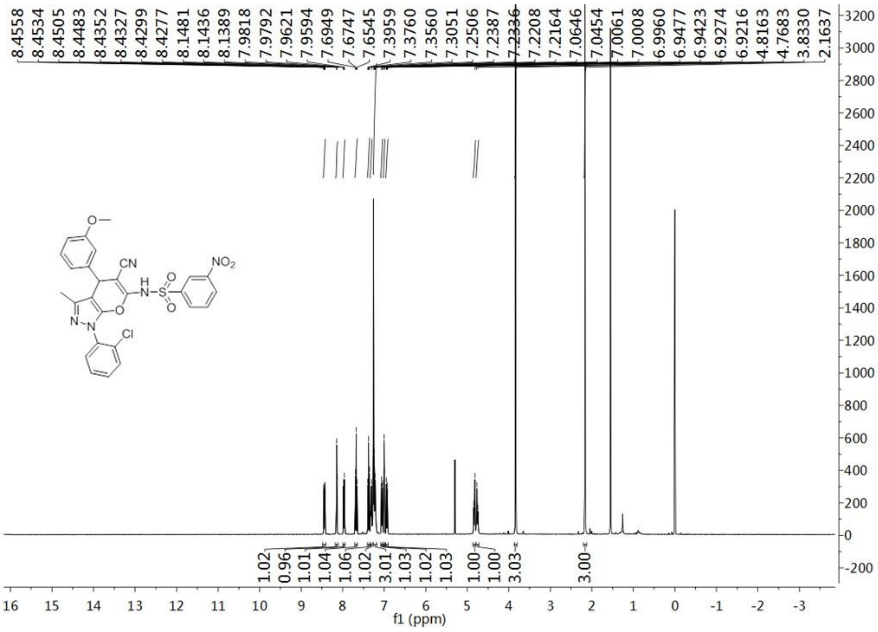
8.4558
8.4534
8.4505
8.4483
8.4352
8.4327
8.4299
8.4277
8.1481
8.1436
8.1389
7.9818
7.9792
7.9621
7.9594
7.6949
7.6747
7.6545
7.3959
7.3760
7.3560
7.3051
7.2506
7.2387
7.2336
7.2208
7.2164
7.0646
7.0454
7.0061
7.0008
6.9960
6.9477
6.9423
6.9274
6.9216
4.8163
4.7683
3.8330
2.1637
CN
NO2
H
N
S
O
Cl
1.02
0.96
1.01
1.04
1.06
1.02
3.01
1.03
1.02
1.03
1.00
1.00
3.03
3.00
f1 (ppm)

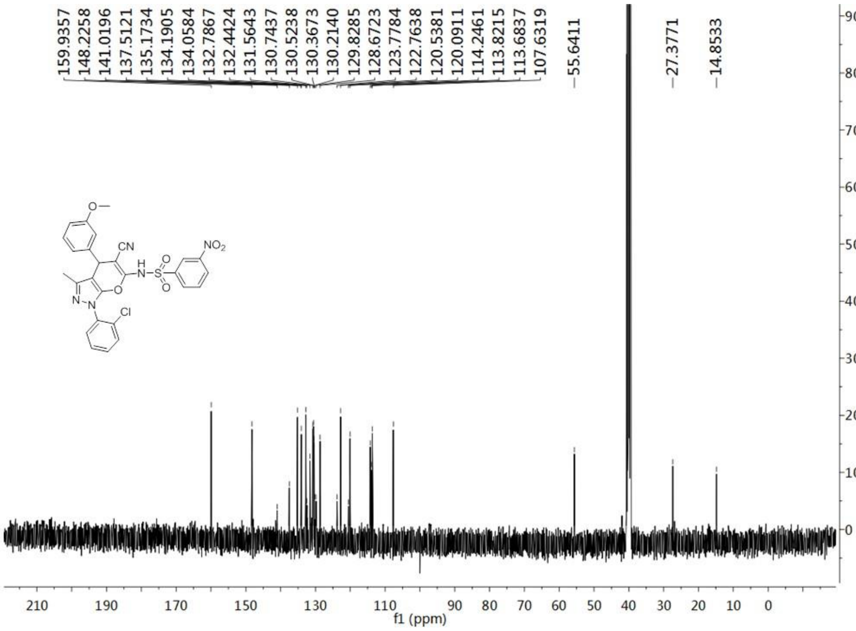
159.9357
148.2258
141.0196
137.5121
135.1734
134.1905
134.0584
132.7867
132.4424
131.5643
130.7437
130.5238
130.3673
130.2140
129.8285
128.6723
123.7784
122.7638
120.5381
120.0911
114.2461
113.8215
113.6837
107.6319
55.6411
27.3771
14.8533
CN
NO2
H
N
S
O
Cl
f1 (ppm)

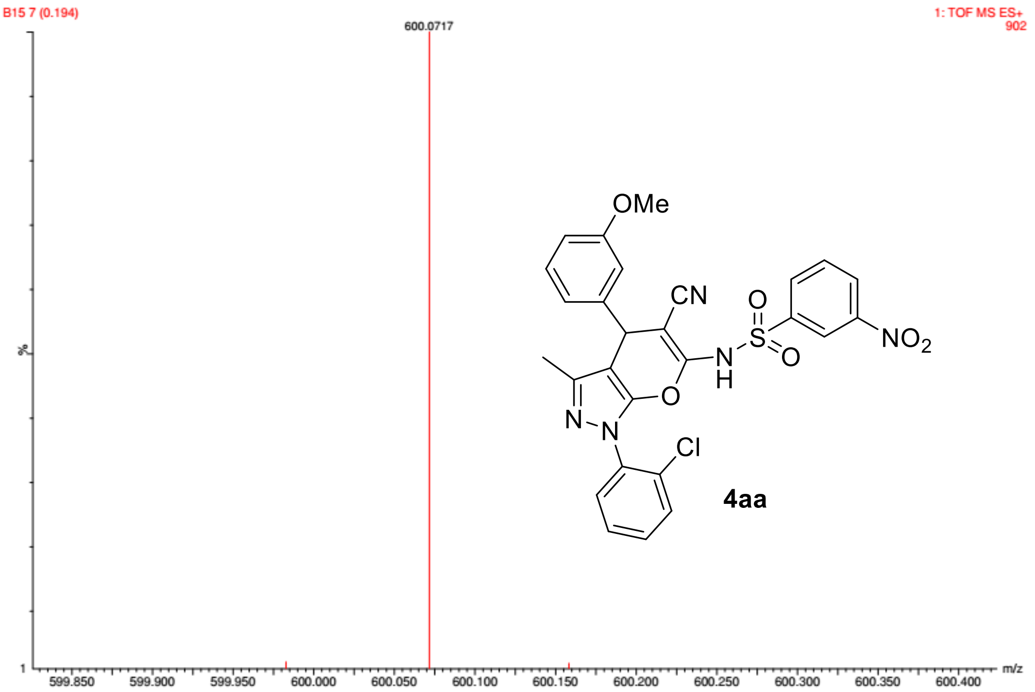
B15 7 (0.194)
1: TOF MS ES+
902
600.0717
%
m/z
OMe
CN
NO2
Cl
4aa

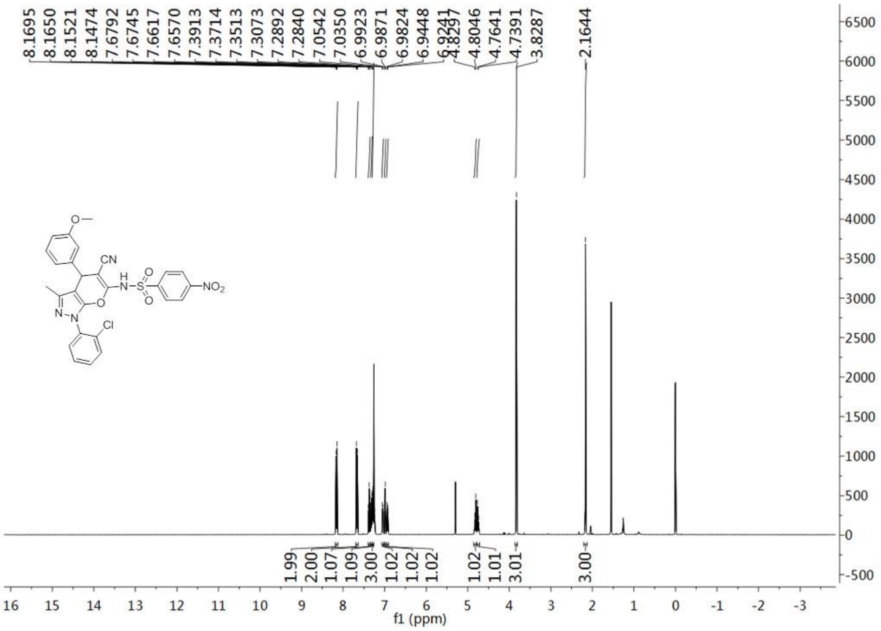
f1 (ppm)

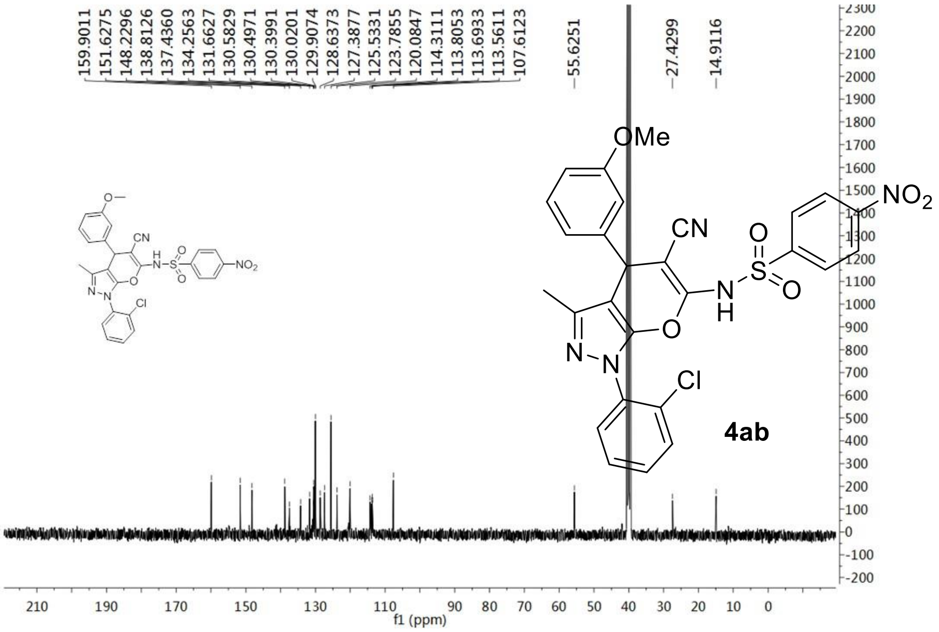
159.9011
151.6275
148.2296
138.8126
137.4360
134.2563
131.6627
130.5829
130.4971
130.3991
130.0201
129.9074
128.6373
127.3877
125.5331
123.7855
120.0847
114.3111
113.8053
113.6933
113.5611
107.6123
55.6251
27.4299
14.9116
OMe
CN
NO2
N
H
S
O
Cl
4ab
f1 (ppm)

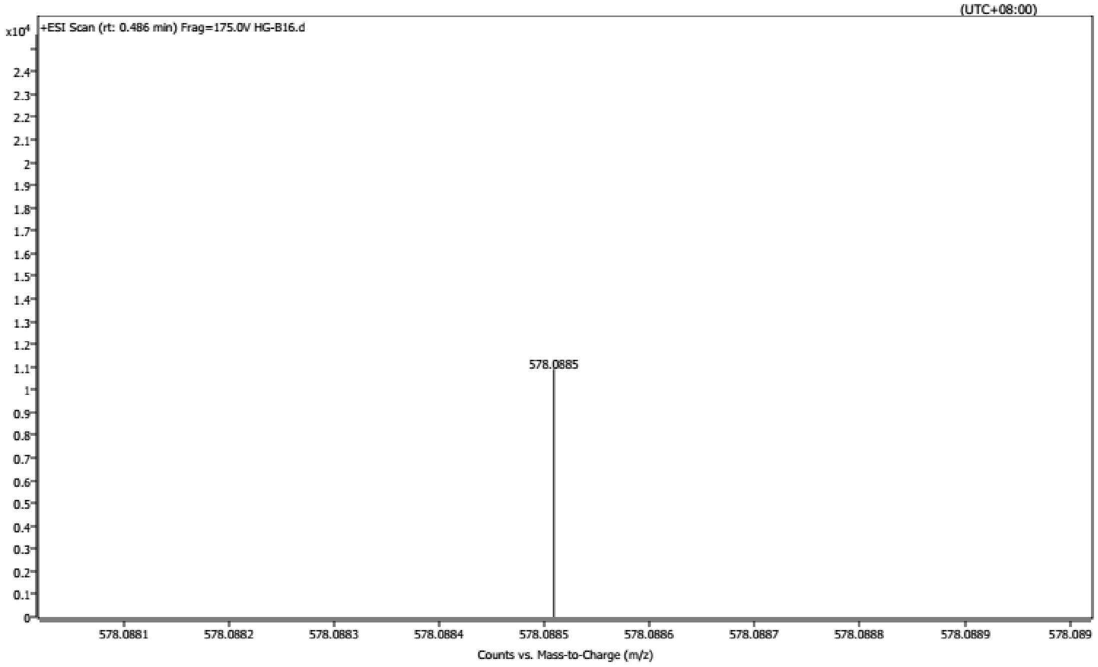
+ESI Scan (rt: 0.486 min) Frag=175.0V HG-B16.d
(UTC+08:00)
x10^4
578.0885
Counts vs. Mass-to-Charge (m/z)

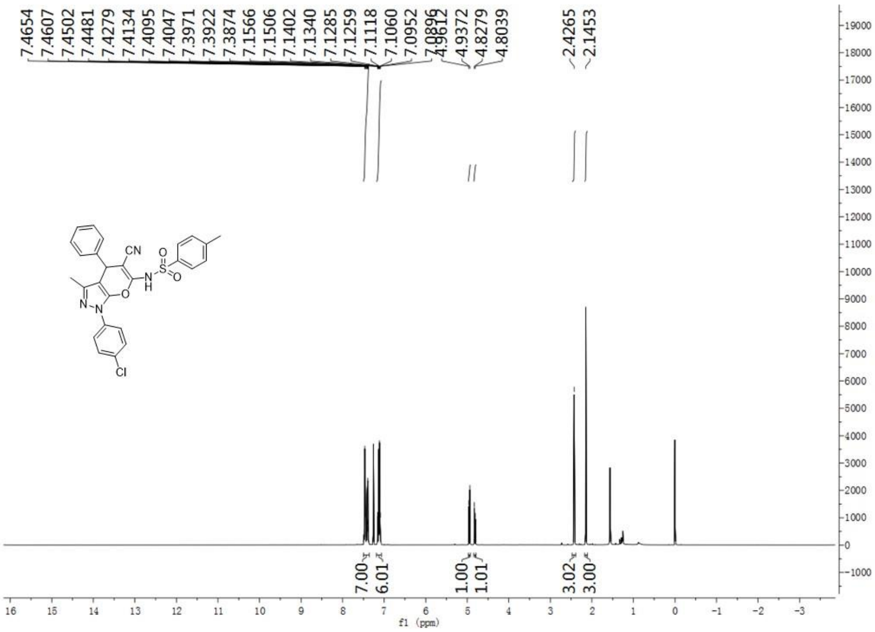
7.4654
7.4607
7.4502
7.4481
7.4279
7.4134
7.4095
7.4047
7.3971
7.3922
7.3874
7.1566
7.1506
7.1402
7.1340
7.1285
7.1259
7.1118
7.1060
7.0952
7.0896
4.9612
4.9372
4.8279
4.8039
2.4265
2.1453
CN
NH
O
S
N
N
Cl
7.00
6.01
1.00
1.01
3.02
3.00
f1 (ppm)

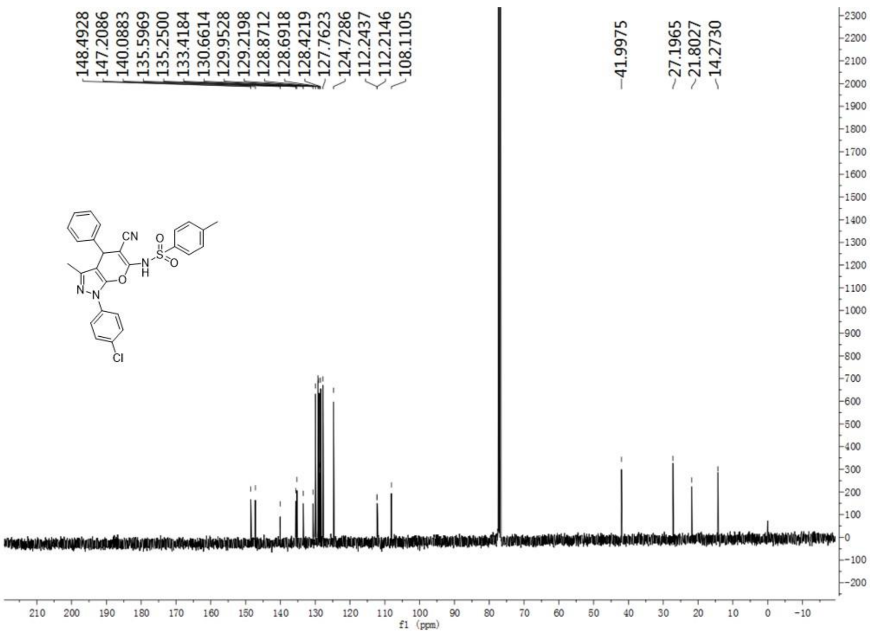
148.4928
147.2086
140.0883
135.5969
135.2500
133.4184
130.6614
129.9528
129.2198
128.8712
128.6918
128.4219
127.7623
124.7286
112.2437
112.2146
108.1105
41.9975
27.1965
21.8027
14.2730
CN
NH
O
S
N
N
Cl
f1 (ppm)

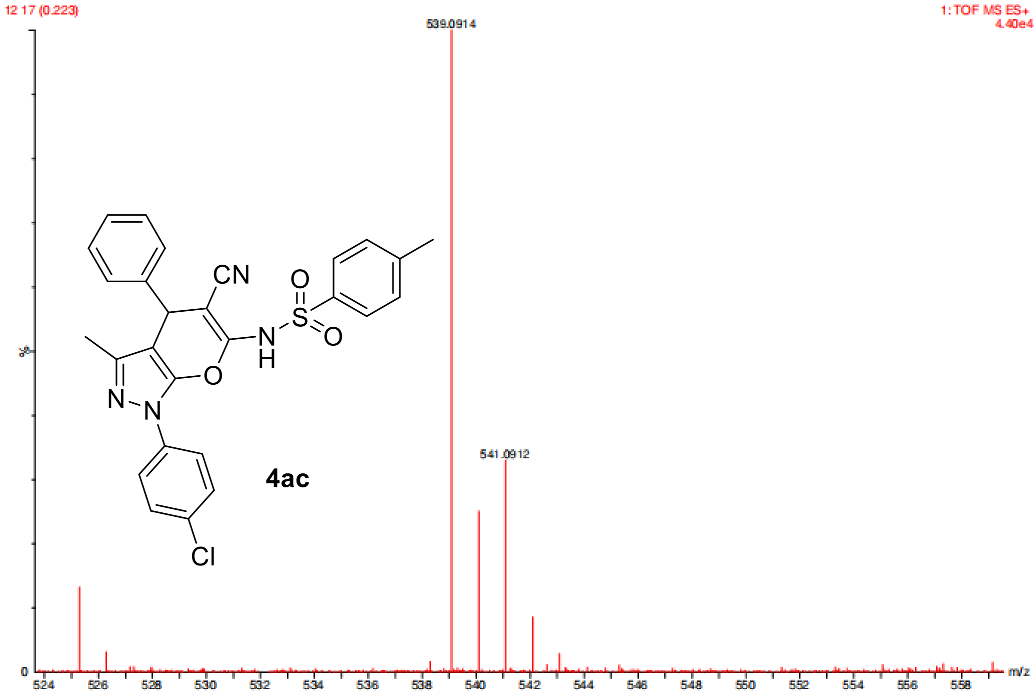
12 17 (0.223)
1: TOF MS ES+
4.40e4
539.0914
541.0912
4ac
m/z

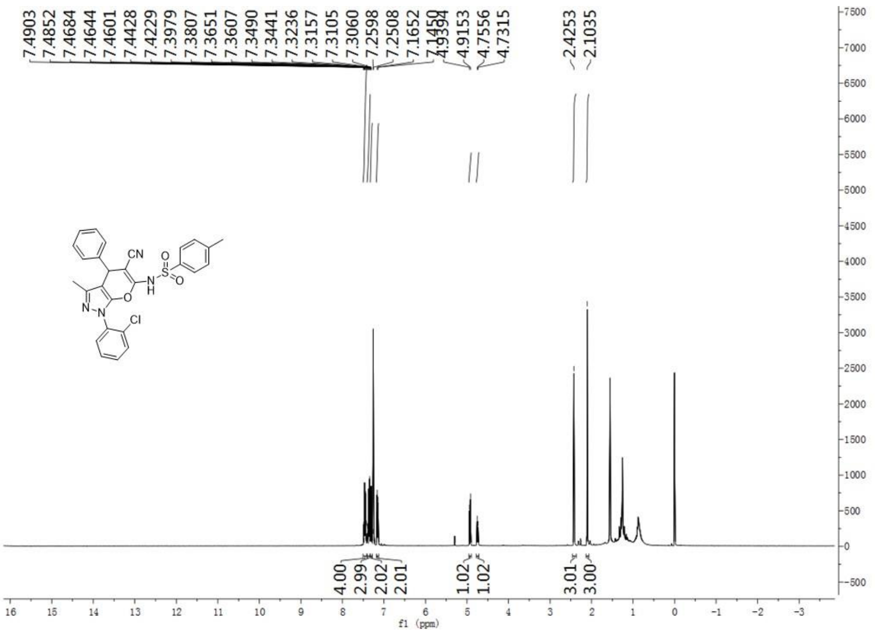
f1 (ppm)

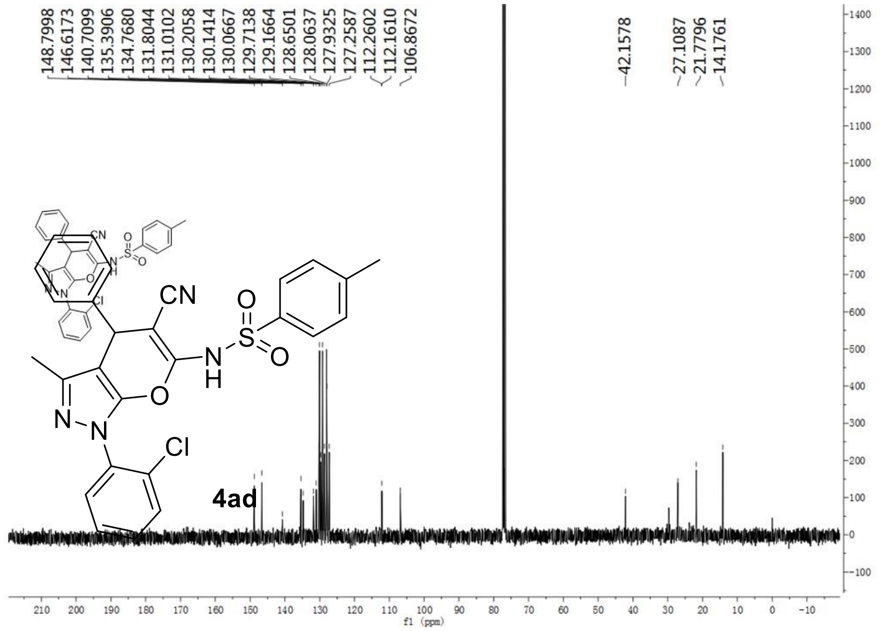
148.7998
146.6173
140.7099
135.3906
134.7680
131.8044
131.0102
130.2058
130.1414
130.0667
129.7138
129.1664
128.6501
128.0637
127.9325
127.2587
112.2602
112.1610
106.8672
42.1578
27.1087
21.7796
14.1761
4ad
f1 (ppm)

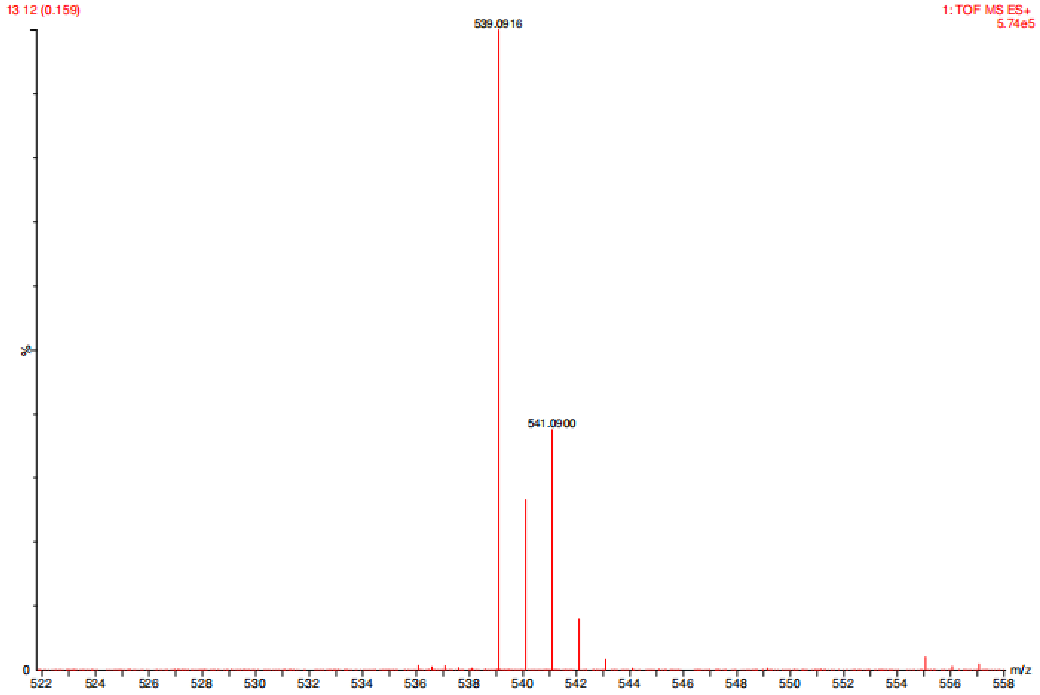
13 12 (0.159)
1: TOF MS ES+
5.74e5
539.0916
541.0900
%
m/z

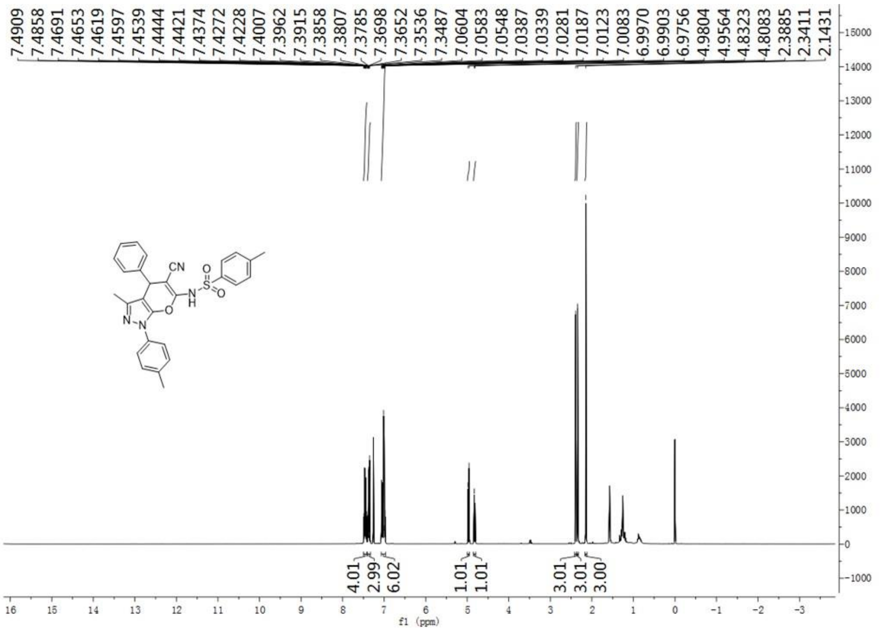

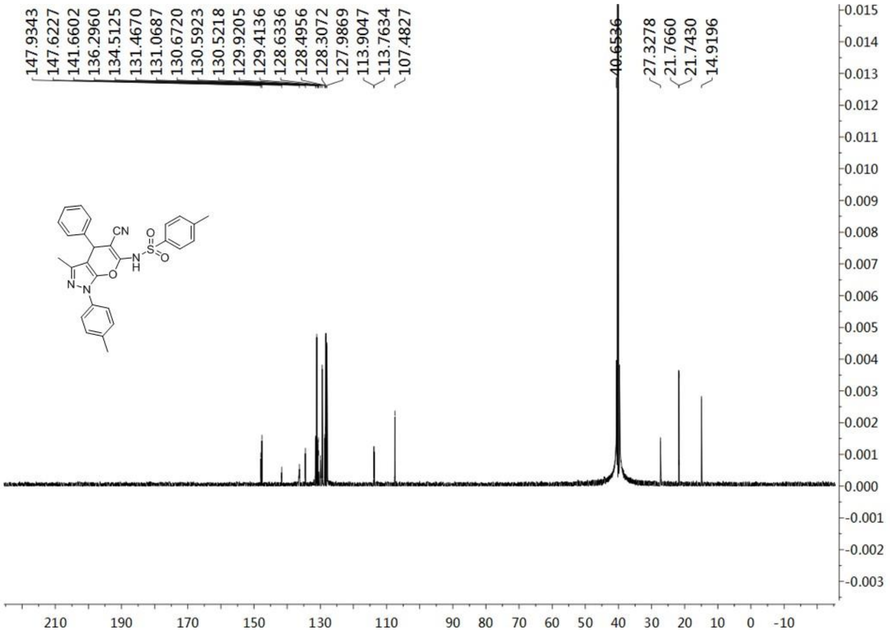

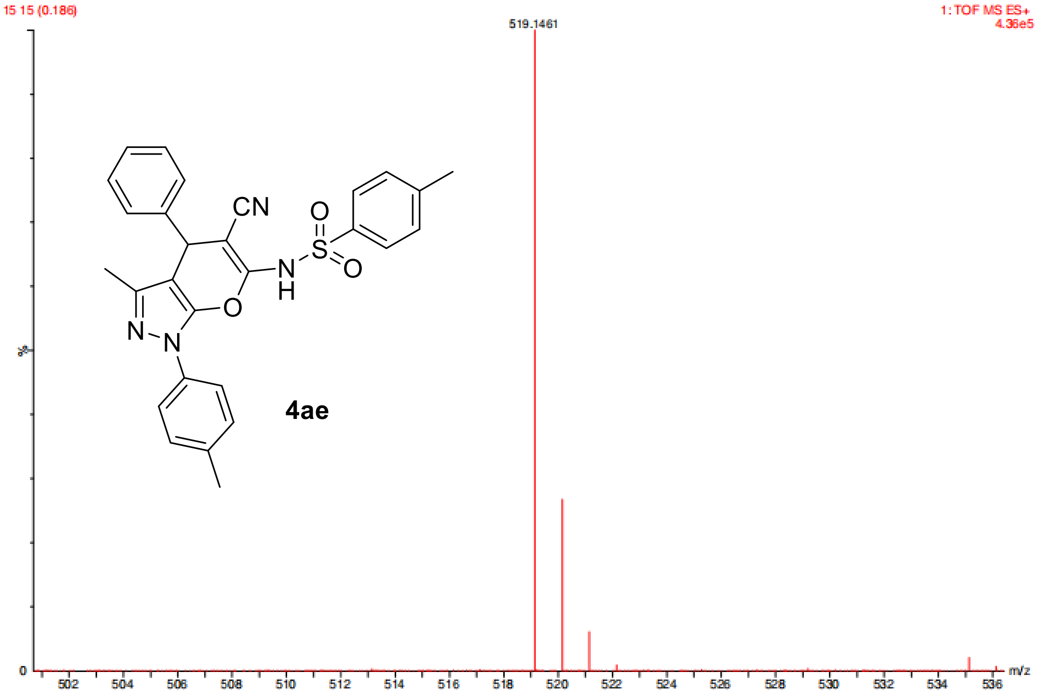
15 15 (0.186)
1: TOF MS ES+
4.36e5
519.1461
%
CN
O
S
O
N
H
O
N
N
4ae
m/z

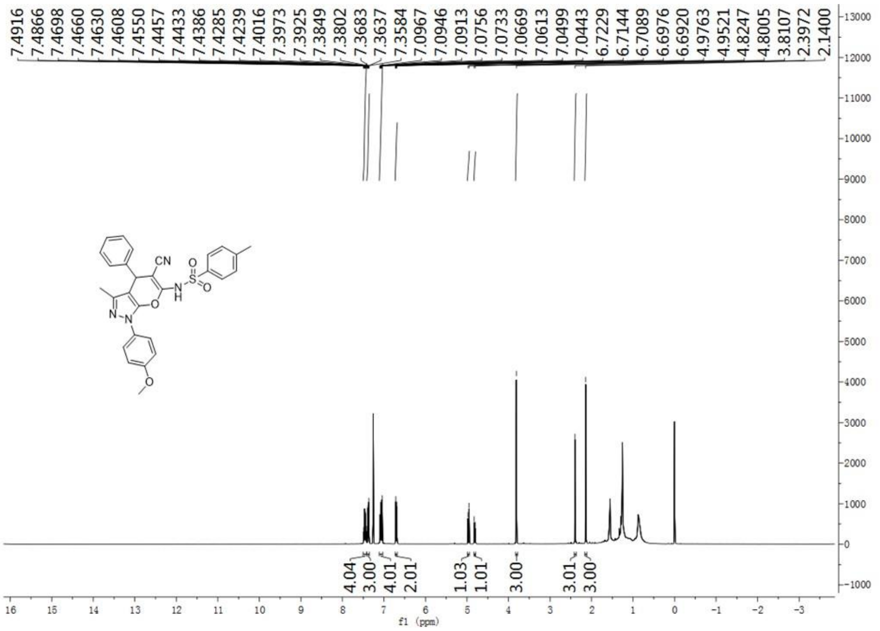
7.4916
7.4866
7.4698
7.4660
7.4630
7.4608
7.4550
7.4457
7.4433
7.4386
7.4285
7.4239
7.4016
7.3973
7.3925
7.3849
7.3802
7.3683
7.3637
7.3584
7.0967
7.0946
7.0913
7.0756
7.0733
7.0669
7.0613
7.0499
7.0443
6.7229
6.7144
6.7089
6.6976
6.6920
4.9763
4.9521
4.8247
4.8005
3.8107
2.3972
2.1400
4.04
3.00
4.01
2.01
1.03
1.01
3.00
3.01
3.00
f1 (ppm)

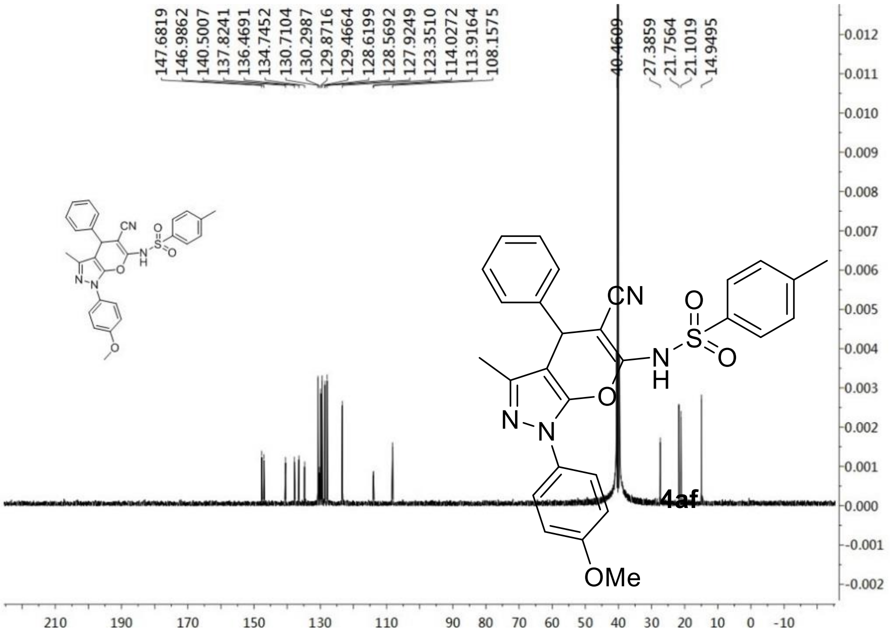
147.6819
146.9862
140.5007
137.8241
136.4691
134.7452
130.7104
130.2987
129.8716
129.4664
128.6199
128.5692
127.9249
123.3510
114.0272
113.9164
108.1575
40.4609
27.3859
21.7564
21.1019
14.9495
CN
O
S
O
N
H
N
N
O
OMe
4af
210
190
170
150
130
110
90
80
70
60
50
40
30
20
10
0
-10
0.012
0.011
0.010
0.009
0.008
0.007
0.006
0.005
0.004
0.003
0.002
0.001
0.000
-0.001
-0.002

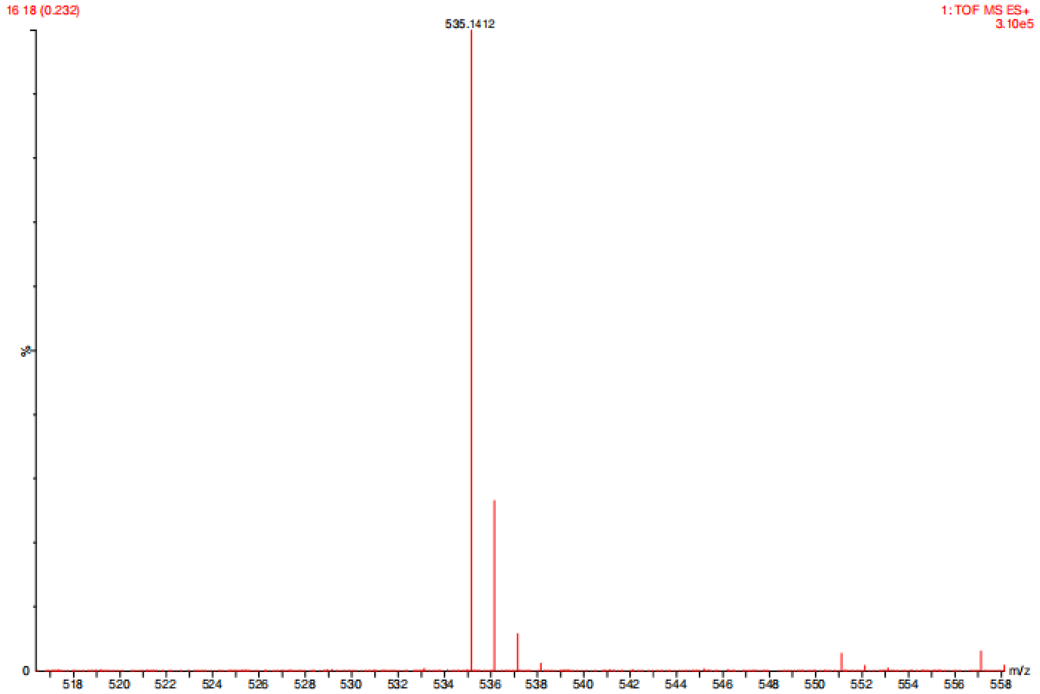
16 18 (0.232)
535.1412
1: TOF MS ES+
3.10e5
%
0
m/z
518
520
522
524
526
528
530
532
534
536
538
540
542
544
546
548
550
552
554
556
558

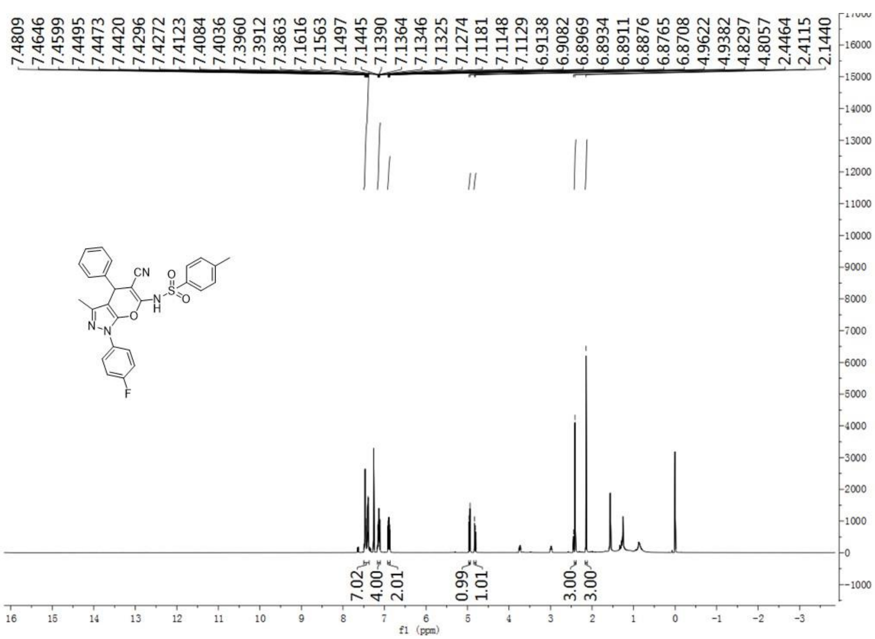
7.4809
7.4646
7.4599
7.4495
7.4473
7.4420
7.4296
7.4272
7.4123
7.4084
7.4036
7.3960
7.3912
7.3863
7.1616
7.1563
7.1497
7.1445
7.1390
7.1364
7.1346
7.1325
7.1274
7.1181
7.1148
7.1129
6.9138
6.9082
6.8969
6.8934
6.8911
6.8876
6.8765
6.8708
4.9622
4.9382
4.8297
4.8057
2.4464
2.4115
2.1440
CN
O
S
N
H
O
N
N
O
F
7.02
4.00
2.01
0.99
1.01
3.00
3.00
f1 (ppm)

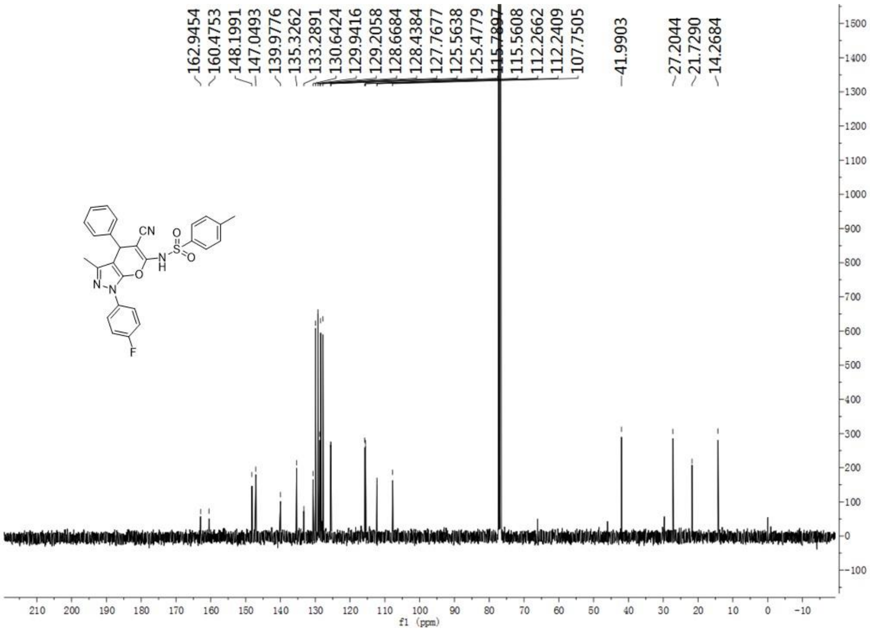
162.9454
160.4753
148.1991
147.0493
139.9776
135.3262
133.2891
130.6424
129.9416
129.2058
128.6684
128.4384
127.7677
125.5638
125.4779
115.7897
115.5608
112.2662
112.2409
107.7505
41.9903
27.2044
21.7290
14.2684
CN
O
S
N
H
O
N
N
O
F
f1 (ppm)

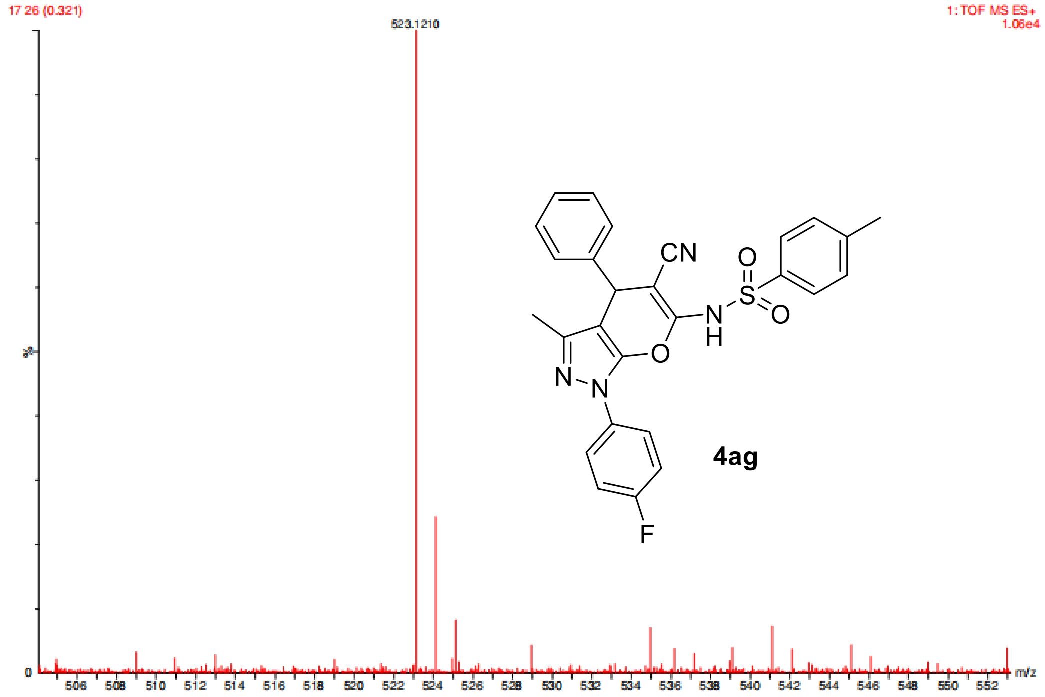
17 26 (0.321)
523.1210
1: TOF MS ES+
1.06e4
%
0
m/z
CN
O
S
O
N
H
N
N
O
F
4ag
